# Synthesis and Antibacterial Activity of Difluoromethyl Cinnamoyl Amides

**DOI:** 10.3390/molecules25040789

**Published:** 2020-02-12

**Authors:** Mario David Martínez, Diego Ariel Riva, Cybele Garcia, Fernando Javier Durán, Gerardo Burton

**Affiliations:** 1 CONICET-Universidad de Buenos Aires, UMYMFOR, Buenos Aires C1428EGA, Argentina; zenitramdm@gmail.com; 2 Departamento de Química Orgánica, Facultad de Ciencias Exactas y Naturales, Universidad de Buenos Aires, Buenos Aires C1428EGA, Argentina; 3 CONICET-Universidad de Buenos Aires, IQUIBICEN, Buenos Aires C1428EGA, Argentina; diegor@qb.fcen.uba.ar (D.A.R.); cybele.garcia@gmail.com (C.G.); 4 Departamento de Química Biológica, Facultad de Ciencias Exactas y Naturales, Universidad de Buenos Aires, Buenos Aires C1428EGA, Argentina

**Keywords:** difluoromethyl group, cinnamic acid amides, antibacterial

## Abstract

Series of novel amides of isoferulic acid, where the phenolic hydroxyl was replaced by a difluoromethyl group, were synthesized and their in vitro antibacterial activities assayed against fourteen bacterial strains (six Gram-positive and eight Gram-negative). A one-pot methodology was developed to obtain the 3′-(difluoromethyl)-4′-methoxycinnamoyl amides using Deoxofluor^®^ as a fluorinating agent. The *N*-isopropyl, *N*-isopentyl, and *N*-(2-phenylethyl) amides **11b**, **11d** and **11g** were the most active and selective against *Mycobacterium smegmatis* (MIC = 8 µg/mL) with **11b** and **11g** displaying negligible or no cytotoxicity against HepG2 and A549 cells. Thirteen analogs of *N*-isopropylamide **11b** were also synthesized and their antibacterial activity assayed. Results show that the difluoromethyl moiety enhanced antibacterial activity and selectivity towards *M. smegmatis,* changing the microorganism inhibition profile of the parent compound. The selectivity exhibited by some of the compounds towards *M. smegmatis* makes them potential leads in the search for new narrow spectrum antibiotics against *M. tuberculosis*.

## 1. Introduction

In the last 20 years, antimicrobial resistance has been recognized as a serious public health problem. The increased resistance of pathogenic microorganisms is related to the misuse/abuse of antibiotics and to their natural adaptation and evolution to marketed antimicrobial compounds (e.g., via biofilm formation, gene transfer from resistant counterparts, efflux pumps, cellular permeability, enzymes that confer resistance, and natural evolutionary mutations) [1,2,3]. This dangerous rise of pathogenic bacteria that are resistant to existing antibiotics constitutes a global human health threat and requires a continuous search for new chemical entities [4,5]. Broad spectrum antibiotics, effective with a wide range of pathogens, are important for first line treatment of bacterial infections as well as for prevention in risk situations (e.g., surgical procedures, organ transplant, etc.). Their use however damages the gut microbiota and favors the development of resistance mechanisms that may be readily transferred across species. Hence, once the pathogen has been identified, narrow spectrum therapy is recommended to minimize these adverse effects [6]. This requires the search for narrow spectrum antibiotics as well as uncovering the factors that influence selectivity towards specific bacteria.

**Figure 1:**
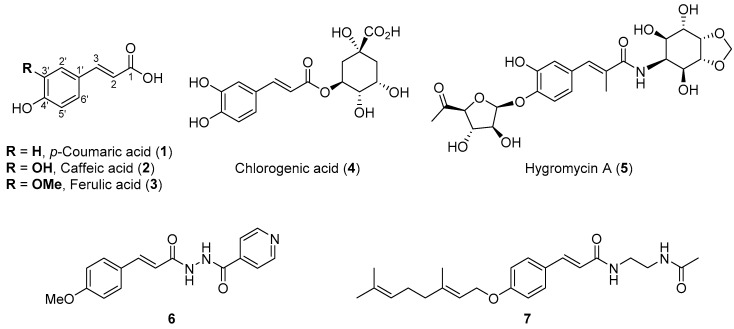
Natural and synthetic HCAs showing antibacterial activity.

Hydroxycinnamic acid derivatives (HCAs) have been shown to possess in vitro antibacterial features as well as health benefits [7,8,9]. HCAs possess a hydroxylated and/or methoxylated 3-phenylpropenoyl core (C_6_–C_3_) and are one of the most plentiful and ubiquitously distributed groups of plant secondary metabolites, commonly found in a variety of dietary products such as vegetables, fruits, chocolate, and beverages. The most common HCAs found in fruits and vegetables are *p*-coumaric (**1**), caffeic (**2**), ferulic (**3**), and chlorogenic (**4**) acids (Figure 1). Only a minor proportion of HCAs are present in their free form, and most are found as quinic or tartaric acid derivatives (e.g., chlorogenic acid **4** or hygromycin A **5**). Daily intake of HCAs ranges from 25 to 1000 mg depending on the type of diet. Some of the most abundant sources of these compounds are coffee beans and *yerba mate* brews [9,10]. Surprisingly, the HCAs molecular target in terms of their antibacterial activity is still unknown, but in some cases experimental evidence has proven the interaction of these types of compounds with the pathogen cell membrane [11,12,13,14].

The bioavailability of HCAs and their metabolites is crucial for their pharmacological properties; however, HCAs exhibit poor ADME (absorption, distribution, metabolism, and excretion) properties showing a rapid phase II metabolic transformation (e.g., methylation, glucuronidation, and sulfation) followed by urine and feces excretion. This leads to poor translation of in vitro features to in vivo therapeutic applications of HCAs [15]. Several attempts have been made to enhance the bioavailability and antibacterial selectivity of HCAs by means of lipophilization strategies such as amidation or esterification of the acyl moiety and functionalization of the phenolic hydroxyl with long alkyl or prenyl chains [13,16,17,18]. The latter approach has the disadvantage of poor atom economy and may also lead to self-aggregation and internalization to the lipidic core. Synthetic amides derived from HCAs have shown the most interesting antibacterial properties with several of the hydroxycinnamoyl amides and structurally related analogs being active against *Mycobacterium tuberculosis* including resistant strains (e.g., compounds **6** and **7**, Figure 1) [19,20]. Some structural aspects relevant to the antibacterial potency and selectivity are the oxygen atom at C-4′ (free hydroxyl or alkoxy substituent) and the nitrogen atom at C-1 (amides, hydrazides, and nitrogen heterocycles) [7,8,21]. In this context, alternate approaches for increasing lipophilicity may provide access to new lead compounds with improved activities.

The difluoromethyl group has been shown to exhibit some distinctive properties that make it an interesting alternative to the widely used trifluoromethyl group when seeking to increase lipophilicity. In particular, due to its hydrogen bond donor properties, the CF_2_H group has been proposed as a lipophilic bioisostere of hydroxyl groups [22,23]. Recently, Zafrani and coworkers showed that, when bonded to an aryl scaffold, the difluoromethyl group effectively behaves as a lipophilic bioisoster of the phenolic OH, increasing lipophilicity while retaining significant hydrogen bonding acidity [24]. Hydrogen bonding acceptor properties of the difluoromethyl group have also been reported [25]. Previously, we also found an interesting bioisosteric relationship in the antioxidant behavior between difluoromethyl substituted arenes and phenols. Thus, replacement of a phenolic hydroxyl by a difluoromethyl moiety on hydroxycinnamic acid methyl ester scaffolds conferred radical scavenging ability only in lipophilic environments, even in the absence of free phenol groups, probably due to a hydrogen atom transfer mechanism [26]. We envisaged that the combination of this lipophilicity-booster fluorinated moiety together with the amidation strategy applied to HCAs could lead to analogs with enhanced ADME properties, with the added possibility of perturbing the bacterial redox homeostasis beyond the effects over the bacterial cell wall. Here, we report the synthesis of a series of difluoromethyl substituted methoxycinnamoyl amides and their biological evaluation against a panel of fourteen relevant microorganisms (Gram-positive and Gram-negative bacteria).

## 2. Results and Discussion

**Figure 2:**
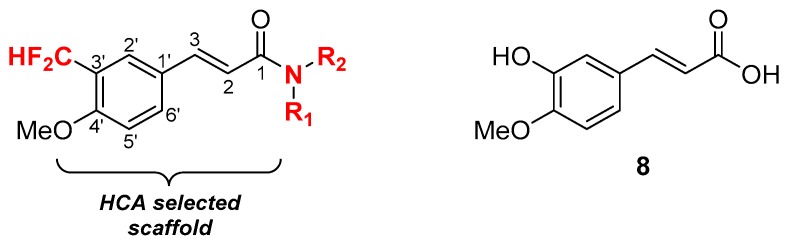
Proposed modifications (in red) to the coumaric acid (**1**) scaffold and analogy with isoferulic acid (**8**).

Our initial approach was to introduce a difluoromethyl moiety at the C-3′ position of a 4′-methoxycinnamoyl amide core and to evaluate the effect of the resulting analogs of isoferulic acid (**8**) on antibacterial activity (Figure 2). The difluoromethyl moiety was expected to enhance the lipophilicity while retaining, at least in part, the hydrogen bonding donor/acceptor capacity of the phenolic hydroxyl as well as its radical scavenging properties. The 4′-methoxy group was expected to provide the required oxygen atom with metabolic stability and to prevent formation of an *o*-quinomethane [27], while the acyl amide would confer antibacterial potency and selectivity, as well as allow fine tuning of drug-like properties by modifying the substituents on the nitrogen atom. This approach does not introduce major steric changes in the aromatic ring vicinity compared with caffeic acid and related HCAs.

### 2.1. -CF_2_H at C-3′ on the 4′-methoxycinnamyl Amide Scaffold

**Scheme 1:**
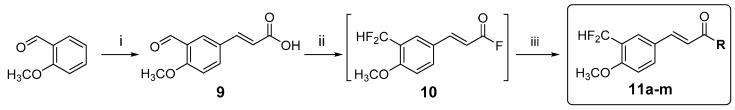
Synthesis of amides **11a**–**11m**. Reagents and conditions: (i) (1) AgNO_3_, I_2_, MeOH, (2) Pd(AcO)_2_, (*o*-Tol)_3_P, methyl acrylate, Et_3_N, MeCN, and (3) K_2_CO_3_, MeOH-H_2_O; (ii) Deoxofluor^®^, PhCH_3_-CH_2_Cl_2_; and (iii) R_1_R_2_NH or R_1_R_2_NH·HX (see Table 1 for details).

The synthetic strategy (Scheme 1) aimed to access, in a straightforward fashion, a variety of amides with the general structure **11**. Commercially available 2-methoxybenzaldehyde was iodinated with iodine and silver nitrate in methanol and the resulting 5-iodo-2-methoxybenzaldehyde was then coupled with methyl acrylate under Heck reaction conditions using palladium acetate and tri(*o*-tolyl)phosphine as a ligand [28,29] to give the cinnamic acid derivative **9** in 73% isolated yield after alkaline hydrolysis. The structure of compound **9** was confirmed by ^1^H- and ^13^C-NMR spectra that showed resonances at δ_H_/δ_C_ 12.35/167.6 for the carboxylic acid and at δ_H_/δ_C_ 10.33/188.9 for the aldehyde moiety. The side chain double bond gave signals at δ_H_/δ_C_ 7.59/142.6 and 6.47/118.3, with a ^1^H-^1^H coupling constant of 16 Hz that confirmed the *E* configuration.

The key step in this synthetic approach was the double deoxofluorination of the cinnamic acid derivative **9** to give 3′-difluoromethyl-4′-methoxycinnamoyl fluoride (**10**) and its subsequent conversion to amides **11a**–**11m** in a one pot procedure (Table 1). At this stage, Deoxofluor^®^ was chosen as the fluorinating reagent, as initial attempts to use XtalFluor-E^®^ were unsuccessful due to long reaction times and undesired byproducts [30]. However, one drawback of using Deoxofluor^®^ is the formation of *N,N*-bis(2-methoxyethyl)amine as a byproduct that can react with the acyl fluoride (Table 1, Method B), thus limiting the scope of the method to amines that are more nucleophilic than *N,N*-bis(2-methoxyethyl)amine [31,32,33,34]. We found that this undesired condensation could be avoided by filtering the reaction mixture through a short silica gel pad. The resulting solution containing the acyl fluoride **10** was concentrated and treated directly with the desired amine and triethylamine as a base (Table 1, Methods A and D). The intermediate acyl fluoride could also be isolated upon evaporation of the solvent and was characterized by ^1^H and ^13^C-NMR. This methodology allowed for the synthesis of primary and secondary amides and was also applicable to aromatic amines (e.g., aniline). Amines could be used either as free bases or as their salts (e.g., hydrochlorides and acetates), and even the highly unreactive amine 2,5-bis(trifluoromethyl)aniline gave the corresponding amide when the reaction was carried out in the presence of LDA as a base (Table 1, Method C). In the latter case, after filtration of the acyl fluoride solution, the solvent was completely removed under reduced pressure and replaced by THF. In contrast with previous reports [31,33], this procedure allows for the coupling of a wide variety of amines (either free or as their salts) with acyl fluorides, employing Deoxofluor^®^ as the fluorinating agent. The procedure was easily scalable allowing the synthesis of several amides in parallel. Following this procedure, 13 amides of 3′-difluoromethyl-4′-methoxycinnamic acid (**11a**–**11m**) were synthesized in 46–84% isolated yields (Table 1). Structures were confirmed by ^1^H and ^13^C-NMR (1D and 2D) and mass spectrometry. Particularly diagnostic were the shifts of the -CF_2_H group at ca. δ_H_ 6.9 (triplet, *J*_HF_ = 55.5 Hz) and δ_C_ 111.3 (triplet, *J*_CF_ = 236 Hz).

The series of amides **11a**–**11m** was screened for antibacterial activity against a panel of 14 microorganisms: six Gram-positive and eight Gram-negative bacteria. The most susceptible microorganism was the Gram-positive bacillus *Mycobacterium smegmatis* (Table 2) with compounds **11b**, **11d** and **11g** being the most promising candidates in terms of potency (MICs = 8 µg/mL) and selectivity. It has been shown that *M. smegmatis* can be used as a non-pathogenic model for *M. tuberculosis* in antibacterial screening, as activity against *M. smegmatis* usually results in activity against *M. tuberculosis* [35]. Marginal bacterial growth inhibition was detected for other compounds in the series against various microorganisms such as *Streptococcus pyogenes, Streptococcus pneumoniae, Clostridum sporogenes, Pseudomona aeruginosa*, and *Acinetobacter guillouiae* (MICs = 64 µg/mL). To carry out preliminary SAR studies, compound **11b** was chosen as the better candidate due to its potency, selectivity towards *M. smegmatis*, and atom economy.

### 2.2. Structure Activity Relationship Studies (SAR) on Compound ***11b***

**Scheme 2:**
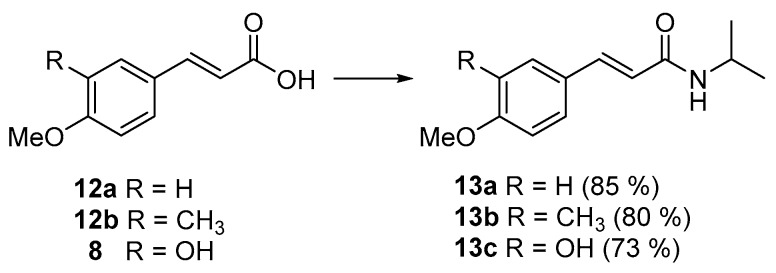
Synthesis of amides **13a**–**c**. Reagents and conditions: BOP, Et_3_N, CH_2_Cl_2_, *i*-PrNH_2_, 0–30 °C.

To evaluate the influence of the 3′-CF_2_H moiety on antibacterial activity, we synthesized compounds **13a**–**13c** by condensation of the corresponding acids **12a**, **12b**, or **8** and isopropylamine using (benzotriazol-1-yloxy)tris(dimethylamino)phosphonium hexafluorophosphate (BOP) as the activating agent (Scheme 2) [36]. While the bioisosteric replacement of a hydroxyl group by CF_2_H takes advantage of the hydrogen bonding donor (and possibly acceptor) ability of the CF_2_H to mimic the hydroxyl, replacement of a methyl group by CF_2_H makes use of the fluorine as a bioisostere of hydrogen due to its relatively small size. The CF_2_H has been shown to increase lipophilicity relative to the hydroxyl group (thus the lipophilic hydroxyl analogy) but to reduce lipophilicity when compared to the methyl analog [24].

**Scheme 3:**
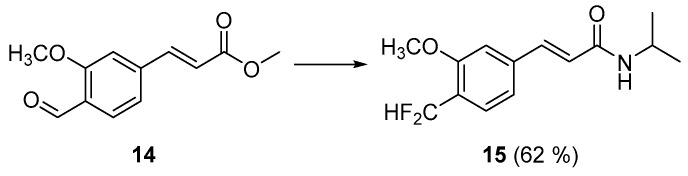
Synthesis of amide **15**. Reagents and conditions: (1) K_2_CO_3_, MeOH-H_2_O; (2) Deoxofluor^®^, PhCH_3_-CH_2_Cl_2_; and (3) *i*-PrNH_2_, Et_3_N.

Both the non-functionalized counterpart **13a** and the 3′-methyl analog **13b** were only moderately active against *M. smegmatis*, with a four-fold activity decrease compared to the difluoromethylated analog **11b** (see Table 2). On the other hand, the isoferulic acid amide **13c** with a phenolic hydroxyl at position 3′ showed a different activity profile, namely that it was inactive against *M. smegmatis* but moderately active against the Gram-negative bacteria *Shigella boydii* and *Shigella flexneri*. These results suggest that the balance between lipophilicity (see Table S1) and hydrogen bond donor/acceptor capacities of the CF_2_H moiety is a significant contributor to the observed activity and selectivity towards *M. smegmatis* of the *N*-isopropylamide derivative. Furthermore, to analyze the aromatic ring substitution pattern, the regioisomer **15** was obtained from the methyl cinnamate **14** (Scheme 3) [26]. Compound **15** presented a different antibacterial activity profile (MIC = 32 µg/mL against *Clostridium sporogenes*, see Table 2) compared to compound **11b**, proving the importance of the substitution pattern in the selective activity against *Mycobacterium smegmatis*.

**Scheme 4:**
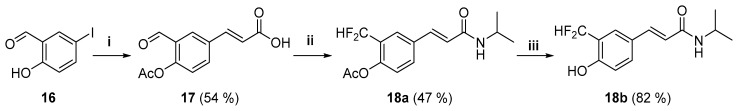
Synthesis of amides **18a** and **18b**. Reagents and conditions: (i) (1) Ac_2_O, K_2_CO_3_, acetone and (2) Pd(AcO)_2_, (*o*-Tol)_3_P, acrylic acid, Et_3_N, MeCN; (ii) (1) Deoxofluor^®^, PhCH_3_-CH_2_Cl_2_ and (2) *i*-PrNH_2_, Et_3_N-CH_2_Cl_2_; and (iii) H_2_SO_4_, MeOH.

To evaluate the role of the methoxy group at the 4′ position of the aromatic ring, acetate **18a** and the free phenol **18b** were synthesized (Scheme 4). Compounds **18a** and **18b** showed a significant loss of potency (Table 2) and, in the case of **18a**, an increased cytotoxicity (see below).

**Figure 3:**
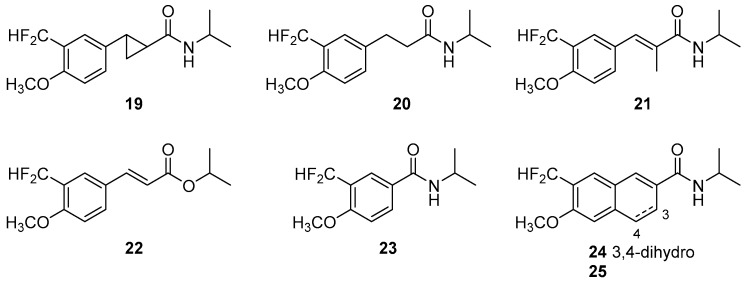
Analogs of **11b** with modified side chains.

We then focused our attention on the importance of the alkenoyl chain at position C1′ (i.e., hybridization, planarity, and length). Chain modified analogs **19**–**25** (Figure 3) were obtained with minor modifications to the synthetic strategy used for compound **11b**. Briefly, the cyclopropyl analog **19** was obtained by a sequence involving a Corey–Chaykovsky reaction over the Weinreb amide **11h**, followed by a Gassman’s “anhydrous hydroxide” saponification and a BOP condensation with isopropylamine [37,38]. Compound **20** was synthesized by catalytic hydrogenation of **11b**. Compound **21** was obtained by replacing acrylic acid with methacrylic acid in the Heck reaction and ester **22** was obtained by treating the acyl fluoride **10** with isopropanol instead of isopropylamine. The vinylogue **23** was obtained by a Rieche formylation of commercial *p*-methoxybenzoic acid followed by the deoxofluorination/condensation protocol used for the synthesis of **11b** [39,40]. Naphthalene derivatives **24** and **25** were obtained in a similar way as **23**. Compounds **19**–**25** showed loss of activity and selectivity, proving the importance of the structural features of the side chain and validating the hydroxycinnamic acid scaffold as a privileged structure.

Finally, all compounds active against *M. smegmatis* were evaluated for cytotoxicity in human lung carcinoma A549 cells and hepatoma HepG2 cells. Compound **13c** was also included due to its activity against the Gram-negative bacteria *S. boydii* and *S. flexneri* (Table 3). Only compound **18a** exhibited significant cytotoxicity against both cell lines, while compound **11d** was moderately cytotoxic for HepG2 cells. Two of the most active compounds, **11b** and **11g**, had selectivity indexes of 24.9 or higher.

## 3. Materials and Methods 

### 3.1. General

Melting points were taken on a Fisher–Johns apparatus and are uncorrected. ^1^H- and ^13^C-NMR spectra were measured at 500.13 and 125.76 MHz, respectively, on a Bruker Avance II 500 NMR spectrometer unless noted otherwise; J values are given in Hz. All assignments were confirmed by a combination of 2D spectra (COSY, HSQC, and HMBC). Elemental analysis (C, H, and N) was performed on an Exeter Analytical Inc. CE-440 apparatus, North Chelmsford, MA, USA. The electron impact mass spectra (EIMS) were measured on a Shimadzu QP-5000 or a Thermo DSQ-II mass spectrometer at 70 eV by direct inlet. Exact mass spectra (HRMS) were measured on a Bruker micrOTOF-Q II mass spectrometer using positive electrospray ionization. Analytical thin layer chromatography (tlc) was performed on pre-coated silica gel plates (Merck F254, 0.2 mm thickness); compounds were visualized under 254 nm UV light. Flash column chromatography was performed on silica gel Merck 9385 (0.0040–0.0063 mm). All solvents were distilled and stored over 4 Å molecular sieves before use. Solvents were evaporated at ca. 45 °C under vacuum in a rotary evaporator. The homogeneity of all compounds was confirmed by tlc. Products obtained as solids or syrups were dried under high vacuum. 5-Iodo-2-methoxybenzaldehyde was obtained by iodination of 2-methoxybenzaldehyde with iodine/silver nitrate in methanol [28]. Methyl-3-[4-formyl-3-methoxyphenyl]-(*E*)-propenoate (**14**) was obtained as described previously [26]. 6-Methoxy-3,4-dihydronaphthalene-2-carboxylic acid was obtained from commercial 6-methoxytetralone [41].

### 3.2. Chemical Synthesis

#### 3.2.1. 3-(3-Formyl-4-methoxyphenyl)-(*E*)-propenoic acid (**9**) 

5-Iodo-2-methoxy-benzaldehyde (1.0 g, 3.82 mmol) was dissolved in 25 mL of acetonitrile and oxygen was removed by bubbling nitrogen through the solution. Triethylamine (3.71 mL, 26.7 mmol) and methyl acrylate (0.686, mL, 7.63 mmol) were added dropwise with stirring to the solution, followed by four portions of tri-*o*-tolylphosphine (0.0214 g, 0.095 mmol) and palladium (II) acetate (0.008 g, 0.047 mmol) at 1 h intervals. The mixture was then heated at 65–70 °C for 4 h, volatiles were removed by distillation, and the residue was dissolved in dichloromethane. The resulting solution was percolated through silica gel, eluting with a mixture of hexane–ethyl acetate (6:4). The percolate was evaporated to dryness, re-dissolved in 20 mL of methanol, and 20% aqueous potassium carbonate solution (10 mL) added. The mixture was stirred for 6 h at 20 °C and concentrated under reduced pressure to a third of its volume. The solution was acidified with conc. HCl (to pH 1) and the precipitate was filtered and recrystallized from isopropanol, to give 3-formyl-4-methoxycinnamic acid **9** as a crystalline white solid (0.472 g). A second harvest obtained by concentration of the mother liquor gave an additional 0.105 g (73%); m.p. 223–225 °C; ^1^H-NMR (DMSO-d_6_) δ: 12.35 (s, 1H, COO**H**), 10.33 (s, 1H, ArC**H**O), 8.03 (dd, *J* = 2.4, 8.8 Hz, 1H, 6′-H), 7.93 (d, *J* = 2.4 Hz, 1H, 2′-H), 7.59 (d, *J* = 16.0 Hz, 1H, 3-H), 7.28 (d, *J* = 8.8 Hz, 1H, 5′-H), 6.47 (d, *J* = 16.0 Hz, 1H, 2-H), 3.96 (s, 3H, C**H_3_**O); ^13^C-NMR (DMSO-d_6_) δ: 188.9 (Ar**C**HO), 167.5 (1-C), 162.6 (4′-C), 142.6 (3-C), 135.6 (6′-C), 128.2 (2′-C), 126.9 (1′-C), 124.2 (3′-C), 118.3 (2-C), 113.4 (5′-C), 56.4 (**C**H_3_O). EIMS *m*/*z* (%): 206 (18, M^+^), 81 (33), 69 (100), 57 (25), 55 (33), 43 (39), 41 (58).

#### 3.2.2. 3-[3-(difluoromethyl)-4-methoxyphenyl]-(*E*)-propenoyl fluoride (**10**)

A solution of Deoxofluor^®^ 50% in toluene (0.107 mL, 0.435 mmol) was added dropwise to a suspension of 0.030 g of 3-formyl-4-methoxycinnamic acid (**9**, 0.030 g, 0.145 mmol) in 0.4 mL of dry dichloromethane under an argon atmosphere. The reaction mixture was stirred for 45 min at room temperature, diluted with 2 mL of dry dichloromethane and subsequently percolated through 2.5 g of silica gel under an argon atmosphere. The silica gel bed was rinsed with 8 mL of dry dichloromethane and the resulting solution was concentrated to dryness. The resulting solid was purified by flash column chromatography eluting with mixtures of ethyl acetate–hexane of increasing polarity to give **10** as a white solid (0.028 g, 84%). ^1^H-NMR (CDCl_3_, 200.13 MHz) δ: 7.80 (d, *J* = 16.0 Hz, 1H, 3-H), 7.79 (bs, 1H, 2′-H), 7.64 (m, 1H, 6′-H), 7.00 (m, 1H, 5′-H), 6.93 (t, *J* = 55.3 Hz, 1H, CF_2_**H**), 6.29 (dd, *J* = 7.1, 16.0 Hz, 1H, 2-H), 3.94 (s, 3H, C**H_3_**O); ^13^C-NMR (CDCl_3_, 50.32 MHz) δ: 157.2 (d, *J* = 338 Hz, 1-C), 159.9 (t, *J* = 6 Hz, 4′-C), 150.1 (d, *J* = 6 Hz, 3-C), 133.0 (s, 6′-C), 126.7 (t, *J* = 6.0 Hz, 2′-C), 125.9 (s, 1′-C), 123.7 (t, *J* = 22 Hz, 3′-C), 111.5 (s, 5′-C), 110.8 (t, *J* = 236 Hz, **C**F_2_H), 110.7 (d, *J* = 68 Hz, 2-C), 56.0 (s, **C**H_3_O).

#### 3.2.3. Representative Procedure. Preparation of *N*-(1-Methylethyl)-3-[3-(difluoromethyl)-4-methoxyphenyl]-(*E*)-propenamide (**11b**)

A solution of Deoxofluor^®^ 50% in toluene (0.107 mL, 0.435 mmol) was added dropwise to a suspension of 0.030 g of 3-formyl-4-methoxycinnamic acid (**9**, 0.030 g, 0.145 mmol) in 0.4 mL of dry dichloromethane under an argon atmosphere. The reaction mixture was stirred for 45 min at room temperature, diluted with 2 mL of dry dichloromethane, and subsequently percolated through 2.5 g of silica gel under an argon atmosphere. The silica gel bed was rinsed with 8 mL of dry dichloromethane and the resulting solution was concentrated under a nitrogen flow to a final volume of 1 mL. Isopropylamine (0.037 mL, 0.435 mmol) and triethylamine (0.061 mL, 0.435 mmol) were added and the mixture was subsequently stirred for 1 h at room temperature. The solution was diluted with 10 mL of dichloromethane, washed twice with 1M HCl and once with water, dried with anhydrous sodium sulfate, and the solvent evaporated. The resulting solid was purified by flash column chromatography eluting with mixtures of ethyl acetate–hexane of increasing polarity to give **11b** as a crystalline white solid (0.025 g, 64%). m.p. 113–114 °C; ^1^H-NMR (CDCl_3_) δ: 7.75–7.71 (m, 1H, 2′-H), 7.57 (d, *J* = 15.5 Hz, 1H, 3-H), 7.53–7.49 (m, 1H, 6′-H), 6.92 (t, *J* = 55.5 Hz, 1H, CF_2_**H**), 6.94–6.88 (m, 1H, 5′-H), 6.31 (d, *J* = 15.6 Hz, 1H, 2-H), 5.60–5.54 (m, N**H**), 4.29 – 4.16 (m, 1H, 1′′-H), 3.89 (s, 3H, C**H_3_**O), 1.22 (d, *J* = 6.5 Hz, 6H, 2′′-H); ^13^C-NMR (CDCl_3_) δ: 165.1 (s, 1-C), 158.3 (t, *J* = 6.3 Hz, 4′-C), 139.6 (s, 3-C), 132.4 (t, *J* = 2.1 Hz, 6′-C), 127.8 (s, 1′-C), 125.0 (t, *J* = 5.9 Hz, 2′-C), 123.2 (t, *J* = 22.2 Hz, 3′-C), 120.1 (s, 2-C), 111.3 (t, *J* = 236.3 Hz, **C**F_2_H), 111.3 (s, 5′-C), 56.0 (s, **C**H_3_O), 41.7 (s, 1′′-C), 23.0 (s, 2′′-C). EIMS *m*/*z* (%): 269 (57, M^+^), 211 (100), 183 (23), 132 (19), 58 (34). Analysis for C_14_H_17_F_2_NO_2_: Calcd: C, 62.42; H, 6.36; N, 5.20%. Found: C, 62.45; H, 6.32; N, 4.98%.

#### 3.2.4. *N*-Methyl-3-[3-(difluoromethyl)-4-methoxyphenyl]-(*E*)-propenamide (**11a**)

Compound **11a** was prepared from acid **9** (30.0 mg, 0.145 mmol), Deoxofluor^®^ 50% in toluene (0.107 mL, 0.435 mmol), methylamine hydrochloride (0.030 g, 0.435 mmol), and triethylamine (0.082 mL, 0.580 mmol) following the procedure described for **11b**. Compound **11a** was obtained as a crystalline white solid (0.021 g, 60 %); m.p. 169–171 °C; ^1^H-NMR (CDCl_3_-CD_3_OD 9:1) δ: 7.76–7.71 (m, 1H, 2′-H), 7.57–7.54 (m, 1H, 6′-H), 7.54 (d, *J* = 15.5 Hz, 1H, 3-H), 6.97–6.93 (m, 1H, 5′-H), 6.93 (t, *J* = 55.5 Hz, 1H, CF_2_**H**), 6.40 (d, *J* = 15.7 Hz, 1H, 2-H), 3.90 (s, 3H, C**H_3_**O), 2.89 (s, 3H, NC**H_3_**); ^13^C-NMR (CDCl_3_-CD_3_OD 9:1) δ: 167.5 (1-C), 158.2 (t, *J*_CF_ = 5.7 Hz, 4′-C), 139.4 (3-C), 132.2 (6′-C), 127.6 (1′-C), 125.0 (t, *J*_CF_ = 5.9 Hz, 2′-C), 123.0 (t, *J*_CF_ = 22.2 Hz, 3′-C), 119.3 (2-C), 111.2 (t, *J*_CF_ = 236.1 Hz, **C**F_2_H), 111.2 (5′-C), 55.8 (**C**H_3_O), 26.2 (N**C**H_3_). EIMS *m*/*z* (%): 241 (55, M^+^), 240 (26), 211 (100), 183 (25), 132 (22). Analysis for C_12_H_13_F_2_NO_2_: Calcd: C, 59.75; H, 5.43; N, 5.81 %. Found: C, 59.27; H, 5.28; N, 5.66 %.

#### 3.2.5. *N*-(2-Methylpropyl)-3-[3-(difluoromethyl)-4-methoxyphenyl]-(*E*)-propenamide (**11c**)

Compound **11c** was prepared from acid **9** (30.0 mg, 0.145 mmol), Deoxofluor^®^ 50% in toluene (0.107 mL, 0.435 mmol), isobutylamine (0.044 mL, 0.435 mmol), and triethylamine (0.061 mL, 0.435 mmol) following the procedure described for **11b**. Compound **11c** was obtained as a crystalline white solid (0.027 g, 66 %); m.p. 145–146 °C; ^1^H-NMR (CDCl_3_) δ: 7.77–7.72 (m, 1H, 2′-H), 7.59 (d, *J* = 15.5 Hz, 1H, 3-H), 7.56–7.49 (m, 1H, 6′-H), 6.93 (t, *J* = 55.5 Hz, 1H, CF_2_**H**), 6.94–6.90 (m, 1H, 5′-H), 6.35 (d, *J* = 15.5 Hz, 1H, 2-H), 5.74 (t, *J* = 5.4 Hz, 1H, N**H**), 3.89 (s, 3H, C**H_3_**O), 3.23 (dd, *J* = 6.2, 6.7 Hz, 2H, 1′′-H), 1.92–1.77 (1 H, m, 2′′-H), 0.96 (d, *J* = 6.7 Hz, 6H, 3′′-H); ^13^C-NMR (CDCl_3_) δ: 166.0 (1-C), 158.3 (t, *J*_CF_ = 5.7 Hz, 4′-C), 139.8 (3-C), 132.4 (6′-C), 127.8 (1′-C), 125.0 (t, *J*_CF_ = 5.9 Hz, 2′-C), 123.3 (t, *J*_CF_ = 22.3 Hz, 3′-C), 119.8 (2-C), 111.3 (t, *J*_CF_ = 236.2 Hz, **C**F_2_H), 111.3 (5′-C), 56.0 (**C**H_3_O), 47.2 (1′′-C), 28.8 (2′′-C), 20.3 (3′′-C). EIMS *m/z* (%): 283 (30, M^+^), 226 (53), 211 (100), 183 (22), 132 (20), 43 (18). Analysis for C_15_H_19_F_2_NO_2_·0.5H_2_O: Calcd: C, 61.63; H, 6.90; N, 4.79%. Found: C, 61.51; H, 6.57; N, 4.76%.

#### 3.2.6. *N*-(3-Methylbutyl)-3-[3-(difluoromethyl)-4-methoxyphenyl]-(*E*)-propenamide (**11d**) 

Compound **11d** was prepared from acid **9** (30.0 mg, 0.145 mmol), Deoxofluor^®^ 50% in toluene (0.107 mL, 0.435 mmol), isopentylamine (0.051 mL, 0.435 mmol), and triethylamine (0.061 mL, 0.435 mmol) following the procedure described for **11b**. Compound **11d** was obtained as a crystalline white solid (0.025 g, 58%); m.p. 110–111 °C; ^1^H-NMR (CDCl_3_) δ: 7.80–7.68 (m, 1H, 2′-H), 7.58 (d, *J* = 15.5 Hz, 1H, 3-H), 7.55–7.49 (m, 1H, 6′-H), 6.92 (t, *J* = 55.5 Hz, 1H, CF_2_**H**), 6.97–6.85 (m, 1H, 5′-H), 6.33 (d, *J* = 15.5 Hz, 1H, 2-H), 5.71 (t, *J* = 5.6 Hz, 1H, N**H**), 3.89 (s, 3H, C**H_3_**O), 3.50–3.33 (m, 2H, 1′′-H), 1.72–1.61 (1 H, m, 2′′-H), 1.46 (dt, *J* = 7.0, 8.5 Hz, 2H, 3′′-H), 0.94 (d, *J* = 6.6 Hz, 6H, 4′′-H); ^13^C-NMR (CDCl_3_) δ: 165.9 (1-C), 158.3 (t, *J*_CF_ = 5.7 Hz, 4′-C), 139.7 (3-C), 132.4 (t, *J*_CF_ = 1.9 Hz, 6′-C), 127.8 (1′-C), 125.0 (t, *J*_CF_ = 5.9 Hz, 2′-C), 123.2 (t, *J*_CF_ = 22.2 Hz, 3′-C), 119.9 (2-C), 111.3 (t, *J*_CF_ = 236.2 Hz, **C**F_2_H), 111.3 (5′-C), 56.0 (**C**H_3_O), 38.7 (2′′-C), 38.2 (1′′-C), 26.0 (3′′-C), 22.6 (4′′-C). EIMS *m*/*z* (%): 297 (30, M^+^), 241 (44), 240 (32), 226 (22), 211 (100), 183 (27), 132 (25). Analysis for. C_16_H_21_F_2_NO_2_: Calcd: C, 64.63; H, 7.12; N, 4.71%. Found: C, 64.48; H, 7.05; N, 4.74%.

#### 3.2.7. *N*-Cyclohexyl-3-[3-(difluoromethyl)-4-methoxyphenyl]-(*E*)-propenamide (**11e**)

Compound **11e** was prepared from acid **9** (30.0 mg, 0.145 mmol), Deoxofluor^®^ 50% in toluene (0.107 mL, 0.435 mmol), cyclohexylamine (0.050 mL, 0.435 mmol), and triethylamine (0.061 mL, 0.435 mmol) Following the procedure used for **11b**. Compound **11e** was obtained as a crystalline white solid (0.034 g, 76%); m.p. 181–183 °C; ^1^H-NMR (CDCl_3_) δ: 7.75–7.73 (m, 1H, 2′-H), 7.58 (d, *J* = 15.5 Hz, 1H, 3-H), 7.52 (m, 1H, 6′-H), 6.92 (t, *J* = 55.5 Hz, 1H, CF_2_**H**), 6.93–6.90 (m, 1H, 5′-H), 6.32 (d, *J* = 15.5 Hz, 1H, 2-H), 5.61 (d, *J* = 6.2 Hz, 1H, N**H**), 3.97–3.90 (m, 1H, 1′′-H), 3.89 (s, 3H, C**H_3_**O), 2.04–1.92 (m, 2H, 2′′-H*_eq_*), 1.79–1.69 (m, 2H, 3′′-H*_eq_*), 1.68–1.59 (m, 1H, 4′′-H*_eq_*), 1.48–1.34 (m, 2H, 3′′-H*_ax_*), 1.29–1.11 (m, 3H, 2′′-H*_ax_* and 4′′-H*_ax_*); ^13^C-NMR (CDCl_3_) δ: 165.0 (1-C), 158.3 (t, *J*_CF_ = 5.6 Hz, 4′-C), 139.6 (3-C), 132.4 (6′-C), 127.9 (1′-C), 125.0 (t, *J*_CF_ = 5.8 Hz, 2′-C), 123.3 (t, *J*_CF_ = 22.3 Hz, 3′-C), 120.2 (2-C), 111.3 (t, *J*_CF_ = 236.2 Hz, **C**F_2_H), 111.3 (5′-C), 56.0 (**C**H_3_O), 48.5 (1′′-C), 33.4 (2′′-C), 25.7 (4′′-C), 25.0 (3′′-C). EIMS *m*/*z* (%): 309 (62, M^+^), 226 (64), 211 (100), 183 (33), 132 (25), 98 (39). Analysis for C_17_H_21_F_2_NO_2_: Calcd C, 66.00; H, 6.84; N, 4.53%. Found: C, 65.85; H, 6.86; N, 4.36%.

#### 3.2.8. *N*-Phenyl-3-[3-(difluoromethyl)-4-methoxyphenyl]-(*E*)-propenamide (**11f**)

Compound **11f** was prepared from acid **9** (30.0 mg, 0.145 mmol), Deoxofluor^®^ 50% in toluene (0.107 mL, 0.435 mmol), aniline (0.040 mL, 0.435 mmol), and triethylamine (0.061 mL, 0.435 mmol) following the procedure used for **11b**. Compound **11f** was obtained as a crystalline white solid (0.025 g, 57%); m.p. 138–139 °C; ^1^H-NMR (CDCl_3_) δ: 7.76 (m, 1H, 2′-H), 7.70 (d, *J* = 15.5 Hz, 1H, 3-H), 7.63 (d, *J* = 7.2 Hz, 2H, 2′′-H), 7.60 (bs, 1H, N**H**), 7.54–7.50 (m, 1H, 6′-H), 7.37–7.31 (m, 2H, 3′′-H), 7.14–7.10 (m, 1H, 4′′-H), 6.92 (t, *J* = 55.5 Hz, 1H, CF_2_**H**), 6.92–6.88 (m, 1H, 5′-H), 6.50 (d, *J* = 15.5 Hz, 1H, 2-H), 3.89 (s, 3H, C**H_3_**O); ^13^C-NMR (CDCl_3_) δ: 164.0 (1-C), 158.5 (t, *J*_CF_ = 5.6 Hz, 4′-C), 141.2 (3-C), 138.1 (1′′-C), 132.5 (6′-C), 129.1 (3′′-C), 127.4 (1′-C), 125.2 (t, *J*_CF_ = 5.7 Hz, 2′-C), 124.4 (4′′-C), 123.2 (t, *J*_CF_ = 22.2 Hz, 3′-C), 120.0 (2′′-C), 119.7 (2-C), 111.1 (5′-C), 111.2 (t, *J*_CF_ = 236.4 Hz, **C**F_2_H), 55.9 (**C**H_3_O). EIMS *m*/*z* (%): 303 (25, M^+^), 211 (100), 183 (17), 132 (15), 93 (19). Analysis for C_17_H_15_F_2_NO_2_: Calcd C, 67.32; H, 4.98; N, 4.62%. Found: C, 67.31; H, 5.00; N, 4.37%.

#### 3.2.9. *N*-(2-Phenylethyl)-3-[3-(difluoromethyl)-4-methoxyphenyl]-(*E*)-propenamide (**11g**) 

Compound **11g** was prepared from acid **9** (30.0 mg, 0.145 mmol), Deoxofluor^®^ 50% in toluene (0.107 mL, 0.435 mmol), 2-phenylethylamine (0.055 mL, 0.435 mmol), and triethylamine (0.061 mL, 0.435 mmol) following the procedure used for **11b**. Compound **11g** was obtained as a crystalline white solid (0.029 g, 60%); m.p. 104–105 °C; ^1^H-NMR (CDCl_3_) δ: 7.72–7.71 (m, 1H, 2′-H), 7.57 (d, *J* = 15.6 Hz, 1H, 3-H), 7.52–7.49 (m, 1H, 6′-H), 7.35–7.30 (m, 2H, 3′′′-H), 7.26–7.21 (m, 3H, 2′′′-H and 4′′′-H), 6.91 (t, *J* = 55.5 Hz, 1H, CF_2_**H**), 6.92–6.88 (m, 1H, 5′-H), 6.26 (d, *J* = 15.5 Hz, 1H, 2-H), 5.74 (t, *J* = 5.3 Hz, 1H, N**H**), 3.88 (s, 3H, C**H_3_**O), 3.66 (td, *J* = 6.9, 5.4 Hz, 2H, 1′′-H), 2.89 (t, *J* = 6.9 Hz, 2H, 2′′-H); ^13^C-NMR (CDCl_3_) δ: 166.0 (1-C), 158.3 (t, *J*_CF_ = 5.6 Hz, 4′-C), 139.9 (3-C), 139.0 (1′′′-C), 132.4 (6′-C), 128.9 (2′′′-C), 128.8 (3′′′-C), 127.7 (1′-C), 126.7 (4′′′-C), 125.1 (t, *J*_CF_ = 5.9 Hz, 2′-C), 123.2 (t, *J*_CF_ = 22.2 Hz, 3′-C), 119.6 (2-C), 111.3 (t, *J*_CF_ = 236.3 Hz, **C**F_2_H), 111.3 (5′-C), 56.0 (**C**H_3_O), 40.9 (1′′-C), 35.8 (2′′-C). EIMS *m*/*z* (%): 331 (34, M^+^), 226 (32), 211 (100), 183 (19), 132 (16), 104 (17), 91 (47). Analysis for C_19_H_19_F_2_NO_2_: Calcd C, 68.87; H, 5.78; N, 4.23%. Found: C, 68.86; H, 5.83; N, 4.10%.

#### 3.2.10. *N*-Methoxy-*N*-methyl-3-[3-(difluoromethyl)-4-methoxyphenyl]-(*E*)-propenamide (**11h**)

Compound **11h** was prepared from acid **9** (30.0 mg, 0.145 mmol), Deoxofluor^®^ 50% in toluene (0.107 mL, 0.435 mmol), *N*,*O*-dimethylhydroxylamine hydrochloride (0.043 g, 0.435 mmol), and triethylamine (0.082 mL, 0.580 mmol) following the procedure used for **11b**. Compound **11h** was obtained as a crystalline white solid (0.026 g, 66%); m.p. 101 °C; ^1^H-NMR (CDCl_3_) δ: 7.83–7.80 (m, 1H, 2′-H), 7.69 (d, *J* = 15.8 Hz, 1H, 3-H), 7.62–7.58 (m, 1H, 6′-H), 6.96 (d, *J* = 15.8 Hz, 1H, 2-H), 6.96–6.93 (m, 1H, 5′-H), 6.94 (t, *J* = 55.5 Hz, 1H, CF_2_**H**), 3.91 (s, 3H, C**H_3_**OAr), 3.78 (s, 3H, N(CH_3_)OC**H_3_**), 3.31 (s, 3H, N(C**H_3_**)OCH_3_); ^13^C-NMR (CDCl_3_) δ: 167.1 (1-C), 158.5 (t, *J*_CF_ = 5.7 Hz, 4′-C), 142.4 (3-C), 132.7 (6′-C), 128.1 (1′-C), 125.5 (t, *J*_CF_ = 5.9 Hz, 2′-C), 123.3 (t, *J*_CF_ = 22.1 Hz, 3′-C), 114.8 (2-C), 111.4 (t, *J*_CF_ = 236.3 Hz, **C**F_2_H), 111.3 (5′-C), 62.1 (N(CH_3_)O**C**H_3_), 56.0 (**C**H_3_OAr), 32.7 (N(**C**H_3_)OCH_3_). EIMS *m*/*z* (%): 271 (3.4, M^+^), 211 (100), 183 (18), 132 (12). Analysis for C_13_H_15_F_2_NO_3_: Calcd C, 57.56; H, 5.57; N, 5.16%. Found: C, 57.84; H, 5.65; N, 5.24%.

#### 3.2.11. *N*,*N*-Bis(2-methoxyethyl)-3-[3-(difluoromethyl)-4-methoxyphenyl]-(*E*)-propenamide (**11i**)

A solution of Deoxofluor^®^ 50% in toluene (0.107 mL, 0.435 mmol) was added dropwise to a suspension of 0.030 g of 3-formyl-4-methoxycinnamic acid (**9**, 0.030 g, 0.145 mmol) in 0.4 mL of dry dichloromethane under an argon atmosphere. The reaction mixture was stirred for 45 min at room temperature. Triethylamine (0.061 mL, 0.435 mmol) was added and the mixture was subsequently stirred for 1 h at room temperature. The solution was diluted with 10 mL of dichloromethane, washed twice with 1M HCl and once with water, dried with anhydrous sodium sulfate, and the solvent evaporated. The resulting solid was purified by flash column chromatography eluting with mixtures of ethyl acetate–hexane of increasing polarity to give **11i** as a crystalline white solid (0.042 g, 84%); m.p. 60–61 °C; ^1^H-NMR (CDCl_3_) δ: 7.76–7.71 (m, 1H, 2′-H), 7.64 (d, *J* = 15.4 Hz, 1H, 3-H), 7.58–7.52 (m, 1H, 6′-H), 6.93 (t, *J* = 55.5 Hz, 1H, CF_2_**H**), 6.95–6.91 (m, 1H, 5′-H), 6.89 (d, *J* = 15.4 Hz, 1H, 2-H), 3.90 (s, 3H, C**H_3_**OAr), 3.72 (t, *J* = 5.8 Hz, 2H, NC**H_2_**), 3.68 (t, *J* = 5.3 Hz, 2H, NC**H_2_**), 3.59 (t, *J* = 5.4 Hz, 2H, CH_3_OC**H_2_**), 3.56 (t, *J* = 5.8 Hz, 2H, CH_3_OC**H_2_**), 3.35 (s, 3H, C**H_3_**OCH_2_), 3.34 (s, 3H, C**H_3_**OCH_2_); ^13^C-NMR (CDCl_3_) δ: 167.0 (1-C), 158.3 (t, *J*_CF_ = 5.60 Hz, 4′-C), 141.3 (3-C), 132.3 (t, *J*_CF_ = 2.05 Hz, 6′-C), 128.4 (1′-C), 125.3 (t, *J*_CF_ = 5.8 Hz, 2′-C), 123.2 (t, *J*_CF_ = 22.1 Hz, 3′-C), 117.0 (2-C), 111.4 (t, *J*_CF_ = 236.2 Hz, **C**F_2_H), 111.3 (5′-C), 71.4 (×2, CH_3_O**C**H_2_), 59.3 and 59.0 (**C**H_3_OCH_2_), 56.0 (**C**H_3_OAr), 49.2 and 47.5 (N**C**H_2_). EIMS *m/z* (%): 344 (5.4, M + 1), 343 (3.2, M^+^), 211 (100), 183 (9), 132 (3). Analysis for C_17_H_23_F_2_NO_4_: Calcd C, 59.46; H, 6.75; N, 4.08%. Found: C, 59.08; H, 6.55; N, 4.10%.

#### 3.2.12. (*E*)-3-[3-(Difluoromethyl)-4-methoxyphenyl]-1-(morpholin-4-yl)propenone (**11j**)

Compound **11j** was prepared from acid **9** (30.0 mg, 0.145 mmol), Deoxofluor^®^ 50% in toluene (0.107 mL, 0.435 mmol), morpholine (0.0376 mL, 0.435 mmol), and triethylamine (0.061 mL, 0.435 mmol) following the procedure used for **11b**. Compound **11j** was obtained as a crystalline white solid (0.024 g, 56%); m.p. 120–121 °C; ^1^H-NMR (CDCl_3_) δ: 7.82–7.75 (m, 1H, 2′-H), 7.68 (d, *J* = 15.4 Hz, 1H, 3-H), 7.56–7.54 (m, 1H, 6′-H), 6.95 (t, *J* = 55.5 Hz, 1H, CF_2_**H**), 6.95–6.93 (m, 1H, 5′-H), 6.78 (d, *J* = 15.4 Hz, 1H, 2-H), 3.91 (s, 3H, C**H_3_**O), 3.79–3.66 (m, 8H, 2′′-H, 3′′-H, 5′′-H and 6′′-H); ^13^C-NMR (CDCl_3_) δ: 165.7 (1-C), 158.4 (t, *J*_CF_ = 5.7 Hz, 4′-C), 142.2 (3-C), 132.7 (6′-C), 128.0 (1′-C), 124.9 (t, *J*_CF_ = 5.8 Hz, 2′-C), 123.3 (t, *J*_CF_ = 22.2 Hz, 3′-C), 115.4 (2-C), 111.3 (t, *J*_CF_ = 237.0 Hz, **C**F_2_H), 111.3 (5′-C), 67.0 (2′′-C and 6′′-C), 56.0 (**C**H_3_O), 46.4 and 42.6 (br s, 3′′-C and 5′′-C). EIMS *m/z* (%): 297 (53, M^+^), 211 (100), 183 (22), 132 (15), 86 (12). Analysis for C_15_H_17_F_2_NO_3_: Calcd C, 60.60; H, 5.76; N, 4.71%. Found: C, 61.02; H, 5.74; N, 4.42%.

#### 3.2.13. *N*-(2,5-Bis(trifluoromethyl)phenyl)-3-[3-(difluoromethyl)-4-methoxyphenyl]-(*E*)-propenamide (**11k**)

A solution of Deoxofluor^®^ 50% in toluene (0.107 mL, 0.435 mmol) was added dropwise to a suspension of 3-formyl-4-methoxycinnamic acid (**9**, 0.030 g, 0.145 mmol) in 0.4 mL of dry dichloromethane under an argon atmosphere. The reaction mixture was stirred for 45 min at room temperature, diluted with 2 mL of dry dichloromethane, and percolated through 2.5 g of silica gel under an argon atmosphere. The silica gel bed was rinsed with 8 mL of dry dichloromethane and the resulting solution was dried under a nitrogen flow to give a white solid. Anhydrous THF (1.0 mL), 2,5-bis(trifluoromethyl)aniline (0.068 mL, 0.435 mmol) and 2.0 M lithium diisopropylamide in THF-heptane-ethylbenzene (0.145 mL, 0.29 mmol) were added and the mixture was stirred for 2 h at 25 °C under an argon atmosphere. The solution was diluted with 10 mL of dichloromethane, quenched with ice-water, washed twice with 1M HCl and once with water, dried with anhydrous sodium sulfate, and the solvent evaporated. The resulting oil was purified by flash column chromatography eluting with mixtures hexane–ethyl acetate of increasing polarity to give **11k** as a white solid (0.030 g, 47%); m.p. 168–169 °C; ^1^H-NMR (CDCl_3_) δ: 8.83 (bs, 1H, 6′′-H), 7.85–7.82 (m, 1H, 2′-H), 7.78 (d, *J* = 15.5 Hz, 1H, 3-H), 7.76 (d, *J* = 8.3 Hz, 1H, 3′′-H), 7.64 (s, 1H, N**H**), 7.66–7.59 (m, 1H, 6′-H), 7.49 (d, *J* = 8.2 Hz, 1H, 4′′-H), 7.00–6.96 (m, 1H, 5′-H), 6.96 (t, *J* = 55.41 Hz, 1H, CF_2_**H**), 6.47 (d, *J* = 15.4 Hz, 1H, 2-H), 3.93 (s, 3H, C**H_3_**O); ^13^C-NMR (CDCl_3_) δ: 164.1 (1-C), 159.1 (t, *J*_CF_ = 5.57 Hz, 4′-C), 143.3 (3-C), 136.6 (1′′-C), 135.2 (q, *J*_CF_ = 33.4 Hz, 5′′-C), 133.0 (6′-C), 127.0 (q, *J*_CF_ = 5.40 Hz, 3′′-C), 127.0 (1′-C), 125.8 (t, *J*_CF_ = 5.78 Hz, 2′-C), 123.6 (q, *J*_CF_ = 273.1 Hz, 2′′-**C**F_3_), 123.6 (t, *J*_CF_ = 22.2 Hz, 3′-C), 123.3 (q, *J*_CF_ = 273.2 Hz, 5′′-**C**F_3_), 122.3 (q, *J*_CF_ = 30.1 Hz, 2”-C), 121.0 (q, *J*_CF_ = 3.8 Hz, 6”-C), 120.9 (q, *J*_CF_ = 3.7 Hz, 4”-C), 118.5 (2-C), 111.5 (5′-C), 111.2 (t, *J*_CF_ = 236.6 Hz, **C**F_2_H), 56.1 (**C**H_3_O). EIMS *m*/*z* (%): 439 (5, M^+^), 420 (2), 212 (12), 211 (100), 183 (15), 132 (12). Analysis for C_19_H_13_F_8_NO_2_·0.5H_2_O: Calcd C, 50.90; H, 3.15; N, 3.12%. Found: C, 51.20; H, 3.10; N, 3.50%. 

#### 3.2.14. *N*,*N*’-Bis [3-[3-(difluoromethyl)-4-methoxyphenyl]-(*E*)-propenoyl]-1,3-propanediamine (**11l**)

Compound **11l** was prepared from acid **9** (30.0 mg, 0.145 mmol), Deoxofluor^®^ 50% in toluene (0.107 mL, 0.435 mmol), 1,3-diaminopropane (0.0055 mL, 0.065 mmol), and triethylamine (0.061 mL, 0.435 mmol) following the procedure used for **11b**. Compound **11l** was obtained as a white solid (0.019 g, 59%); m.p. 174–175 °C; ^1^H-NMR (CDCl_3_-CD_3_OD 9:1) δ: 7.74 (m, 2H, 2”-H), 7.56 (d, *J* = 15.8 Hz, 2H, 3′-H), 7.57–7.53 (m, 2H, 6”-H), 6.94–6.90 (m, 2H, 5”-H), 6.92 (t, *J* = 55.5 Hz, 2H, CF_2_**H**), 6.43 (d, *J* = 15.7 Hz, 2H, 2′-H), 3.90 (s, 6H, C**H_3_**O), 3.45–3.35 (m, 4H, 1-H and 3-H), 1.80–1.74 (m, 2H, 2-H); ^13^C-NMR (CDCl_3_-CD_3_OD 9:1) δ: 167.2 (1′-C), 158.3 (t, *J*_CF_ = 5.6 Hz, 4”-C), 139.8 (3′-C), 132.1 (6”-C), 127.6 (1”-C), 125.4 (t, *J*_CF_ = 5.8 Hz, 2”-C), 123.1 (t, *J*_CF_ = 22.1 Hz, 3”-C), 119.6 (2′-C), 111.3 (t, *J*_CF_ = 236.2 Hz, **C**F_2_H), 111.3 (5”-C), 55.9 (**C**H_3_O), 36.5 (1-C and 3-C), 29.3 (2-C). HRMS: calcd for C_25_H_27_F_4_N_2_O_4_^+^ (M + H)^+^: 495.1902, found: 495.1893; HRMS/MS (33 eV) from (M + H)^+^ *m*/*z* (%): 268.1155 (4), 211.0570 (100), 183.0614 (13). Analysis for C_25_H_26_F_4_N_2_O_4_: Calcd C, 60.72; H, 5.30; N, 5.67%. Found: C, 60.46; H, 5.41; N, 5.23%.

#### 3.2.15. 1,4-Bis[3-[3-(difluoromethyl)-4-methoxyphenyl]-(*E*)-propenoyl]-piperazine (**11m**)

Compound **11m** was prepared from acid **9** (30.0 mg, 0.145 mmol), Deoxofluor^®^ 50% in toluene (0.107 mL, 0.435 mmol), piperazinium diacetate (0.015 g, 0.073 mmol), and triethylamine (0.102 mL, 0.730 mmol) following the procedure used for **11b**. Compound **11m** was obtained as a white solid (0.017 g, 46%); m.p. 259–261 °C; ^1^H-NMR (CDCl_3_-CD_3_OD 9:1) δ: 7.80 (m, 2H, 2′-H), 7.66 (d, *J* = 15.3 Hz, 2H, 3-H), 7.65–7.61 (m, 2H, 6′-H), 7.03–6.99 (m, 2H, 5′-H), 6.96 (t, *J* = 55.5 Hz, 2H, CF_2_**H**), 6.88 (d, *J* = 15.4 Hz, 2H, 2-H), 3.93 (s, 6H, C**H_3_**O), 3.86–3.76 (m, 8H, N(C**H_2_**C**H_2_**)_2_N); ^13^C-NMR (CDCl_3_-CD_3_OD 9:1) δ: 166.3 (1-C), 158.5 (t, *J*_CF_ = 5.6 Hz, 4′-C), 142.8 (3-C), 132.3 (br s, 6′-C), 127.4 (1′-C), 125.2 (br s, 2′-C), 123.1 (t, *J*_CF_ = 22.2 Hz, 3′-C), 114.8 (2-C), 111.2 (5′-C), 111.1 (t, *J*_CF_ = 236.1 Hz, **C**F_2_H), 55.7 (**C**H_3_O), 45.4 and 42.2 (br s, N(**C**H_2_**C**H_2_)_2_N). HRMS calcd for C_26_H_27_F_4_N_2_O_4_^+^ (M + H)^+^: 507.1902, found: 507.1892; HRMS/MS (33 eV) from (M + H)^+^ *m*/*z* (%): 211.0558 (100), 183.0610 (31), 160.0515 (4). Analysis for C_26_H_26_F_4_N_2_O_4_·0.5H_2_O: Calcd C, 60.58; H, 5.28; N, 5.43%. Found: C, 60.13; H, 5.15; N, 5.19%.

#### 3.2.16. *N*-(1-Methylethyl)-3-(4-methoxyphenyl)-(*E*)-propenamide (**13a**)

To a solution of 4-methoxycinnamic acid (**12a**, 50 mg, 0.28 mmol) in dry DMF (0.6 mL), triethylamine (0.059 mL, 0.42 mmol), isopropylamine (0.048 mL, 0.56 mmol) and a solution of (benzotriazol-1-yloxy)tris(dimethylamino)phosphonium hexafluorophosphate (0.186 g, 0.42 mmol) in dry dichloromethane (0.6 mL) were added at 0 °C. The reaction mixture was stirred at 0 °C for 30 min and then at 25 °C for 2 h. Water (15 mL) was added and the mixture was extracted with dichloromethane (30 mL). The extract was washed with 1M HCl and water, dried over anhydrous sodium sulfate, and the solvent evaporated. The residue was recrystallized from hexane to give **13a** as a crystalline white solid (0.052 g, 85%); m.p. 131 °C; ^1^H-NMR (CDCl_3_) δ: 7.57 (d, *J* = 15.6 Hz, 1H, 3-H), 7.44 (d, *J* = 8.6 Hz, 2H, 2′-H and 6′-H), 6.88 (d, *J* = 8.7 Hz, 2H, 3′-H and 5′-H), 6.24 (d, *J* = 15.6 Hz, 1H, 2-H), 5.49 (d, *J* = 8.2 Hz, 1 H, N**H**), 4.28–4.17 (m, 1H, 1”-H), 3.82 (s, 3 H, C**H_3_**O), 1.22 (d, *J* = 6.6 Hz, 6 H, 2”-H); ^13^C-NMR (CDCl_3_) δ: 165.5 (1-C), 160.9 (4′-C), 140.5 (3-C), 129.4 (2′-C and 6′-C), 127.8 (1′-C), 118.8 (2-C), 114.3 (3′-C and 5′-C), 55.5 (**C**H_3_O), 41.7 (1”-C), 23.0 (2”-C). EIMS *m*/*z* (%): 220 (18, M + 1), 219 (51, M^+^), 161 (100), 134 (28), 133 (40), 118 (14). Analysis for C_13_H_17_NO_2_: C, 71.21; H, 7.81; N, 6.39%. Found: C, 70.93; H, 7.82; N, 6.36%.

#### 3.2.17. *N*-(1-Methylethyl)-3-(4-methoxy-3-methylphenyl)-(*E*)-propenamide (**13b**)

Compound **13b** was prepared from acid **12b** (50 mg, 0.26 mmol), triethylamine (0.054 mL, 0.39 mmol), isopropylamine (0.045 mL, 0.52 mmol), and (benzotriazol-1-yloxy)-tris(dimethylamino)-phosphonium hexafluorophosphate (0.173 g, 0.39 mmol) following the procedure used for **13a**. The reaction product was purified by flash column chromatography eluting with mixtures of hexane–ethyl acetate of increasing polarity to give **13c** as a pale yellow solid (0.049 g, 80%); m.p. 124–125 °C; ^1^H-NMR (CDCl_3_) δ: 7.54 (d, *J* = 15.5 Hz, 1H, 3-H), 7.33–7.29 (m, 2H, 2′-H and 6′-H), 6.80 (d, *J* = 9.0 Hz, 1H, 5′-H), 6.22 (d, *J* = 15.5 Hz, 1H, 2-H), 5.38 (d, *J* = 6.6 Hz, 1H, N**H**), 4.30–4.16 (m, 1H, 1”-H), 3.85 (s, 3H, C**H_3_**O), 2.21 (s, 3H, C**H_3_**Ar), 1.22 (d, *J* = 6.5 Hz, 6H, 2”-H); ^13^C-NMR (CDCl_3_) δ: 165.6 (1-C), 159.2 (4′-C), 140.8 (3-C), 129.8 (2′-C), 127.5 (6′-C), 127.2 (1′-C and 3′-C), 118.4 (2-C), 110.0 (5′-C), 55.5 (**C**H_3_O), 41.6 (1”-C), 23.1 (2”-C), 16.4 (**C**H_3_Ar). EIMS *m*/*z* (%): 234 (13, M + 1), 233 (36, M^+^), 175 (100), 148 (32), 147 (26), 115 (17). Analysis for C_14_H_19_NO_2_·0.25H_2_O: Calcd: C, 70.71; H, 8.26; N, 5.89%. Found: C, 70.77; H, 8.15; N, 5.86%.

#### 3.2.18. *N*-(1-Methylethyl)-3-(3-hydroxy-4-methoxyphenyl)-(*E*)-propenamide (**13c**)

Compound **13c** was prepared from isoferulic acid (**8**, 54 mg, 0.28 mmol), triethylamine (0.059 mL, 0.42 mmol), isopropylamine (0.048 mL, 0.56 mmol), and (benzotriazol-1-yloxy)-tris(dimethylamino)-phosphonium hexafluorophosphate (0.186 g, 0.42 mmol) following the procedure used for **13a**. The reaction product was purified by flash column chromatography eluting with mixtures of hexane–ethyl acetate of increasing polarity to give **13b** as a white solid (0.048 g, 73%); m.p. 164–165 °C; ^1^H-NMR (CDCl_3_-CD_3_OD 9:1) δ: 7.46 (d, *J* = 15.6 Hz, 1H, 3-H), 7.08 (d, *J* = 2.1 Hz, 1H, 2′-H), 6.98 (dd, *J* = 2.0, 8.6 Hz, 1H, 6′-H), 6.83 (d, *J* = 8.3 Hz, 1H, 5′-H), 6.25 (d, *J* = 15.6 Hz,1H, 2-H), 4.21–4.12 (m, 1H, 1”-H), 3.90 (s, 3H, C**H_3_**O), 1.21 (d, *J* = 6.6 Hz, 6H, 2”-H); ^13^C-NMR (CDCl_3_-CD_3_OD 9:1) δ: 166.1 (1-C), 148.7 (4′-C), 146.0 (3′-C), 140.6 (3-C), 128.4 (1′-C), 121.4 (6′-C), 118.8 (2-C), 112.9 (2′-C), 111.0 (5′-C), 55.9 (**C**H_3_O), 41.5 (1”-C), 22.6 (2”-C). EIMS *m*/*z* (%): 235 (43, M^+^), 178 (35), 177 (98),150 (27), 145 (28), 134 (27), 117 (43), 89 (69), 58 (100). Analysis for C_13_H_17_NO_3_: Calcd C, 66.36; H, 7.28; N, 5.95%. Found: C, 66.28; H, 7.17; N, 5.69%.

#### 3.2.19. *N*-(1-Methylethyl)-3-[4-(difluoromethyl)-3-methoxyphenyl]-(*E*)-propenamide (**15**)

Methyl-3-[4-formyl-3-methoxyphenyl]-(*E*)-propenoate (**14**, 33 mg, 0.150 mmol) was dissolved in a mixture of methanol (2 mL) and 20% aqueous potassium carbonate solution (1 mL). The reaction mixture was stirred for 4 h at 20 °C and concentrated under reduced pressure to a third of its volume. The solution was acidified with conc. HCl (to pH 1) and the precipitate was filtered and used without further purification. The crude product was treated with Deoxofluor^®^ 50% in toluene (0.107 mL, 0.435 mmol), isopropylamine (0.044 mL, 0.435 mmol) and triethylamine (0.061 mL, 0.435 mmol) following the procedure described for **11b**. Compound **15** was obtained as a crystalline white solid (0.025 g, 62% 2 steps); m.p. 128–130 °C; ^1^H-NMR (CDCl_3_) δ: 7.62 (d, *J* = 15.5 Hz, 1H, 3-H), 7.59–7.56 (m, 1H, 5′-H), 7.22–7.18 (m, 1H, 6′-H), 7.05–7.01 (m, 1H, 2′-H), 6.95 (t, *J* = 55.5 Hz, 1H, CF_2_**H**), 6.41 (d, *J* = 15.5 Hz, 1H, 2-H), 5.48 (d, *J* = 6.5 Hz, 1H N**H**), 4.32–4.20 (m, 1H, 1′′-H), 3.92 (s, 3H, C**H_3_**O), 1.26 (d, *J* = 6.6 Hz, 6H, 2”-H); ^13^C-NMR (CDCl_3_) δ: 164.6 (s, 1-C), 157.6 (t, *J* = 6.0 Hz, 3′-C), 140.0 (s, 3-C), 138.9 (t, *J* = 1.8 Hz, 1′-C), 126.8 (t, *J* = 5.8 Hz, 5′-C), 123.8 (t, *J* = 22.3 Hz, 4′-C), 122.9 (s, 2-C), 120.0 (s, 6′-C), 111.4 (t, *J* = 236.4 Hz, **C**F_2_H), 110.2 (s, 2′-C), 55.9 (s, **C**H_3_O), 41.9 (s, 1”-C), 23.0 (s, 2”-C). EIMS *m*/*z* (%): 269 (55, M^+^), 211 (100), 183 (24), 132 (18), 58 (33). Analysis for C_14_H_17_F_2_NO_2_: Calcd C, 62.42; H, 6.36; N, 5.20%. Found: C, 62.44; H, 6.35; N, 4.99%.

#### 3.2.20. 3-(4-Acetyloxy-3-formylphenyl)-(*E*)-propenoic acid (**17**)

To a solution of 5-iodosalicylaldehyde **16** (2.68 g, 10.8 mmol) in dry acetone (50 mL) was added K_2_CO_3_ (3.0 g, 21.6 mmol) and then acetic anhydride (2.15 mL, 21.6 mmol) was added dropwise to the suspension with vigorous stirring. Stirring was continued at room temperature for 2.5 h and the reaction mixture was percolated through a silica gel pad using acetone as eluent. The percolate was evaporated to dryness and the residue recrystallized from n-hexane to give 5-iodo-2-acetyloxy-benzaldehyde as pale yellow needles (2.9 g, 92%); m.p. 89–90 °C; ^1^H-NMR (CDCl_3_) δ: 10.01 (s, 1H, ArC**H**O), 8.16 (d, *J* = 2.2 Hz, 1H, 2-H), 7.91 (dd, *J* = 2.3, 8.5 Hz, 1H, 6-H), 6.96 (d, *J* = 8.5 Hz, 1H, 5-H), 2.38 (s, 3H, C**H_3_**C(O)Ar); ^13^C-NMR (CDCl_3_) δ: 187.0 (Ar**C**HO), 168.7 (CH_3_**C**(O)Ar), 151.3 (2-C), 143.8 (4-C), 139.5 (6-C), 129.5 (1-C), 125.5 (3-C), 90.2 (5-C), 20.8 (**C**H_3_C(O)Ar).

The 5-iodo-2-acetyloxybenzaldehyde obtained above (0.2 g, 0.69 mmol) was dissolved in 5 mL of acetonitrile and oxygen was removed by bubbling nitrogen through the solution. Triethylamine (0.673 mL, 4.82 mmol) and acrylic acid (0.095 mL, 1.38 mmol) were added dropwise with stirring to the solution, followed by tri-*o*-tolylphosphine (16 mg, 0.069 mmol) and palladium (II) acetate (8.0 mg, 0.047 mmol). The mixture was then heated at 65–70 °C for 4 h, volatiles were removed by distillation, and the residue was dissolved in dichloromethane. The resulting solution was percolated through silica gel eluting with a mixture of hexane–ethyl acetate (6:4). The percolate was evaporated to dryness and purified by flash column chromatography eluting with mixtures of hexane–ethyl acetate of increasing polarity to give compound **17** as a white solid (0.095 g, 54%); m.p. 205–207 °C; ^1^H-NMR (DMSO-d_6_) δ: 12.53 (s, 1H, COO**H**), 10.07 (s, 1H, ArC**H**O), 8.22 (d, *J* = 2.3 Hz, 1H, 2′-H), 8.08 (dd, *J* = 8.6, 2.3 Hz, 1H, 6′-H), 7.67 (d, *J* = 16.0 Hz, 1H, 3-H), 7.36 (d, *J* = 8.5 Hz, 1H, 5′-H), 6.63 (d, *J* =16.1 Hz, 1H, 2-H), 2.35 (s, 3H, C**H_3_**CO); ^13^C-NMR (DMSO-d_6_) δ: 189.9 (Ar**C**HO), 169.1 (CH_3_**C**O), 167.3 (1-C), 151.7 (4′-C), 141.8 (3-C), 134.7 (6′-C), 132.8 (1′-C), 131.2 (2′-C), 128.2 (3′-C), 124.5 (5′-C), 120.9 (2-C), 20.7 (**C**H_3_CO). EIMS *m*/*z* (%): 234 (1, M^+^), 192 (23), 146 (9), 91 (20), 89 (45), 63 (19), 43 (100).

#### 3.2.21. *N*-(1-Methylethyl)-3-[4-acetyloxy-3-(difluoromethyl)phenyl]-(*E*)-propenamide (**18a**)

Compound **18a** was prepared from acid **17** (30.0 mg, 0.128 mmol), Deoxofluor^®^ 50% in toluene (0.095 mL, 0.386 mmol), isopropylamine (0.033 mL, 0.386 mmol), and triethylamine (0.054 mL, 0.386 mmol) following the procedure used for **11b**. Compound **18a** was obtained as a white solid (0.018 g, 47%); m.p. 131–133 °C; ^1^H-NMR (CDCl_3_) δ: 7.76–7.71 (m, 1 H, 2′-H), 7.60 (d, *J* = 15.5 Hz, 1H, 3-H), 7.61–7.55 (m, 1H, 6′-H), 7.23–7.18 (m, 1H, 5′-H), 6.74 (t, *J* = 55.2 Hz, 1H, CF_2_**H**), 6.36 (d, *J* = 15.6 Hz, 1H, 2-H), 5.50 (d, *J* = 7.8 Hz, 1 H, N**H**), 4.28–4.17 (m, 1H, 1”-H), 2.34 (s, 3 H, C**H_3_**CO), 1.23 (d, *J* = 6.6 Hz, 6 H, 2”-H); ^13^C-NMR (CDCl_3_) δ: 168.7 (CH_3_**C**O), 164.5 (1-C), 149.1 (t, *J*_CF_ = 5.2 Hz, 4′-C), 138.9 (3-C), 133.4 (1′-C), 131.2 (br s, 6′-C), 127.0 (t, *J*_CF_ = 22.6 Hz, 3′-C), 125.5 (t, *J*_CF_ = 6.4 Hz, 2′-C), 123.8 (s, 5′-C), 122.6 (s, 2-C), 111.6 (t, *J* = 238.9 Hz, **C**F_2_H), 41.9 (s, 1′′-C), 23.0 (s, 2”-C), 21.0 (**C**H_3_CO). EIMS *m*/*z* (%): 298 (23, M + 1), 297 (18, M^+^), 255 (88), 197 (68), 177 (44), 101 (30), 58 (100), 43 (60). Analysis for C_15_H_17_F_2_NO_3_: Calcd C, 60.60; H, 5.76; N, 4.71%. Found: C, 60.21; H, 5.76; N, 4.64%.

#### 3.2.22. *N*-(1-Methylethyl)-3-[3-(difluoromethyl)-4-hydroxyphenyl]-(*E*)-propenamide (**18b**)

To a solution of **18a** (10.0 mg, 0.034 mmol) in MeOH (1.0 mL) was added conc. H_2_SO_4_ (50 µL) and the mixture stirred at 60 °C for 2 h. The reaction mixture was diluted with water (15 mL) and concentrated under reduced pressure. The milky suspension was extracted with ethyl acetate (30 mL), the organic layer was dried with anhydrous sodium sulfate, and the solvent evaporated. The residue was purified by flash column chromatography eluting with mixtures of hexane–ethyl acetate of increasing polarity to give compound **18b** as a white amorphous solid (0.007 g, 82%); m.p. 153–154 °C; ^1^H-NMR (CDCl_3_-CD_3_OD 9:1) δ: 7.70–7.65 (m, 1H, 2′-H), 7.50 (d, *J* 15.6, 1H, 3-H), 7.41–7.35 (m, 1H, 6′-H), 6.95 (t, 1H, *J* = 55.7 Hz, CF_2_**H**), 6.87–6.82 (m, 1H, 5′-H), 6.29 (d, *J* = 15.6 Hz, 1H, 2-H), 4.23–4.13 (m, 1H, 1”-H), 1.21 (d, *J* = 6.5 Hz, 6H, 2”-H); ^13^C-NMR (CDCl_3_-CD_3_OD 9:1) δ: 165.9 (1-C), 156.6 (t, *J*_CF_ = 5.6 Hz, 4′-C), 140.0 (3-C), 132.0 (6′-C), 126.6 (1′-C), 125.3 (t, *J*_CF_ = 5.6 Hz, 2′-C), 121.5 (t, *J*_CF_ = 22.3 Hz, 3′-C), 118.9 (2-C), 116.1 (5′-C), 111.8 (t, *J*_CF_ = 235.5 Hz, **C**F_2_H), 41.6 (1”-C), 22.7 (2”-C). EIMS *m/z* (%): 255 (13, M^+^), 197 (36), 177 (45), 121 (23), 101 (77), 58 (100), 43 (33). HRMS: calcd for C_13_H_16_F_2_NO_2_^+^ (M + H)^+^: 256.1144, found: 256.1145.

#### 3.2.23. *N*-(1-Methylethyl)-*trans*-2-[3-(difluoromethyl)-4-methoxyphenyl]-cyclopropanecarboxamide (19)

Compound **S2** (see the Supplementary Materials) (0.018 g, 0.066 mmol) was dissolved in diethyl ether (0.1 mL) and water (0.003 mL) and potassium *t*-butoxide (40 mg, 0.036 mmol) were added. The mixture was stirred at room temperature for 3 h. After this, the reaction mixture was diluted with 2M HCl (2 mL) and extracted with dichloromethane, the organic layer was dried with anhydrous sodium sulfate, and the solvent evaporated. The resulting crude product was treated with BOP and isopropylamine following the same procedure used for compound **13a**. The residue was purified by flash column chromatography eluting with mixtures of hexane–ethyl acetate of increasing polarity to give compound **19** as a white solid (0.015 g, 80%); m.p. 166–167 °C; ^1^H-NMR (CDCl_3_) δ: 7.22 (bs, 1H, 2′-H), 7.24–7.19 (m, 1H, 6′-H), 6.91 (t, *J* = 55.7 Hz, 1H, CF_2_**H**), 6.86 (m, 1H, 5′-H), 5.47 (d, *J* = 7.2 Hz, 1H, N**H**), 4.20–4.04 (m, 1H, 1′′-H), 3.84 (s, 3H, C**H_3_**OAr), 2.47 (ddd, *J* = 4.1, 6.3, 9.1 Hz, 1H, 2-H), 1.58 (ddd, *J* = 4.1, 5.2, 9.1 Hz, 1H, 3a-H), 1.49 (ddd, *J* = 4.1, 5.2, 8.2 Hz, 1H, 1-H), 1.17 (d, *J* = 6.6 Hz, 3H, 2a’’-H), 1.19–1.14 (m, 1H, 3b-H), 1.17 (d, *J* = 6.6 Hz, 3H, 2b’’-H); ^13^C-NMR (CDCl_3_) δ: 170.8 (**C**(O)N), 155.9 (t, *J*_CF_ = 6.0 Hz, 4′-C), 133.4 (1′-C), 130.4 (t, *J*_CF_ = 2.1 Hz, 6′-C), 123.3 (t, *J*_CF_ = 5.7 Hz, 2′-C), 122.8 (t, *J*_CF_ = 22.0 Hz, 3′-C), 111.6 (t, *J*_CF_ = 235.6 Hz, **C**F_2_H), 111.2 (5′-C), 55.9 (**C**H_3_OAr), 41.8 (1′′-C), 26.6 (1-C), 24.1 (1-C), 23.1 (2b’’-C), 23.0 (2a’’-C), 15.8 (3-C). EIMS *m*/*z* (%): 284 (39, M + 1), 283 (100, M^+^), 264 (29), 224 (21), 197 (30), 178 (21), 146 (42), 43 (15). Analysis for C_15_H_19_F_2_NO_2_: Calcd C, 63.59; H, 6.76; N, 4.94%. Found: C, 63.29; H, 6.50; N, 4.82%.

#### 3.2.24. *N*-(1-Methylethyl)-3-[3-(difluoromethyl)-4-methoxyphenyl]-propanamide (**20**)

Compound **11b** (0.030 g, 0.111 mmol) was dissolved in ethyl acetate (10 mL), 10% wt. Pd/C (0.003 g) added and the mixture hydrogenated at 3 bar and room temperature for 8 h. The catalyst was filtered and the residue crystallized from n-hexane to give compound **20** as a crystalline white solid (0.028 g, 93%); m.p. 84 °C; ^1^H-NMR (CDCl_3_) δ: 7.38 (m, 1H, 2′-H), 7.30 – 7.23 (m, 1H, 6′-H), 6.92 (t, *J* = 55.7 Hz, 1H, CF_2_**H**), 6.86–6.83 (m, 1H, 5′-H), 5.16 (d, *J* = 6.4 Hz, 1H, N**H**), 4.05 (d heptet, *J* = 6.6, 7.9 Hz, 1H, 1′′-H), 3.84 (s, 3H, C**H_3_**OAr), 2.94 (t, *J* = 7.6 Hz, 2H, 3-H), 2.40 (t, *J* = 7.6 Hz, 2H, 2-H), 1.08 (d, *J* = 6.6 Hz, 6H, 2′′-H); ^13^C-NMR (CDCl_3_) δ: 171.1 (1-C), 155.9 (t, *J*_CF_ = 6.1 Hz, 4′-C), 133.3 (1′-C), 132.1 (6′-C), 126.0 (t, *J*_CF_ = 5.7 Hz, 2′-C), 122.7 (t, *J*_CF_ = 22.2 Hz, 3′-C), 111.7 (t, *J*_CF_ = 235.3 Hz, **C**F_2_H), 111.2 (5′-C), 55.9 (**C**H_3_OAr), 41.5 (1′′-C), 38.9 (2-C), 31.0 (3-C), 22.9 (2′′-C). EIMS *m*/*z* (%): 272 (70, M + 1), 271 (100, M^+^), 252 (21), 184 (46), 171 (46), 100 (19). Analysis for C_14_H_19_F_2_NO_2_: Calcd C, 61.98; H, 7.06; N, 5.16%. Found: C, 62.04; H, 7.02; N, 5.11%.

#### 3.2.25. *N*-(1-Methylethyl)-2-methyl-3-[3-(difluoromethyl)-4-methoxyphenyl]-(*E*)-propenamide (**21**)

Compound **21** was prepared from 2-Methyl-3-(3-formyl-4-methoxyphenyl)-(*E*)-propenoic acid (**S3**, see the Supplementary Materials) (24.0 mg, 0.109 mmol), Deoxofluor^®^ 50% in toluene (0.160 mL, 0.327 mmol), isopropylamine (0.028 mL, 0.327 mmol), and triethylamine (0.046 mL, 0.327 mmol) following the procedure used for **11b**. Compound **21** was obtained as a crystalline white solid (0.020 g, 65%); m.p. 96–97 °C; ^1^H-NMR (CDCl_3_) δ: 7.57–7.53 (m, 1H, 2′-H), 7.47–7.37 (m, 1H, 6′-H), 7.25 (bs, 1H, 3-H), 6.97 – 6.93 (m, 1H, 5′-H), 6.95 (t, *J* = 55.6 Hz, 1H, CF_2_**H**), 5.66 (d, *J* = 7.3 Hz, 1H, N**H**), 4.20 (dhept, *J* = 6.6, 7.7 Hz, 1′′-H), 3.90 (s, 3H, C**H_3_**O), 2.09 (d, *J* = 1.4 Hz, 3H, C=CC**H_3_**), 1.24 (d, *J* = 6.6 Hz, 6H, 2”-H); ^13^C-NMR (CDCl_3_) δ: 168.8 (s, 1-C), 156.8 (t, *J* = 5.9 Hz, 4′-C), 133.2 (t, *J* = 2.1 Hz, 6′-C), 132.4 (s, 3-C), 131.9 (s, 2-C), 128.9 (s, 1′-C), 127.4 (t, *J* = 5.8 Hz, 2′-C), 122.7 (t, *J* = 22.2 Hz, 3′-C), 111.4 (t, *J* = 236.1 Hz, **C**F_2_H), 111.0 (s, 5′-C), 55.9 (s, **C**H_3_O), 41.9 (s, 1”-C), 23.0 (s, 2”-C), 14.4 (s, C=C**C**H_3_). EIMS *m*/*z* (%): 284 (55, M + 1), 283 (100, M^+^), 225 (36), 197 (30), 146 (97), 131 (16), 58 (11). Analysis for C_15_H_19_F_2_NO_2_: Calcd C, 63.59; H, 6.76; N, 4.94%. Found: C, 63.45; H, 6.76; N, 4.94%.

#### 3.2.26. (1-Methylethyl)-3-[3-(difluoromethyl)-4-methoxyphenyl]-(*E*)-propenoate (**22**)

Compound **22** was prepared from acid **9** (30.0 mg, 0.145 mmol), Deoxofluor^®^ 50% in toluene (0.107 mL, 0.435 mmol), isopropanol (0.034 mL, 0.435 mmol), and triethylamine (0.061 mL, 0.435 mmol) following the procedure used for **11b**. Compound **22** was obtained as a white solid (0.033 g, 84%); m.p. 62 °C; ^1^H-NMR (CDCl_3_) δ: 7.77–7.74 (m, 1H, 2′-H), 7.63 (d, *J* = 16.0 Hz, 1H, 3-H), 7.60–7.56 (m, 1H, 6′-H), 6.97–6.93 (m, 1H, 5′-H), 6.92 (t, *J* = 55.5 Hz, 1H, CF_2_**H**), 6.35 (d, *J* = 16.0 Hz, 1H, 2-H), 5.13 (hept, *J* = 6.3 Hz, 1′′-H), 3.91 (s, 3H, C**H_3_**O), 1.31 (d, *J* = 6.3 Hz, 6H, 2”-H); ^13^C-NMR (CDCl_3_) δ: 166.6 (s, 1-C), 158.7 (t, *J* = 5.7 Hz, 4′-C), 143.1 (s, 3-C), 132.1 (t, *J* = 1.9 Hz, 6′-C), 127.5 (s, 1′-C), 126.0 (t, *J* = 5.9 Hz, 2′-C), 123.4 (t, *J* = 22.3 Hz, 3′-C), 117.8 (s, 2-C), 111.4 (s, 5′-C), 111.2 (t, *J* = 236.3 Hz, **C**F_2_H), 67.9 (s, 1”-C), 56.0 (s, **C**H_3_O), 22.1 (s, 2”-C). HRMS: calcd for C_14_H_17_F_2_O_3_^+^ (M + H)^+^ requires *m*/*z* 271.1140, found *m/z* 271.1141.

#### 3.2.27. *N*-(1-Methylethyl)-3-(difluoromethyl)-4-methoxybenzamide (**23**)

*p*-Methoxybenzoic acid (91 mg, 0.60 mmol) was dissolved in anhydrous dichloromethane (0.90 mL) and cooled to −40 °C. Then, dichloromethylmethyl ether (0.49 mL, 0.54 mmol) and a solution of TiCl_4_ (0.144 mL, 1.30 mmol) in anhydrous dichloromethane (0.3 mL) were added dropwise under continuous stirring. The deep red solution was stirred at −40 °C for 1.5 h and then 1M HCl (3 mL) added. The resulting emulsion was stirred at room temperature for 0.5 h and extracted with dichloromethane. The organic layer was dried with anhydrous sodium sulfate and the solvent evaporated. The resulting product was treated with Deoxofluor^®^ 50% in toluene (0.107 mL, 0.435 mmol), isopropyl amine (0.037 mL, 0.435 mmol), and triethylamine (0.061 mL, 0.435 mmol) following the procedure used for **11b**. Compound **23** was obtained as a white solid (0.025 g, 52% for 2 steps); m.p. 99–100 °C; ^1^H-NMR (CDCl_3_) δ: 7.97–7.93 (m, 1H, 6-H), 7.90 – 7.87 (m, 1H, 2-H), 7.00–6.95 (m, 1H, 5-H), 6.94 (t, *J* = 55.4 Hz, 1H, CF_2_**H**), 5.91 (d, *J* = 6.7 Hz, 1H, N**H**), 4.28 (dhept, *J* = 6.6, 7.6 Hz, 1′-H), 3.92 (s, 3H, C**H_3_**O), 1.28 (d, *J* = 6.6 Hz, 6H, 2′-H); ^13^C-NMR (CDCl_3_) δ: 165.5 (s, **C**(O)N), 159.6 (t, *J* = 5.6 Hz, 4-C), 131.8 (s, 6-C), 127.4 (s, 1-C), 124.7 (t, *J* = 5.9 Hz, 2-C), 122.5 (t, *J* = 22.3 Hz, 3-C), 111.3 (t, *J* = 236.4 Hz, **C**F_2_H), 111.0 (s, 5-C), 56.1 (s, **C**H_3_O), 42.1 (s, 1′-C), 23.0 (s, 2′-C). EIMS *m*/*z* (%): 243 (13, M^+^), 185 (100), 157 (4), 127 (4), 109 (2). Analysis for C_12_H_15_F_2_NO_2_: Calcd C, 59.25; H, 6.22; N, 5.76%. Found: C, 59.14; H, 6.23; N, 5.77%.

#### 3.2.28. *N*-(1-Methylethyl)-7-(difluoromethyl)-6-methoxy-3,4-dihydronaphthalene-2-carboxamide (**24**)

6-Methoxy-3,4-dihydronaphthalene-2-carboxylic acid (50 mg, 0.25 mmol) was dissolved in anhydrous dichloromethane (0.38 mL) and cooled to −40 °C. Then, dichloromethylmethyl ether (0.49 mL, 0.54 mmol) and a solution of TiCl_4_ (60 µL, 0.54 mmol) in anhydrous dichloromethane (0.12 mL) were added dropwise under continuous stirring. The deep red solution was stirred at −40 °C for 1.5 h and then 1M HCl (3 mL) was added. The resulting emulsion was stirred at room temperature for 0.5 h and then extracted with dichloromethane. The organic layer was dried with anhydrous sodium sulfate and the solvent evaporated. The resulting product was treated with Deoxofluor^®^ 50% in toluene (0.192 mL, 0.779 mmol), isopropylamine (0.066 mL, 0.779 mmol), and triethylamine (0.109 mL, 0.779 mmol) following the procedure used for **11b**. Compound **24** was obtained as a white solid (0.027 g, 35% for 2 steps); mp 150–152 °C; ^1^H-NMR (CDCl_3_) δ: 7.36 (m, 1H, 8-H), 7.10–7.06 (m, 1H, 1-H), 6.90 (t, *J* = 55.7 Hz, 1H, CF_2_**H**), 6.75 (s, 1H, 5-H), 5.65 (d, *J* = 7.8 Hz, 1H, N**H**), 4.20 (dhept, *J* = 6.5, 7.1 Hz, 1′-H), 3.88 (s, 3H, C**H_3_**O), 2.89 (t, *J* = 8.2 Hz, 2H, 4-H), 2.57 (td, *J* = 1.5, 8.2 Hz, 2H, 3-H), 1.23 (d, *J* = 6.5 Hz, 6H, 2′-H); ^13^C-NMR (CDCl_3_) δ: 167.2 (s, **C**(O)N), 157.6 (t, *J* = 6.0 Hz, 6-C), 141.0 (t, *J* = 1.7 Hz, 10-C), 132.0 (s, 2-C), 129.6 (s, 1-C), 125.8 (s, 9-C), 125.8 (t, *J* = 5.7 Hz, 8-C), 121.1 (t, *J* = 22.4 Hz, 7-C), 111.5 (t, *J* = 235.5 Hz, **C**F_2_H), 110.7 (s, 5-C), 56.0 (s, **C**H_3_O), 41.7 (s, 1′-C), 28.5 (s, 4-C), 23.0 (s, 2′-C), 22.7 (s, 3-C). HRMS: calcd for C_16_H_20_F_2_NO_2_^+^ (M + H)^+^ requires *m*/*z* 296.1456, found *m/z* 296.1457.

#### 3.2.29. *N*-(1-Methylethyl)-7-(difluoromethyl)-6-methoxy-naphthalene-2-carboxamide (**25**)

Compound **24** (10 mg, 0.034 mmol) and 2,3-dichloro-5,6-dicyano-1,4-benzoquinone (11.5 mg, 0.05 mmol) were dissolved in dry toluene (0.8 mL) and the mixture was heated under reflux in an argon atmosphere for 1 h. Then, 5% NaHCO_3_ was added and the mixture extracted with dichloromethane, the organic layer was dried with anhydrous sodium sulfate, and the solvent evaporated. The residue was purified by flash column chromatography eluting with mixtures of hexane–ethyl acetate of increasing polarity to give compound **25** as a white solid (0.0085 g, 86%); m.p. 173–174 °C; ^1^H-NMR (CDCl_3_) δ: 8.22 (bs, 1H, 1-H), 8.11 (m, 1H, 8-H), 7.88 (dd, *J* = 1.8, 8.5 Hz, 1H, 3-H), 7.79 (d, *J* = 8.5 Hz, 1H, 4-H), 7.19 (s, 1H, 5-H), 7.02 (t, *J* = 55.3 Hz, 1H, CF_2_**H**), 6.06 (d, *J* = 7.6 Hz, 1H, N**H**), 4.35 (dhept, *J* = 6.6, 7.7 Hz, 1′-H), 4.00 (s, 3H, C**H_3_**O), 1.31 (d, *J* = 6.6 Hz, 6H, 2′-H); ^13^C-NMR (CDCl_3_) δ: 166.6 (s, **C**(O)N), 156.2 (t, *J* = 4.7 Hz, 6-C), 137.0 (s, 10-C), 131.0 (s, 2-C), 127.9 (t, *J* = 6.9 Hz, 8-C), 127.7 (s, 1-C), 127.1 (s, 4-C), 127.1 (s, 9-C), 125.9 (s, 3-C), 125.0 (t, *J* = 21.6 Hz, 7-C), 111.6 (t, *J* = 236.9 Hz, **C**F_2_H), 105.9 (s, 5-C), 55.9 (s, **C**H_3_O), 42.2 (s, 1′-C), 23.1 (s, 2′-C). HRMS: calcd for C_16_H_18_F_2_NO_2_^+^ (M + H)^+^ requires *m*/*z* 294.1307, found *m*/*z* 294.1300.

### 3.3. Biological Activity

#### 3.3.1. Determination of the Minimum Inhibitory Concentration

The Minimum Inhibitory Concentration (MIC), defined as the lowest antimicrobial agent concentration inhibiting the visible growth of a microorganism following its incubation, was subsequently determined. For the purpose of such determination, the compounds under study were tested against *Acinetobacter guillouiae* ATCC 11171, *Clostridium sporogenes* ATCC 19404, *Enterobacter cloacae* ATCC 35587, *Enterococcus faecalis* ATCC 29212, *Escherichia coli* ATCC 8739, *Klebsiella pneumoniae* ATCC 10273, *Mycobacterium smegmatis* ATCC 607, *Proteus mirabilis* ATCC 10005, *Pseudomonas aeruginosa* ATCC 9027, *Shigella boydii* ATCC 12027, *Shigella flexneri* ATCC 11836, *Staphylococcus aureus* ATCC 29737, *Streptococcus pneumoniae* ATCC 10015, and *Streptococcus pyogenes* ATCC 8133 using Mueller–Hinton (MH) broth.

The MICs of the tested compounds were determined by microdilution with MH broth supplemented with Mg^+2^ and Ca^+2^ cations. A stock solution of the synthesized compounds (1 mg/mL) in DMSO was prepared and graded quantities of the test compounds were incorporated in specified quantity of sterilized liquid medium (MH, supplemented with defibrinated horse blood for *S. pneumoniae* and Tween 80 for *M. smegmatis*) to test different concentrations of the compounds. The test was conducted on a plate on the basis of a 1 × 10^5^ CFU/mL inoculum concentration, with negative, positive, and sterility controls being simultaneously performed. Different concentrations of the compounds under study were tested. Following incubation at 32.5 ± 2.5 °C for a 24 h period (48 h in the case of *C. sporogenes* and 72 h in the case of *M. smegmatis*), the plates were visually read. After incubation, the MICs were visually determined by the presence or absence of visible growth [42,43].

#### 3.3.2. Cytotoxicity Assay

Cytotoxicity of compounds **11b**–**11d**, **11f**–**11g**, **13a**–**13c**, and **18a** was measured using the MTT (3-(4,5-dimethylthiazol-2yl)-2,5-diphenyl tetrazolium bromide; Sigma-Aldrich, USA) method [44], in A549 and HepG2 cells, respectively. Confluent cultures in 96-well plates were exposed to two-fold dilutions of the compounds, with three wells for each dilution, during 48 h of incubation at 37 °C. Then, 10 µL of Maintenance Medium (MM) containing MTT (final concentration 5 mg/mL) was added to each well. After 2 h of incubation, the supernatant was decanted and 200 µL ethanol were added to each well to solubilize the formazan crystals. After vigorous shaking, absorbance was measured in a microplate reader and cytotoxicity was calculated as the cytotoxic concentration 50% (CC_50_), i.e., the compound concentration required to reduce the MTT signal by 50% compared to controls.

## 4. Conclusions

Our results show that the combination of the difluoromethyl group and the *p*-methoxycinamyl scaffold provided both selectivity and increased activity towards *M. smegmatis*, with a high dependency on the characteristics of the substituent on the amide nitrogen. The most active compounds (**11b**, **11d**, and **11g**) had an activity similar to that of the reference antibiotic, exhibited very low cytotoxicity, and were inactive against the other bacteria assayed. This makes them potential leads for the development of narrow spectrum antibiotics against *M. tuberculosis*, where long-term treatments with broad spectrum antibiotics entail an increased risk of damaging the gut microbiota and promoting the appearance of resistant genes [45].

## 5. Patents

The following patents have been granted: Compounds having antibacterial activity process for their preparation and pharmaceutical compositions comprising them, G. Burton, F. J. Durán, M. D. Martinez, E. Zini, V. Mora Muñoz, L. Bertoncello, (CONICET—Laboratorios Richmond), US 9,255,071 B2 (2016); EP 2,802,558 B1 (2016).

## Figures and Tables

**Table 1:** Synthesis of 3′-difluoromethyl-4′-methoxycinnamoyl amides **11a**–**11m**.

Compound	Method ^1^	Amine	R	Yield ^2^
**11a**	A	CH_3_NH_2_·HCl	-NHCH_3_	60%
**11b**	A	*i*-Pr-NH_2_	-NH*i*-Pr	64%
**11c**	A	*i*-Bu-NH_2_	-NH*i*-Bu	66%
**11d**	A	*i*-Pentyl-NH_2_	-NH*i*-Pentyl	58%
**11e**	A	*c*-C_6_H_11_-NH_2_	-NH*c*-C_6_H_11_	76%
**11f**	A	Ph-NH_2_	-NHPh	57%
**11g**	A	Ph(CH_2_)_2_-NH_2_	-NH(CH_2_)_2_Ph	60%
**11h**	A	CH_3_-NH(OCH_3_)·HCl	-N(OCH_3_)CH_3_	66%
**11i**	B	-	-N(CH_2_CH_2_OCH_3_)_2_	84%
**11j**	A	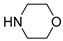	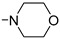	56%
**11k**	C	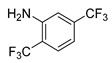	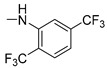	47%
**11l**	D	H_2_N-(CH_2_)_3_-NH_2_	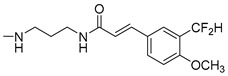	59%
**11m**	D	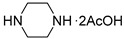	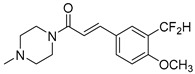	46%

^1^ For Methods A, C and D, the cinnamoyl fluoride (**10**) solution was filtered through a silica gel pad prior to adding the reagents; in Method C, the dichloromethane was then evaporated and replaced with THF. (A) amine (1 eq), Et_3_N-CH_2_Cl_2_; (B) Et_3_N-CH_2_Cl_2_; (C) amine (1 eq), LDA-THF; and (D) diamine (0.5 eq), Et_3_N, CH_2_Cl_2_. ^2^ Isolated yields from **9**.

**Table 2:** Antibacterial activity of compounds **11a**–**11m**, **13a**–**13c**, **15**, **18a**–**18b**, and **19**–**23** ^1^.

	Microorganism (ATCC/Strain)	Reference Antibiotic ^2^ (MIC μg/mL)	Active Compounds ^3^ (MIC μg/mL)
Gram-positive	*Clostridiumsporogenes* (19404)	A (≤ 2.0)	**11e** (64), **11f** (64), **11j** (64), **15** (32)
	*Enterococcusfaecalis* (29212)	A (1.0–4.0)	n.a.
	*Mycobacteriumsmegmatis* (607)	B (≤ 4.0)	**11b** (8), **11c** (32), **11d** (8), **11f** (64), **11g** (8), **13a** (32), **13b** (32), **18a** (64)
	*Staphylococcusaureus* (29737)	A (0.5–2.0)	n.a.
	*Streptococcuspyogenes* (8133) ^4^	C (≤ 0.125)	**11f** (64), **18a** (64)
	*Streptococcuspneumoniae* (10015) ^4^	D (≤ 0.5)	**11l** (64)
Gram-negative	*Acinetobacterguillouiae* (11171) ^4^	E (0.06–8.0)	**11e** (64), **11f** (64), **11i** (64)
	*Enterobactercloacae* (35587) ^4^	B (≤ 2.0)	n.a.
	*Escherichiacoli* (8739)	B (≤ 2.0)	n.a.
	*Klebsiellapneumoniae* (10273) ^4^	B (≤ 2.0)	n.a.
	*Shigellaboydii* (12027) ^4^	F (≤ 4.0)	**13c** (32)
	*Shigellaflexneri* (11836) ^4^	F (≤ 4.0)	**13c** (32)
	*Proteusmirabilis* (10005) ^4^	B (≤ 2.0)	**18a** (64)
	*Pseudomonasaeruginosa* (9027)	B (1.0–4.0)	**11f** (64), **11j** (64), **20** (64)

^1^ Activities were determined by the MH broth microdilution method supplemented with Mg^+2^ and Ca^+2^ cations; ^2^ (A) Vancomycin, (B) Imipenem, (C) Penicillin, (D) Ampicillin sodium salt, (E) Minocycline, (F) Phosphomycin; ^3^ n.a., MICs > 64 μg/mL for all compounds except **11k**, which was tested up to 32 μg/mL due to solubility limitations; ^4^ compound **18b** was not evaluated against these microorganisms.

**Table 3:** Cytotoxicity data of selected compounds on A549 and HepG2 cells.

Compound	A549		HepG2	
	CC_50_^1^ (μg/mL)	SI ^2^	CC_50_ (μg/mL)	SI ^2^
**11b**	>200	>25	199.2 ± 1.1	24.9
**11c**	>200	>6.25	>200	>6.25
**11d**	>200	>25	77.0 ± 13.8	9.63
**11f**	>200	>3.13	>200	>3.13
**11g**	>200	>25	>200	>25
**13a**	>200	>6.25	>200	>6.25
**13b**	>200	>6.25	>200	>6.25
**13c**	>200	>6.25 ^3^	>200	>6.25 ^3^
**18a**	64.4 ± 21.0	1	92.3 ± 2.3	1.44

^1^ CC_50_ is 50% cytotoxicity concentration; ^2^ SI (selectivity index) = CC_50_/MIC*_M. smegmatis_*; ^3^ SI (selectivity index) = CC_50_/MIC*_S. boydii or S. flexneri_*.

## Supplementary Materials

The following are available online. Synthetic procedures and characterization of precursors **12b**, **S1**, **S2**, and **S3**. Table S1: Calculated *LogP* of compounds **11a**–**11m**, **13a**–**11c**, **15**, **18a**, **18b**, and **19**–**25**. Mass spectra for compounds **9**, **11a**–**11m**, **13a**–**13c**, **15**, **17**, **18a**, **18b, 19**–**25**, and **S2**. ^1^H and ^13^C-NMR spectra of compounds **9**, **10**, **11a**–**11m**, **13a**–**13c**, **15**, **17**, **18a**, **18b, 19**–**25**, **S2**, and **S3**.
