# Supplementary material for: Synthesis and Antibacterial Activity of Difluoromethyl Cinnamoyl Amides
Source: Molecules. 2020 Feb 12;25(4):789. doi: 10.3390/molecules25040789 (PMC7070587; doi:10.3390/molecules25040789)
Supplement: Supplementary file 1 [file molecules-25-00789-s001.pdf]

Supporting Information for:

Synthesis and antibacterial activity of difluoromethyl cinnamoyl amides

Mario David Martínez[1,2], Diego Ariel Riva[3,4,5], Cybele Garcia[3,4], Fernando Javier Durán[1,2,*] and Gerardo Burton[1,2,*]

[1] CONICET-Universidad de Buenos Aires, UMYMFOR, Buenos Aires, Argentina;
[2] Universidad de Buenos Aires, Facultad de Ciencias Exactas y Naturales, Departamento de Química Orgánica, Buenos Aires, Argentina
[3] CONICET- Universidad de Buenos Aires, Instituto de Química Biológica (IQUIBICEN), Buenos Aires, Argentina.
[4] Universidad de Buenos Aires, Facultad de Ciencias Exactas y Naturales, Departamento de Química Biológica, Laboratorio de Estrategias Antivirales.
[5] *Current affiliation:* SENASA, Dirección General de Laboratorios y Control Técnico, Dirección del Laboratorio Animal, Coordinación de Virología, Departamento de Cultivo Celular. Ministerio de Agroindustria, Buenos Aires, Argentina.

* Correspondence: fduran@qo.fcen.uba.ar (F.J.D.); burton@qo.fcen.uba.ar (G.B.); Tel.: +541145763385 (G.B.)

Index

S.1. EXPERIMENTAL......3

S.1.1. GENERAL 3

S.1.2. CHEMICAL SYNTHESIS......4

*S.1.2.1. 4-Iodo-1-methoxy-2-methylbenzene (**S1**)*......4

*S.1.2.2 4-Methoxy-3-methyl-(E)-cinnamic acid (**12b**)*......4

*S.1.2.3. N-Methoxy-N-methyl-trans-2-[3-(difluoromethyl)-4-methoxyphenyl]-cyclopropanecarboxamide (**S2**)* 5

*S.1.2.4. 2-Methyl-3-(3-formyl-4-methoxyphenyl)-(E)-propenoic acid (**S3**)*......6

2. CALCULATED *LOGP* VALUES......7

3. ^{1}H AND ^{13}C NMR CHARTS 8

4. MS SPECTRA 39

S.1. Experimental

S.1.1. General

Melting points were taken on a Fisher-Johns apparatus and are uncorrected. ^{1}H and ^{13}C NMR spectra were measured in a Bruker Avance II 500 NMR spectrometer at 500.13 and 125.72 MHz respectively. J values are given in Hz. All assignments were confirmed by a combination of 2D experiments (COSY, HSQC and HMBC). Elemental analysis was performed on an EAI Exeter Analytical, Inc. CE-440 apparatus, North Chelmsford, MA, USA. The electron impact mass spectra (EIMS) were measured in a Shimadzu QP-5000 or in a Thermo DSQ-II mass spectrometer at 70 eV by direct inlet. Exact mass spectra (HRMS) were measured on a Bruker micrOTOF-Q II mass spectrometer with positive electrospray ionization. Analytical thin layer chromatography (TLC) was performed on pre-coated silica gel plates (Merck F254, 0.2 mm thickness); compounds were visualized under 254 nm UV light. Flash column chromatography was performed on silica gel Merck 9385 (0.0040-0.0063 mm). All solvents were distilled and stored over 4 Å molecular sieves before use. Solvents were evaporated at *ca.* 45°C under vacuum in a rotary evaporator. The homogeneity of all compounds was confirmed by TLC. Products obtained as solids or syrups were dried under high vacuum. 5-Iodo-2-methoxybenzaldehyde was obtained by iodination of 2-methoxybenzaldehyde with iodine/silver nitrate in methanol [28].

S.1.2. Chemical synthesis

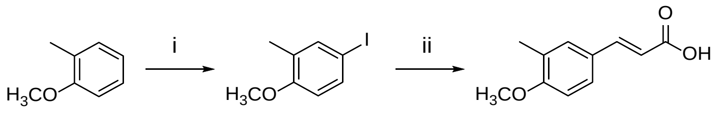

S1 **12b**

Scheme S2. Synthesis of precursors **S1** and **12b**. Reagents and conditions: i) $AgNO_3$, I_2, MeOH; ii) $Pd(AcO)_2$, (*o*-Tol)$_3$P, acrylic acid, HMPA, K_2CO_3, H_2O.

S.1.2.1. 4-Iodo-1-methoxy-2-methylbenzene (**S1**)

To a solution of 2-methylanisole (0.3 mL, 2.42 mmol) in methanol (16.0 mL), silver nitrate (0.41 g, 2.42 mmol) and iodine (0.68 g, 2.66 mmol) were added. The suspension was stirred for 6 h in the dark. The reaction mixture was filtered and the solvent evaporated. The residue was extracted with water and dichloromethane, the organic layer was dried with anhydrous sodium sulfate and the solvent evaporated to give 4-iodo-1-methoxy-2-methylbenzene **S1** as a white solid (0.49 g, 82%). mp 78 ºC (lit. 79-80 ºC) identical (NMR, MS) to that described.

Wan, S.; Wang, S. R.; Lu, W., One-Pot Preparation of Arylalkynes by a Tandem Catalytic Iodination of Arenes and Palladium-Catalyzed Coupling of Iodoarenes with Terminal Alkynes. *The Journal of Organic Chemistry* **2006,** 71, (11), 4349-4352. DOI:10.1021/jo060424x.

S.1.2.2 4-Methoxy-3-methyl-(*E*)-cinnamic acid (**12b**)

Tri(o-tolyl)phosphine (30 mg, 0.1 mmol) and palladium (II) acetate (11.0 mg, 0.05 mmol) were dissolved in hexamethylphosphoramide (0.1 mL). The mixture was stirred for 5 min, then water (0.9 mL), potassium carbonate (0.42 g, 3 mmol), acrylic acid (1.14 mL, 2 mmol) and 4-iodo-1-methoxy-2-methylbenzene S1 (0.25 g, 1 mmol) obtained above were added. The mixture was stirred at 90 °C for 5 h, filtered and the solution was acidified with conc. H2SO4 (to pH 1). The milky suspension was extracted with ethyl acetate (30 mL), the organic layer was dried with anhydrous sodium sulfate and the

solvent evaporated. The resulting solid was recrystallized from isopropanol to give 12b as a pale yellow solid (0.115g, 60 %); mp 200-201 ºC, (lit. 201 ºC) identical (1H NMR) to that described.

Jones, B.; Watkinson, J. G., 817. The alkaline hydrolysis of nuclear-substituted ethyl cinnamates. The cumulative effects of substituents. Journal of the Chemical Society (Resumed) 1958, 4064-4069. DOI:10.1039/JR9580004064.

Jones, K. A.; Weaver, D. F.; Tiedje, K. E. Preparation of dihydrouracils as anti-ictogenic or anti-epileptogenic agents. WO2004009559A2, 2004.

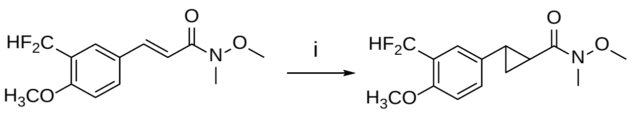

Scheme S3. Synthesis of precursor **S2**. Reagents and conditions: i) $Me_3S(O)I$, NaH, DMSO.

S.1.2.3. *N*-Methoxy-*N*-methyl-*trans*-2-[3-(difluoromethyl)-4-methoxyphenyl]-cyclopropanecarboxamide (**S2**)

The cyclopropane derivative **S2** was prepared from compound **11h** (0.030 g, 0.110 mmol), trimethylsulfoxonium iodide (0.049 g, 0.221 mmol), 60 % dispersion in mineral oil sodium hydride (0.0085 g, 0.221 mmol) following a previously described procedure [49]. The residue was purified by flash column chromatography eluting with mixtures of hexane-ethyl acetate of increasing polarity to give compound **S2** as a white amorphous solid (0.026 g, 83%); ^{1}H NMR ($CDCl_3$) δ: 7.30 (d, *J* = 1.90 Hz, 1H, 2'-H), 7.26 – 7.21 (m, 1H, 6'-H), 6.92 (t, *J* = 55.7 Hz, 1H, CF_2**H**), 6.86 (dt, *J* = 1.3, 8.6 Hz, 1H, 5'-H), 3.85 (s, 3H, C**H3**OAr), 3.71 (s, 3H, N(CH_3)OC**H3**), 3.24 (s, 3H, N(C**H3**)OCH_3), 2.49 (ddd, *J* = 4.2, 6.4, 9.2 Hz, 1H, 2-H), 2.35 (bs, 1H, 1-H), 1.61 (ddd, *J* = 4.3, 5.3, 9.2 Hz, 1H, 3a-H), 1.28 (ddd, *J* = 4.3, 6.4, 8.4 Hz, 1H, 3b-H); ^{13}C NMR ($CDCl_3$) δ: 173.1 (**C**(O)N), 156.0 (t, J_{CF} = 5.8 Hz, 4'-C), 133.1 (1'-C), 130.4 (t, J_{CF} = 1.8 Hz, 6'-C), 123.9 (t, J_{CF} = 5.8 Hz, 2'-

C), 122.8 (t, J_{CF} = 22.0 Hz, 3'-C), 111.6 (t, J_{CF} = 235.7 Hz, **C**F_2H), 111.2 (5'-C), 61.8 (N(CH_3)O**C**H_3), 55.9 (**C**H_3OAr), 32.7 (N(**C**H_3)OCH_3), 25.2 (2-C), 21.5 (1-C), 16.2 (3-C); EIMS *m/z* (%): 285 (37, M^+), 225 (53), 174 (100) , 146 (53) , 131 (30) , 103 (31). Analysis for $C_{14}H_{17}F_2NO_3{\cdot}0.5H_2O$: Calcd C, 57.14; H, 6.16; N, 4.76 %. Found: C, 57.15; H, 5.91; N, 4.66 %.

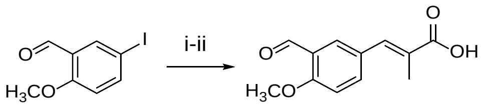

Scheme S4. Synthesis of precursor **S3**. Reagents and conditions: (i) Pd(AcO)$_2$, (*o*-Tol)$_3$P, methyl methacrylate, TEA, MeCN; (ii) K_2CO_3, MeOH-H_2O.

S.1.2.4. 2-Methyl-3-(3-formyl-4-methoxyphenyl)-(*E*)-propenoic acid (**S3**)

The corresponding methacrylic derivative **S3** was prepared from 5-Iodo-2-methoxy-benzaldehyde (0.25 g, 3.82 0.954mmol), triethylamine (3.71 mL, 26.7 mmol), methyl methacrylate (0.686, mL, 7.63 mmol), tri-*o*-tolylphosphine (0.0214 g, 0.095 mmol) and palladium (II) acetate (0.008 g, 0.047 mmol) following the procedure used for the obtention of compound **9**. Compound **S3** was obtained as an amorphous pale yellow solid (0.018 g, 47%); ^{1}H NMR (DMSO-d$_6$) δ: 12.51 (s, 1H, COO**H**), 10.36 (s, 1H, ArC**H**O), 7.82 – 7.77 (m, 2H, 6'-H and 2'-H), 7.57 (m, 1H, 3-H), 7.31 (m, 1H, 5'-H), 3.96 (s, 3H, C**H**$_3$O), 2.03 (d, 3H, J = 1.5 Hz, C**H**$_3$C); ^{13}C NMR (DMSO-d$_6$) 188.9 (Ar**C**HO), 169.3 (1-C), 161.2 (4'-C), 137.8 (6'-C), 136.2 (3-C), 128.9 (2'-C), 128.0 (1'-C), 128.0 (2-C), 123.9 (3'-C), 113.1 (5'-C), 56.2 (**C**H_3O), 13.9 (**C**H_3C).

2. Calculated *LogP* values

The c*LogP* values were obtained employing the Chem3D v16.0 software.

Table S1. Calculated *cLogP* of compounds **11a**-**m**, **13a**-**c**, **17**, **18a**-**b** and **19**-**25**.

Compound	*cLogP*	Compound	*cLogP*
**11a**	1.92	**13a**	2.56
**11b**	2.76	**13b**	3.06
**11c**	3.38	**13c**	1.83
**11d**	3.91	**17**	2.76
**11e**	3.95	**18a**	2.19
**11f**	3.78	**18b**	2.12
**11g**	4.02	**19**	1.62
**11h**	2.72	**20**	1.97
**11i**	2.57	**21**	3.07
**11j**	1.88	**22**	3.42
**11k**	4.70	**23**	2.10
**11l**	4.32	**24**	3.20
**11m**	4.15	**25**	3.19

3. ^{1}H and ^{13}C NMR charts

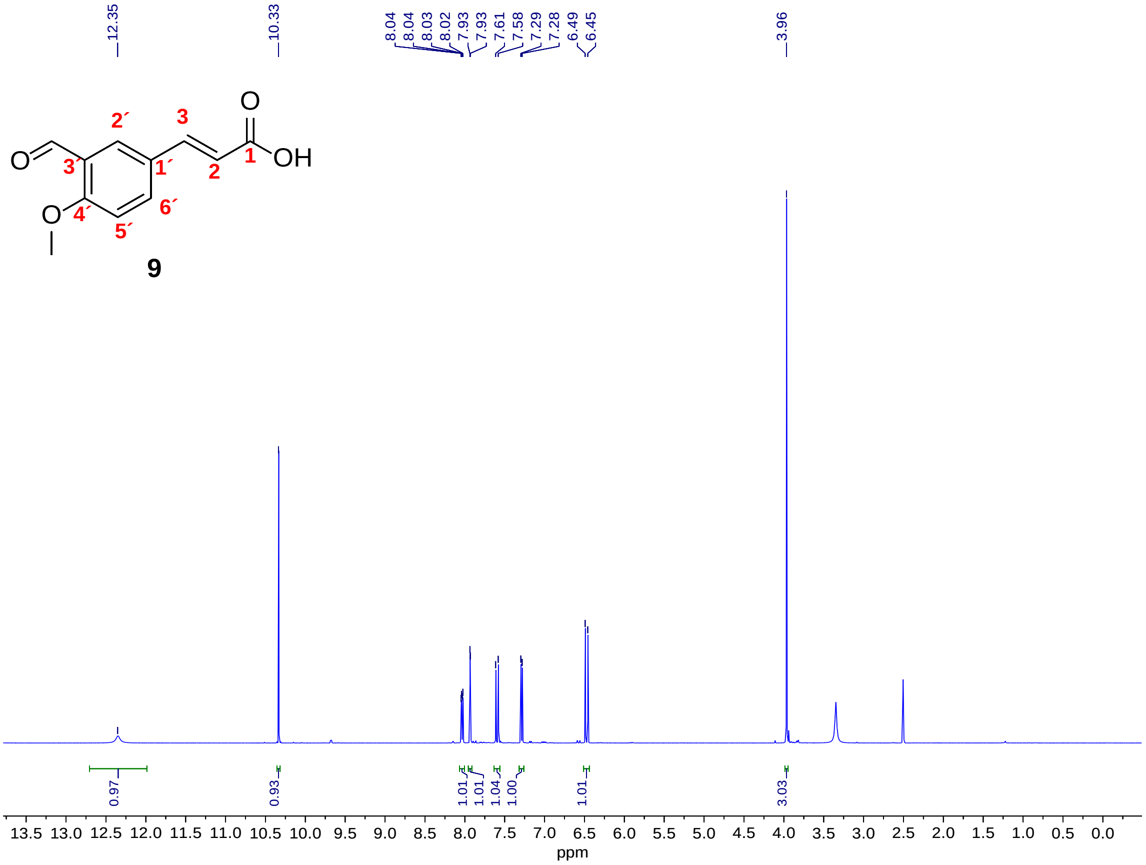

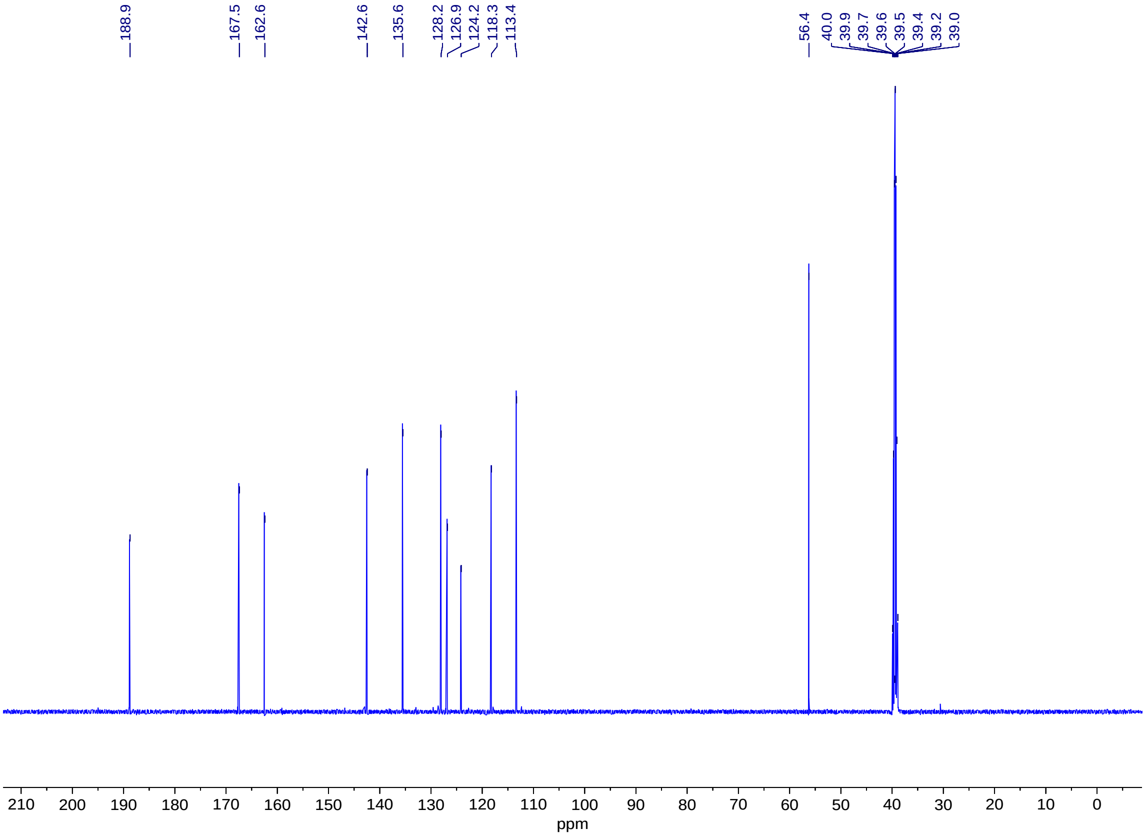

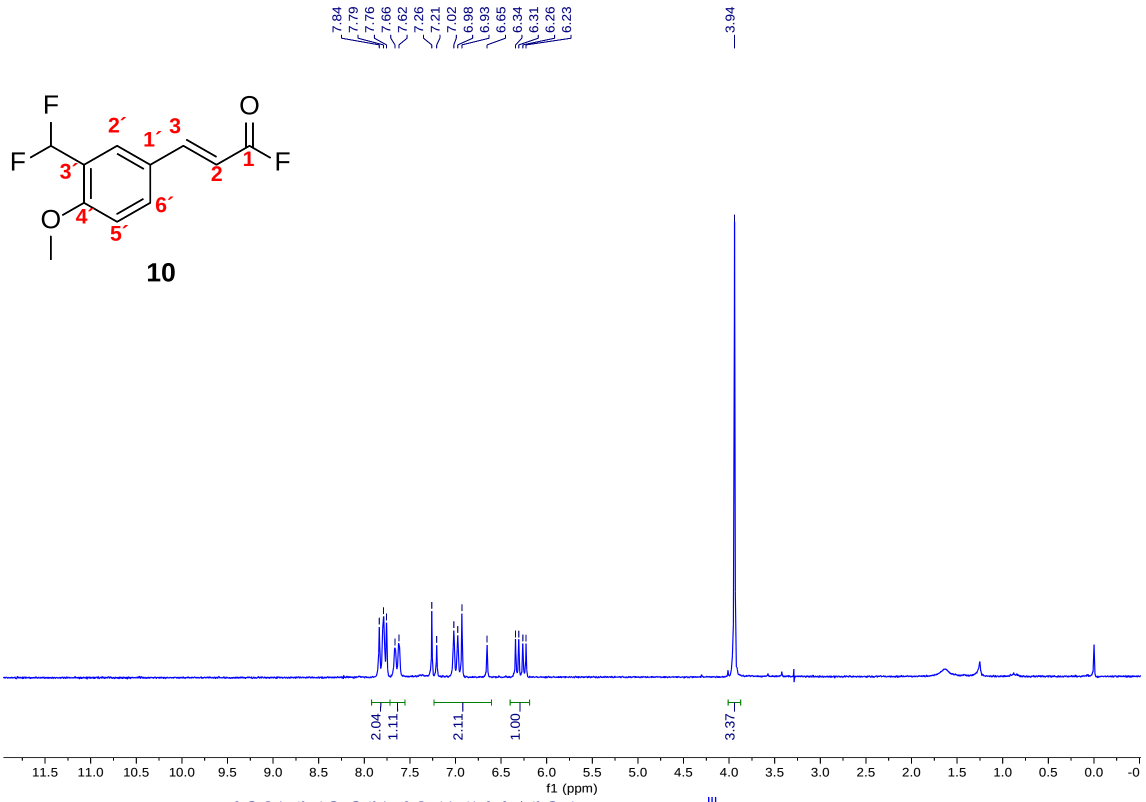
7.84
7.79
7.76
7.66
7.62
7.26
7.21
7.02
6.98
6.93
6.65
6.34
6.31
6.26
6.23
3.94
F
F
2´
1´
3
O
F
3´
2
1
O
4´
6´
5´
10
2.04
1.11
2.11
1.00
3.37
11.5 11.0 10.5 10.0 9.5 9.0 8.5 8.0 7.5 7.0 6.5 6.0 5.5 5.0 4.5 4.0 3.5 3.0 2.5 2.0 1.5 1.0 0.5 0.0 -0
f1 (ppm)

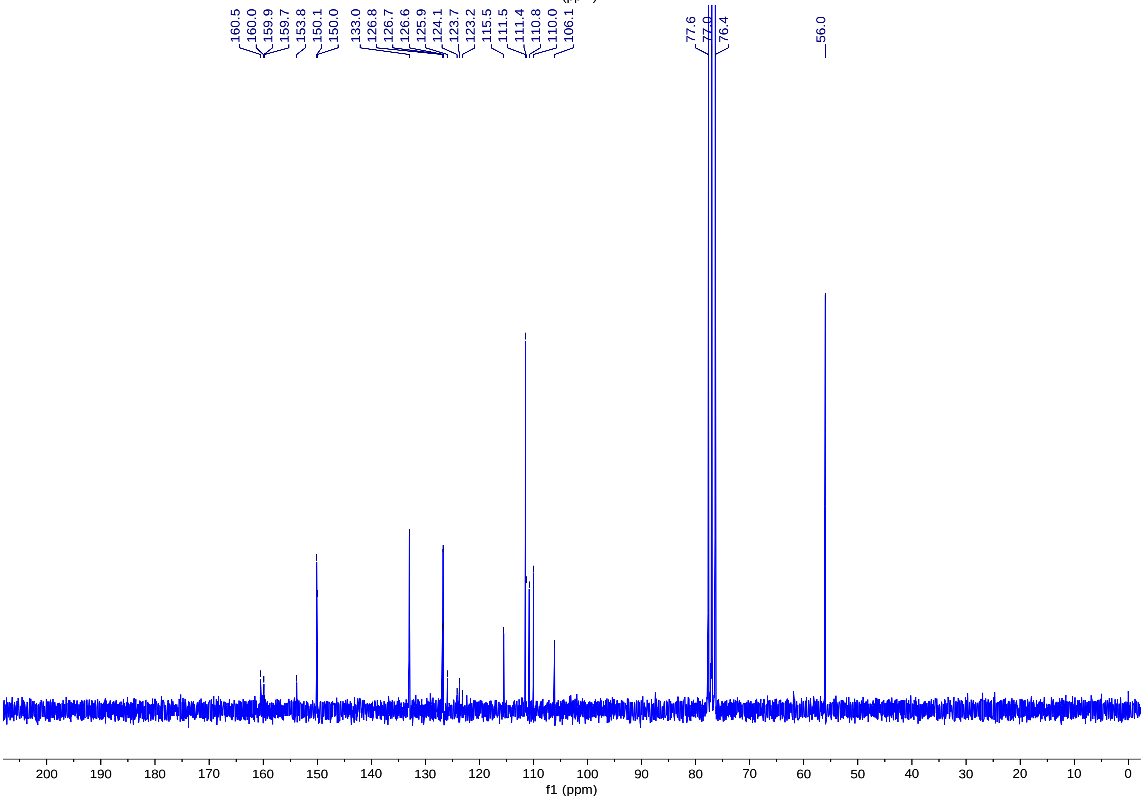
160.5
160.0
159.9
159.7
153.8
150.1
150.0
133.0
126.8
126.7
126.6
125.9
124.1
123.7
123.2
115.5
111.5
111.4
110.8
110.0
106.1
77.6
77.0
76.4
56.0
200 190 180 170 160 150 140 130 120 110 100 90 80 70 60 50 40 30 20 10 0
f1 (ppm)

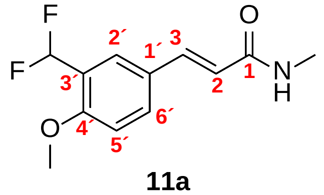
F
F
O
N
H
O
1
2
3
1´
2´
3´
4´
5´
6´

11a

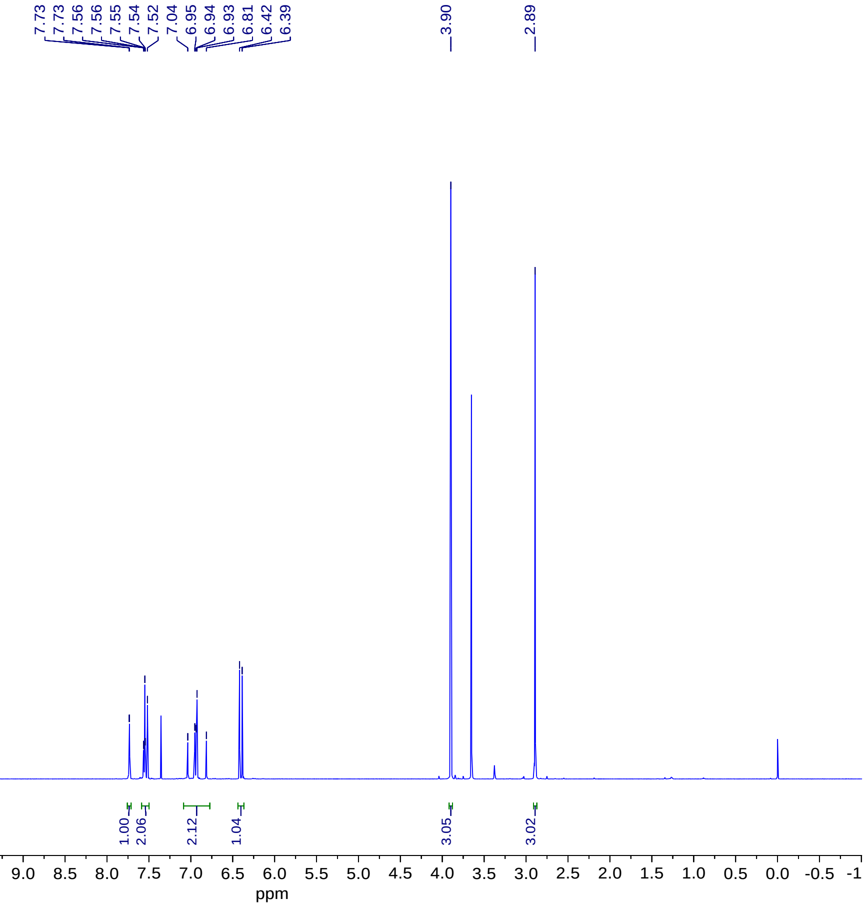
7.73
7.73
7.56
7.56
7.55
7.54
7.52
7.04
6.95
6.94
6.93
6.81
6.42
6.39
3.90
2.89
1.00
2.06
2.12
1.04
3.05
3.02
ppm

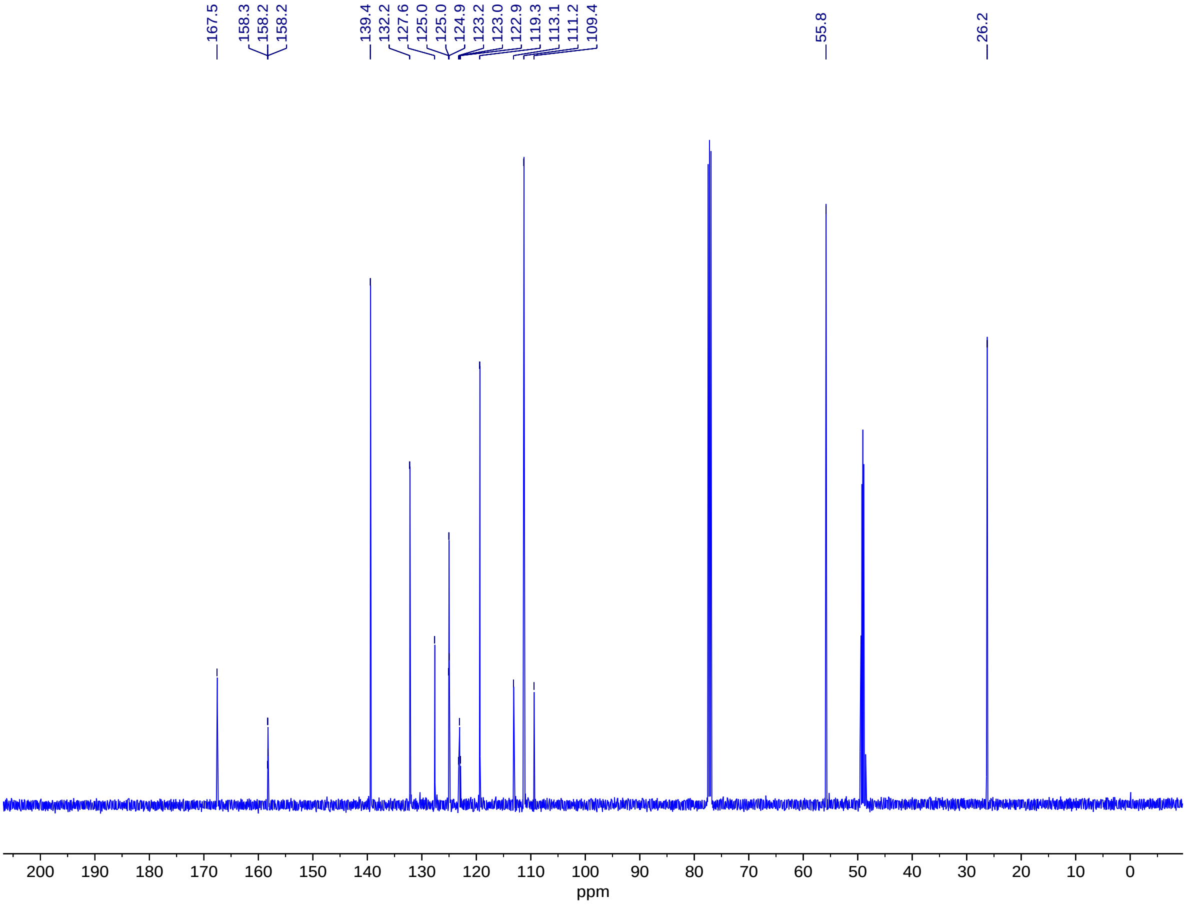
167.5
158.3
158.2
158.2
139.4
132.2
127.6
125.0
125.0
124.9
123.2
123.0
122.9
119.3
113.1
111.2
109.4
55.8
26.2
ppm

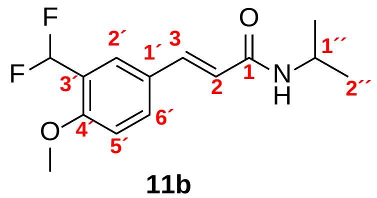
F
F
O
N
H
1
2
3
1´
2´
3´
4´
5´
6´
1´´
2´´

11b

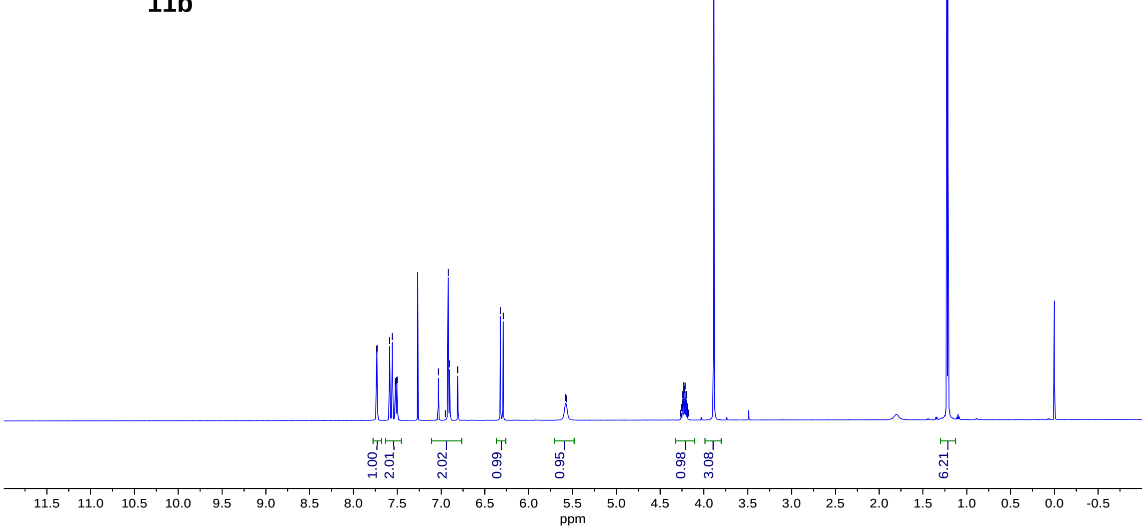
ppm
1.00
2.01
2.02
0.99
0.95
0.98
3.08
6.21

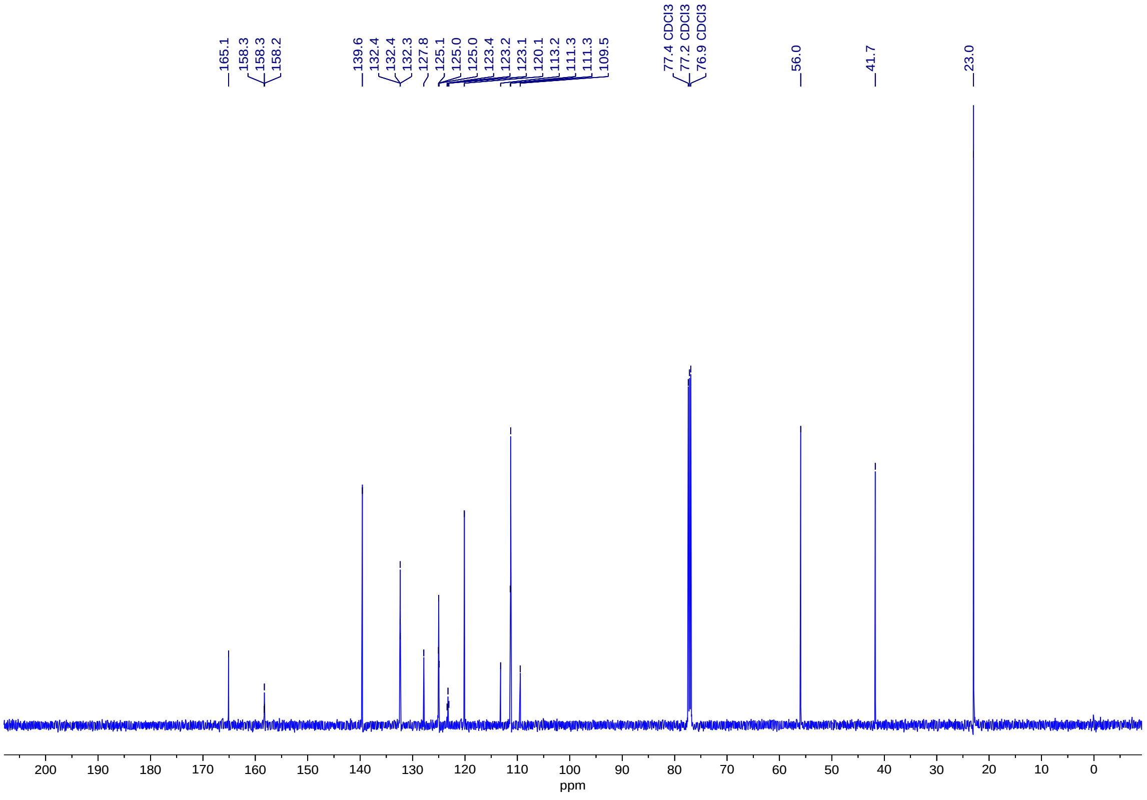
ppm
77.4 CDCl3
77.2 CDCl3
76.9 CDCl3

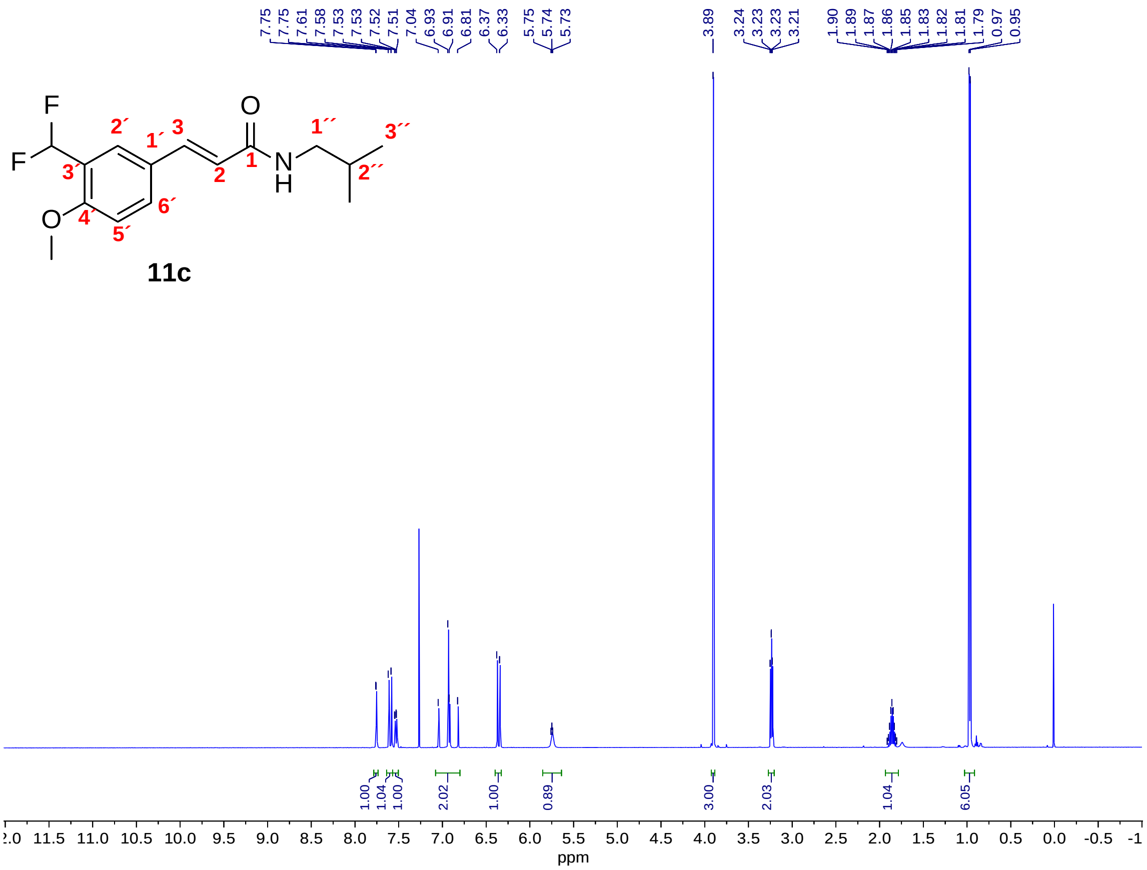
11c
ppm

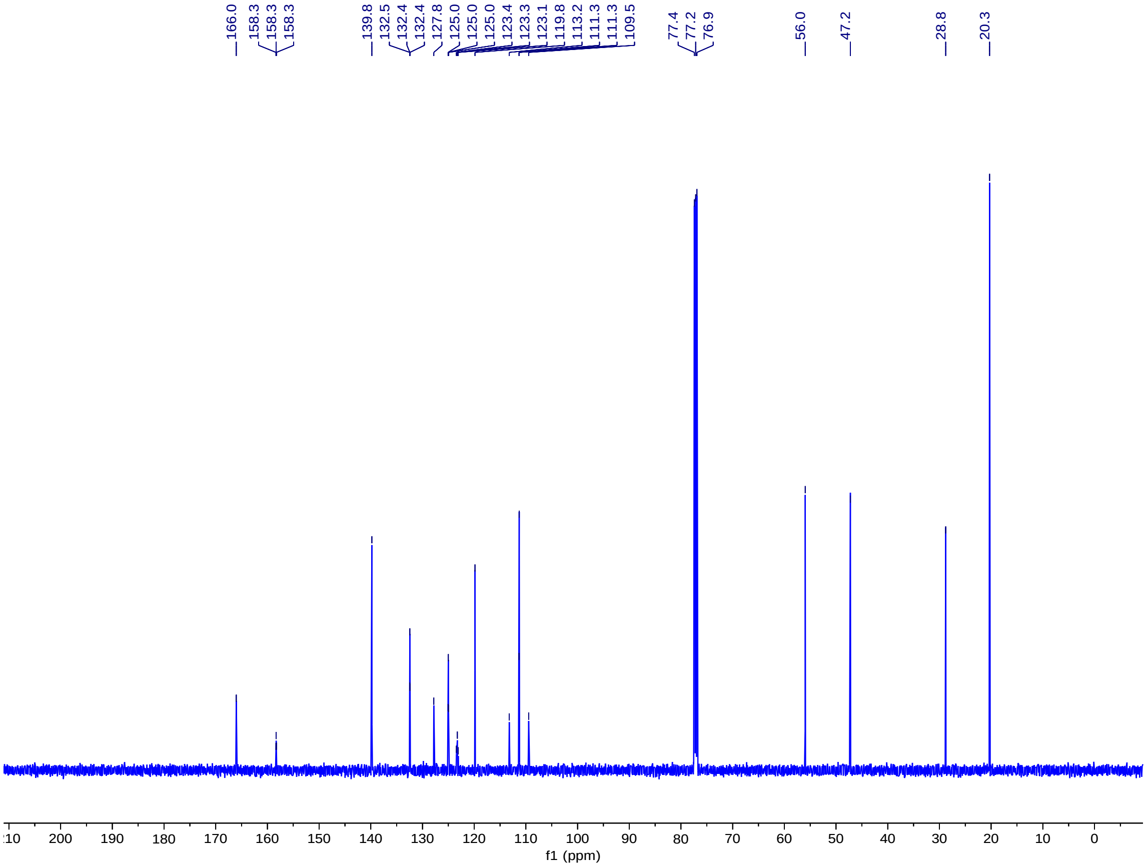
f1 (ppm)

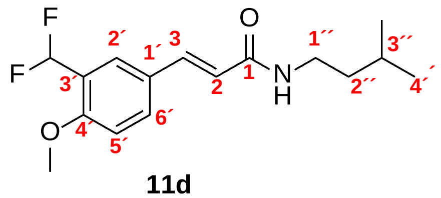

11d

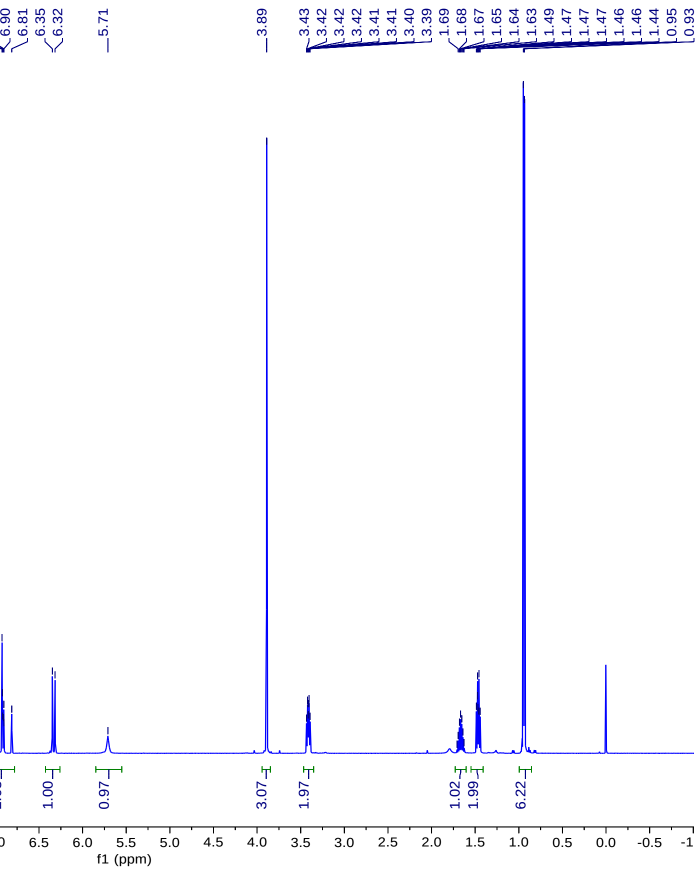
7.74
7.74
7.73
7.60
7.57
7.53
7.52
7.51
7.51
7.27
7.03
6.92
6.92
6.92
6.91
6.90
6.90
6.81
6.35
6.32
5.71
3.89
3.43
3.42
3.42
3.42
3.41
3.40
3.39
1.69
1.68
1.67
1.65
1.64
1.63
1.49
1.47
1.47
1.47
1.46
1.46
1.44
0.95
0.93
1.05
2.06
2.03
1.00
0.97
3.07
1.97
1.02
1.99
6.22
f1 (ppm)

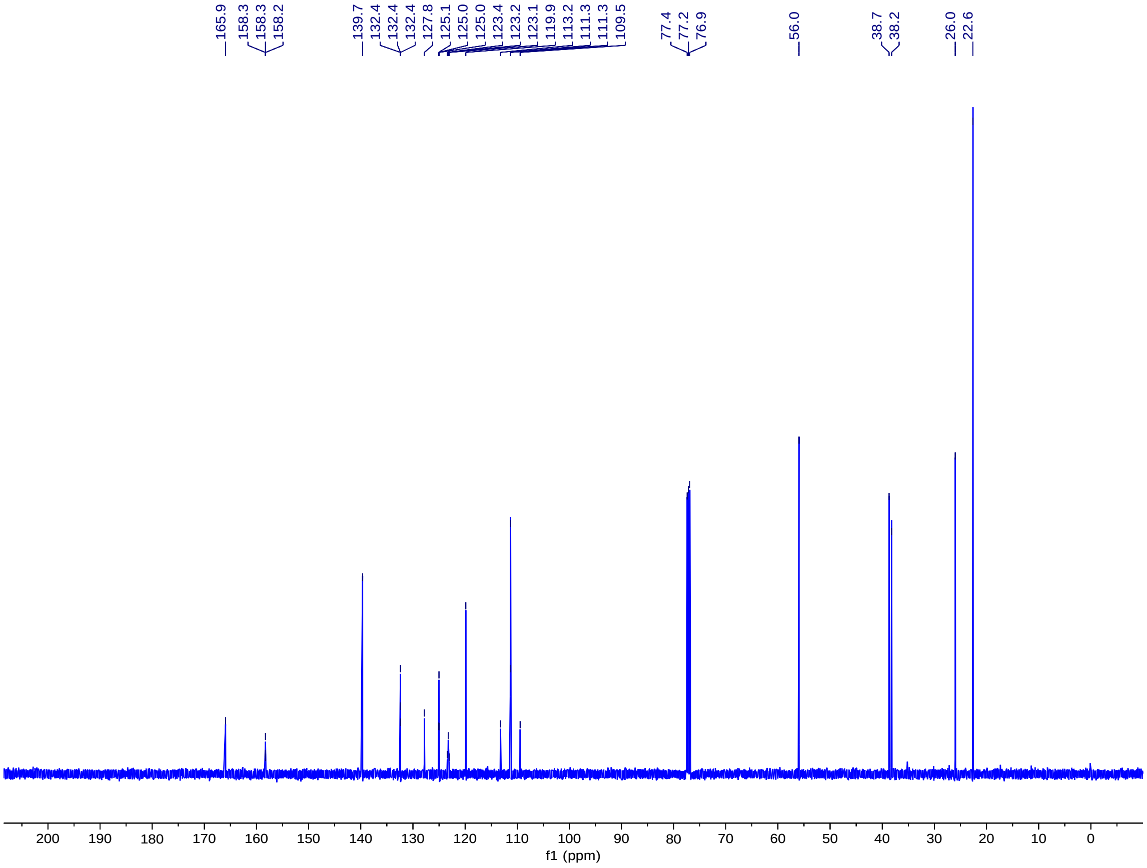
165.9
158.3
158.3
158.2
139.7
132.4
132.4
132.4
127.8
125.1
125.0
125.0
123.4
123.2
123.1
119.9
113.2
111.3
111.3
109.5
77.4
77.2
76.9
56.0
38.7
38.2
26.0
22.6
f1 (ppm)

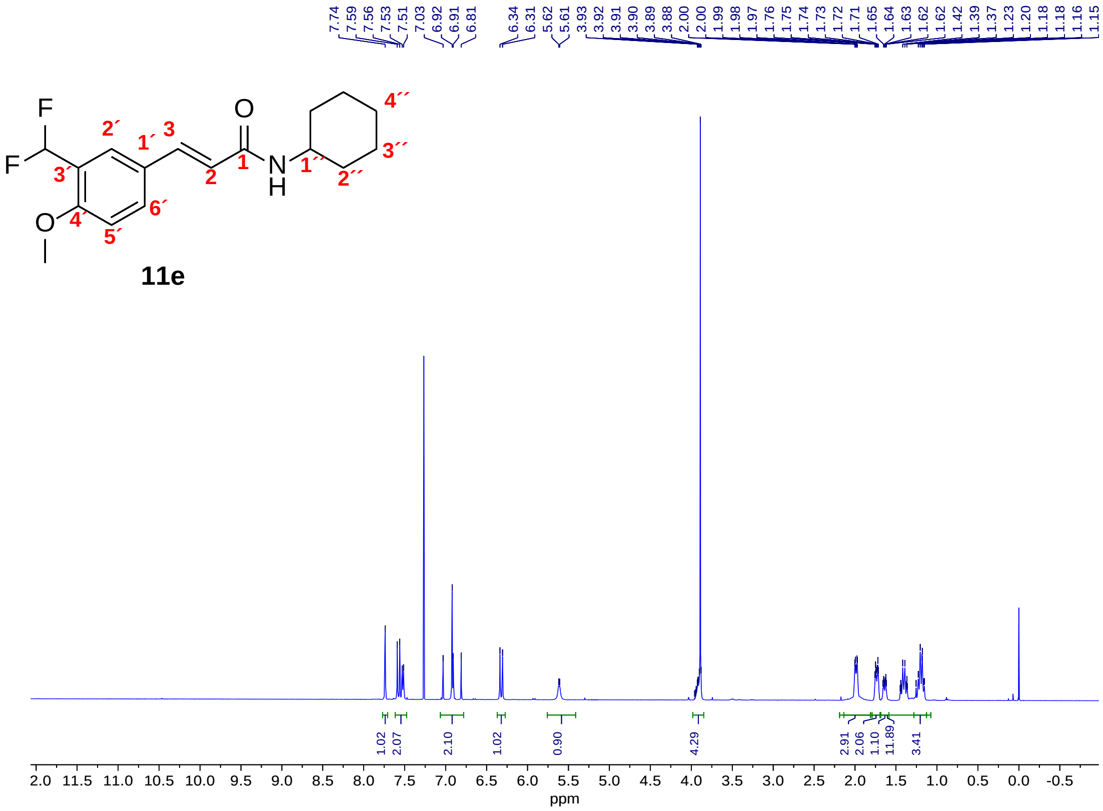
F
F
O
O
N
H
1
2
3
1´
2´
3´
4´
5´
6´
1´´
2´´
3´´
4´´
11e
7.74
7.59
7.56
7.53
7.51
7.03
6.92
6.91
6.81
6.34
6.31
5.62
5.61
3.93
3.92
3.91
3.90
3.89
3.88
2.00
2.00
1.99
1.98
1.97
1.76
1.75
1.74
1.73
1.72
1.71
1.65
1.64
1.63
1.62
1.62
1.42
1.39
1.37
1.23
1.20
1.18
1.18
1.16
1.15
1.02
2.07
2.10
1.02
0.90
4.29
2.91
2.06
1.10
11.89
3.41
ppm

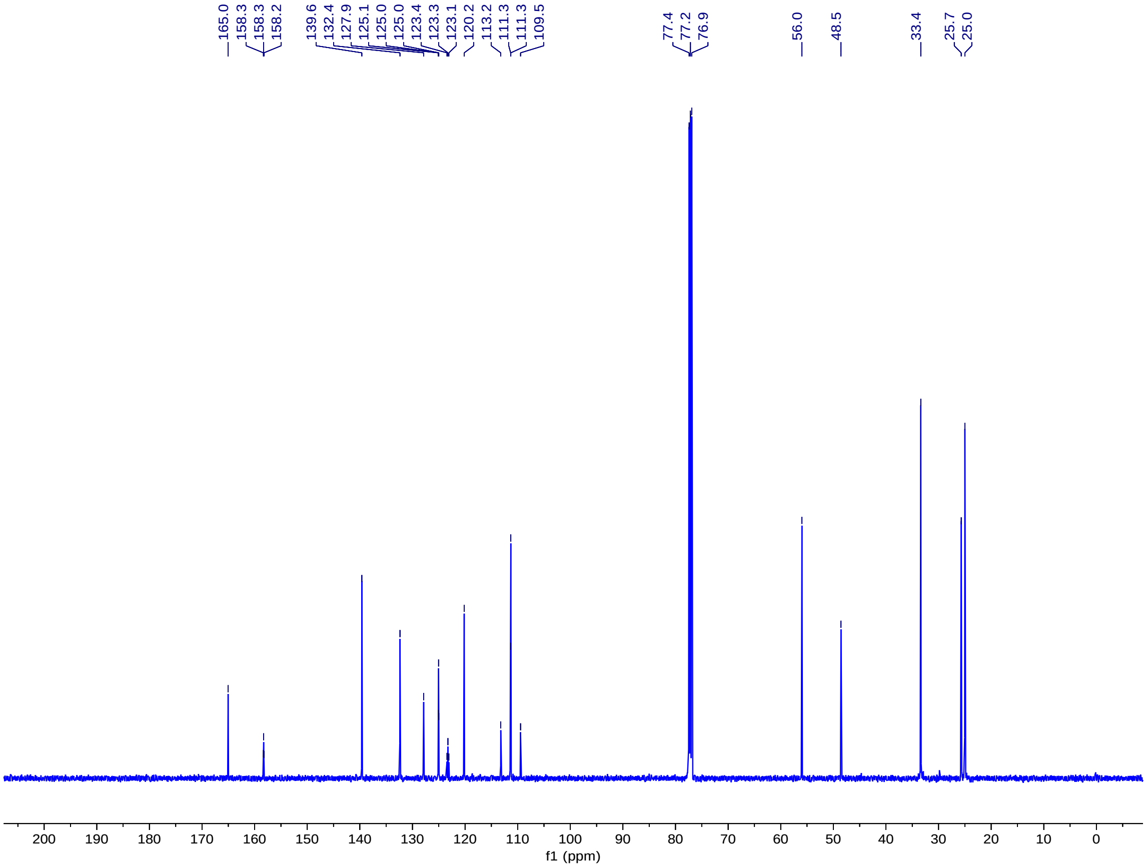
165.0
158.3
158.3
158.2
139.6
132.4
127.9
125.1
125.0
125.0
123.4
123.3
123.1
120.2
113.2
111.3
111.3
109.5
77.4
77.2
76.9
56.0
48.5
33.4
25.7
25.0
f1 (ppm)

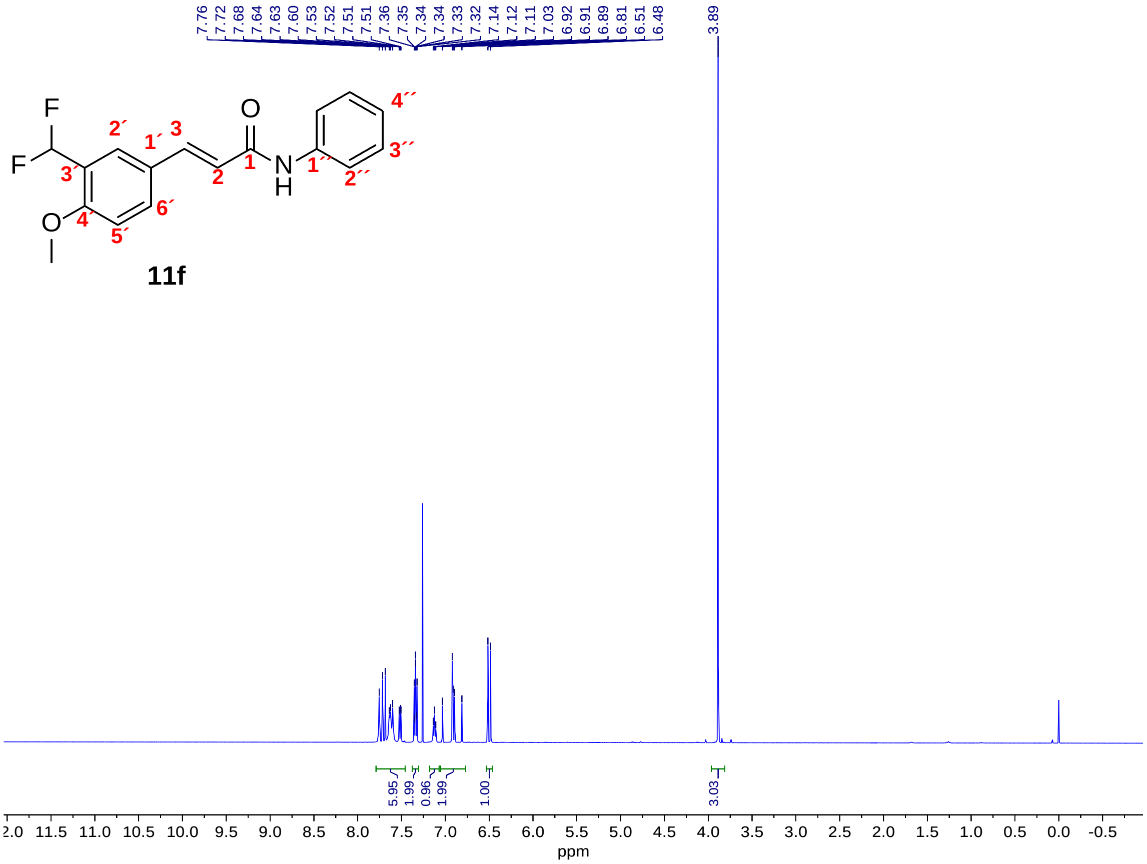
7.76 7.72 7.68 7.64 7.63 7.60 7.53 7.52 7.51 7.51 7.36 7.35 7.34 7.34 7.33 7.32 7.14 7.12 7.11 7.03 6.92 6.91 6.89 6.81 6.51 6.48
3.89
F
F
O
N
H
O
1
2
3
1´
2´
3´
4´
5´
6´
1´´
2´´
3´´
4´´
11f
5.95 1.99 0.96 1.99 1.00 3.03
ppm

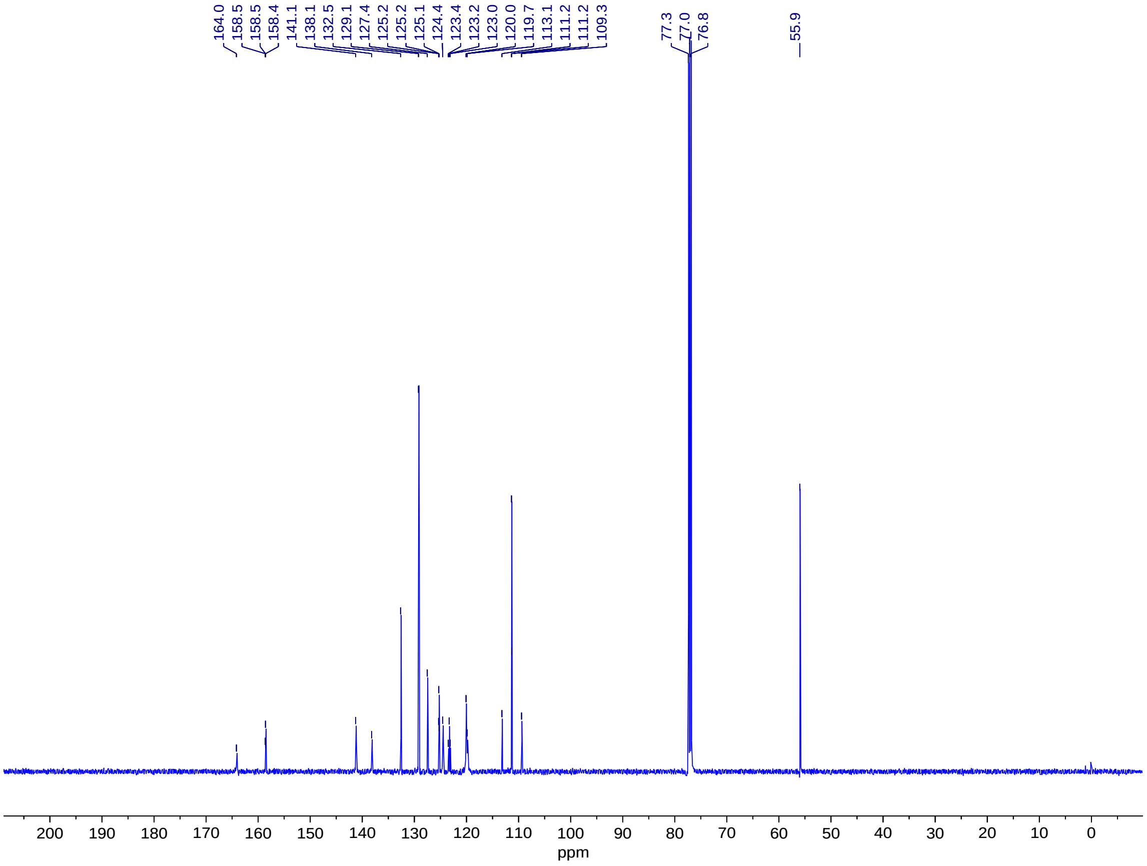
164.0 158.5 158.5 158.4 141.1 138.1 132.5 129.1 127.4 125.2 125.2 125.1 124.4 123.4 123.2 123.0 120.0 119.7 113.1 111.2 111.2 109.3
77.3 77.0 76.8
55.9
ppm

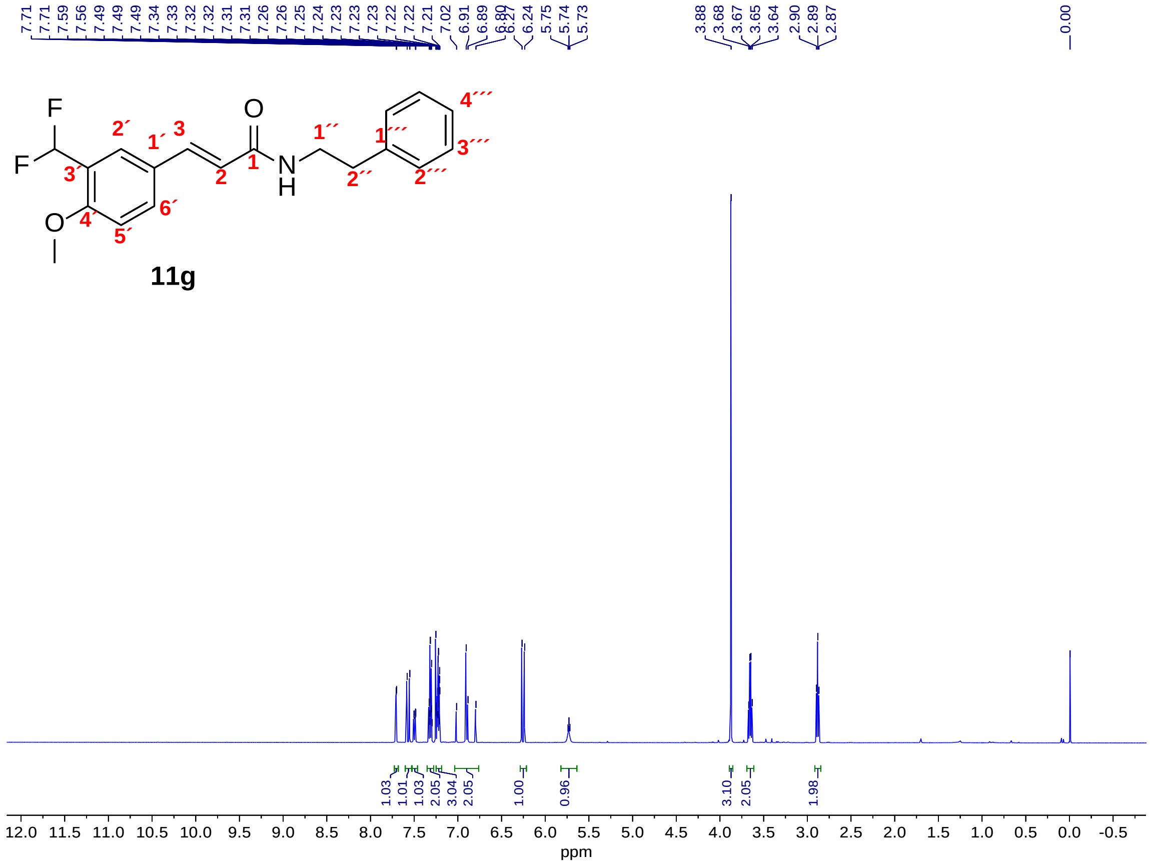

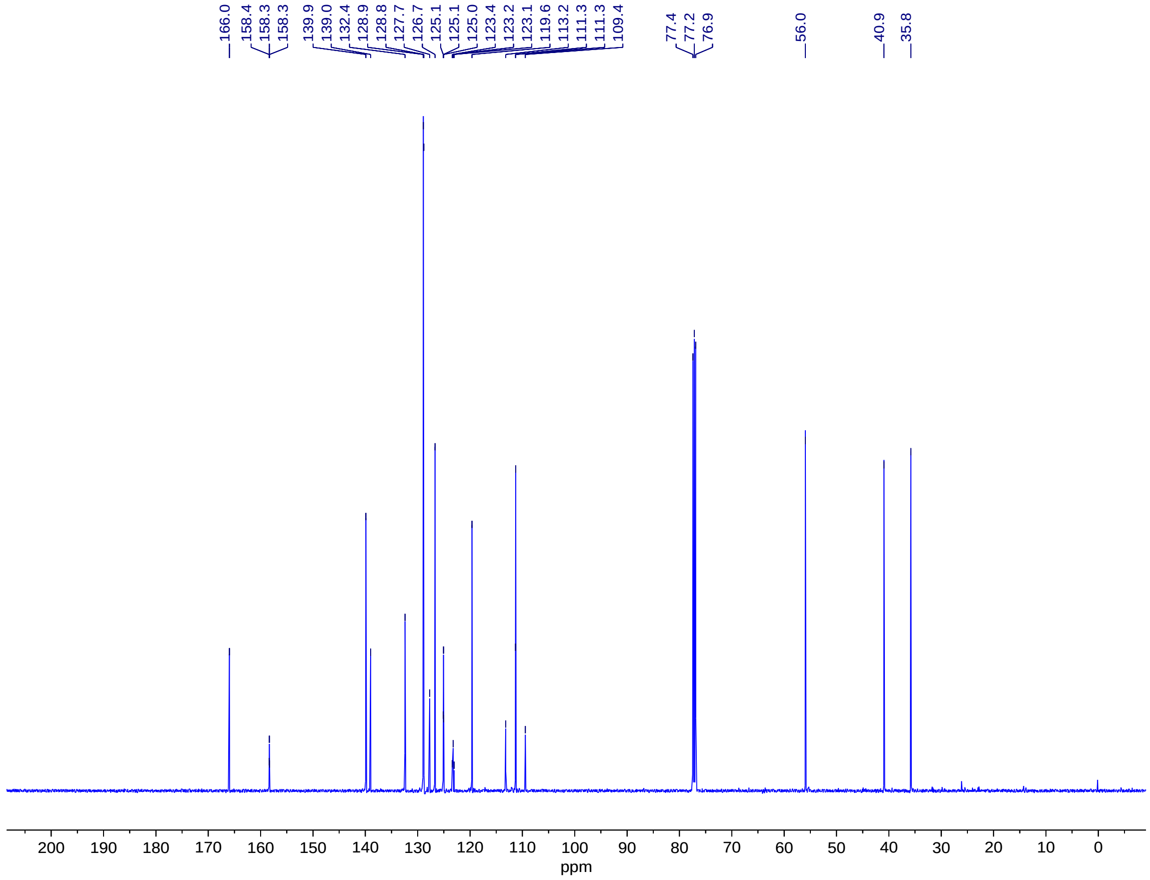

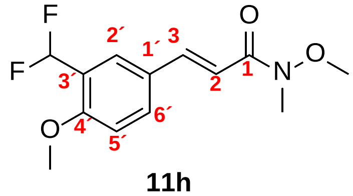
F
F
O
N
O
O
1
2
3
1´
2´
3´
4´
5´
6´

11h

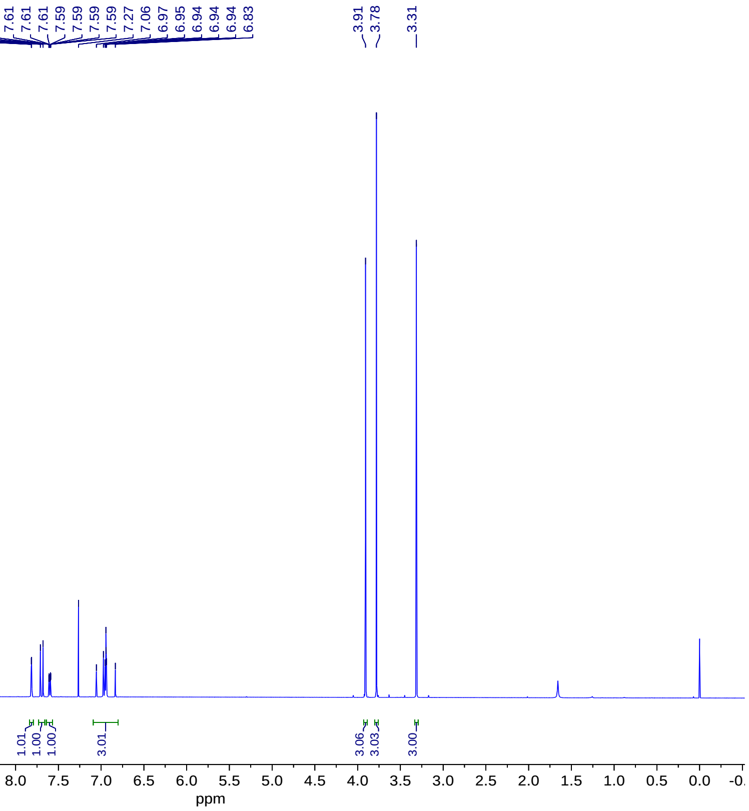
7.82
7.82
7.81
7.71
7.68
7.61
7.61
7.61
7.61
7.61
7.59
7.59
7.59
7.59
7.27
7.06
6.97
6.95
6.94
6.94
6.94
6.83
3.91
3.78
3.31
1.01
1.00
1.00
3.01
3.06
3.03
3.00
ppm

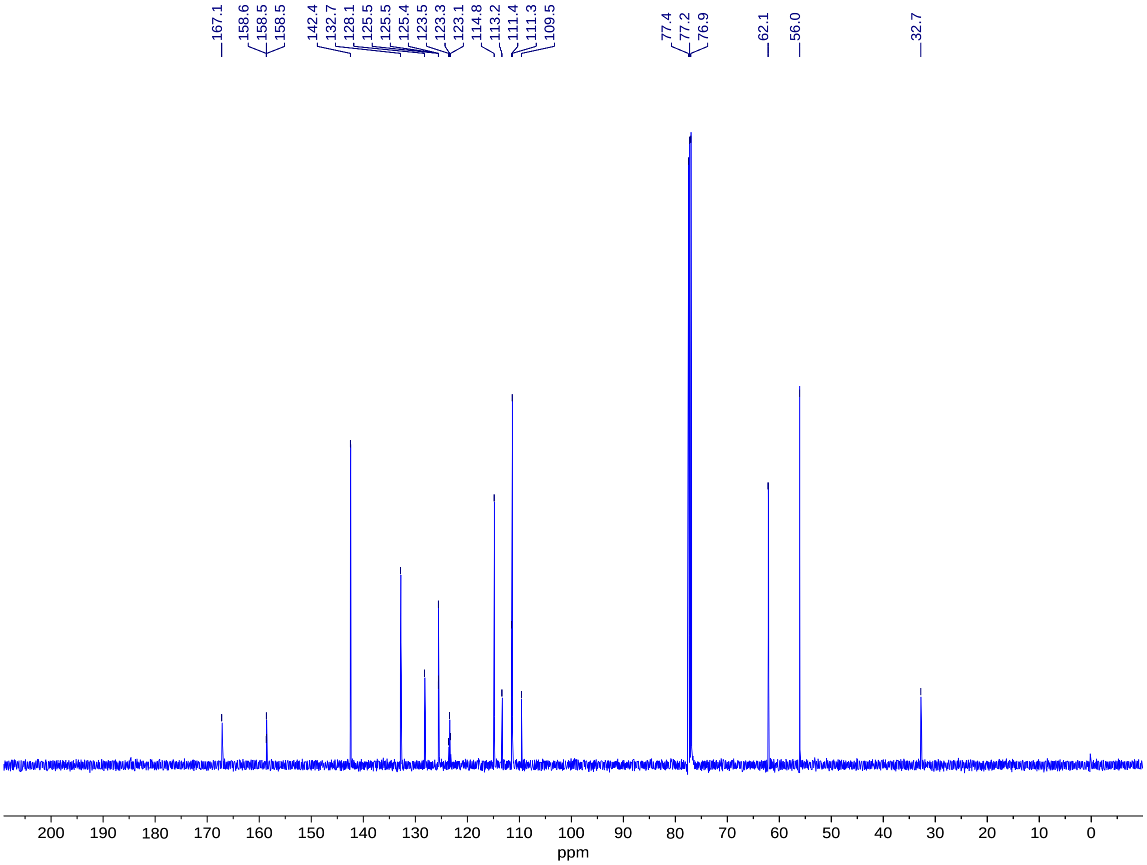
167.1
158.6
158.5
158.5
142.4
132.7
128.1
125.5
125.5
125.4
123.5
123.3
123.1
114.8
113.2
111.4
111.3
109.5
77.4
77.2
76.9
62.1
56.0
32.7
ppm

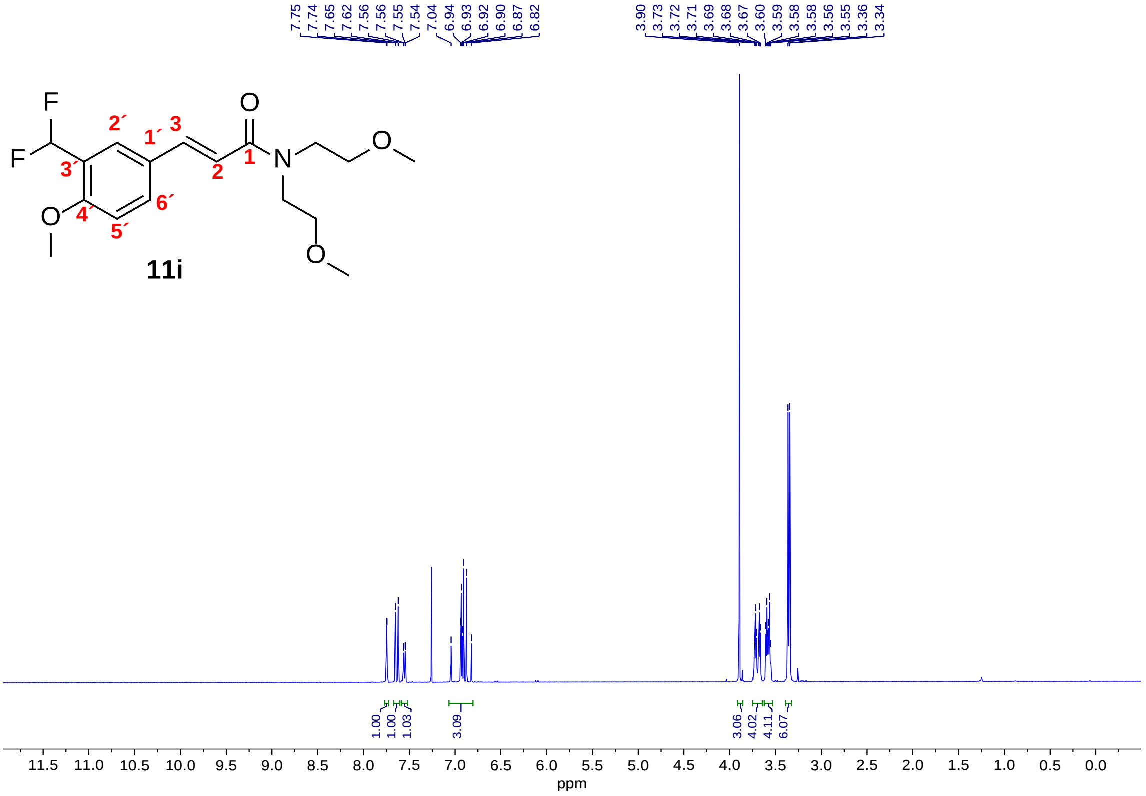
F
F
O
O
N
O
O
1
2
3
1´
2´
3´
4´
5´
6´
11i
7.75 7.74 7.65 7.62 7.56 7.56 7.55 7.54 7.04 6.94 6.93 6.92 6.90 6.87 6.82
3.90 3.73 3.72 3.71 3.69 3.68 3.67 3.60 3.59 3.58 3.58 3.56 3.55 3.36 3.34
1.00 1.00 1.03 3.09 3.06 4.02 4.11 6.07
ppm

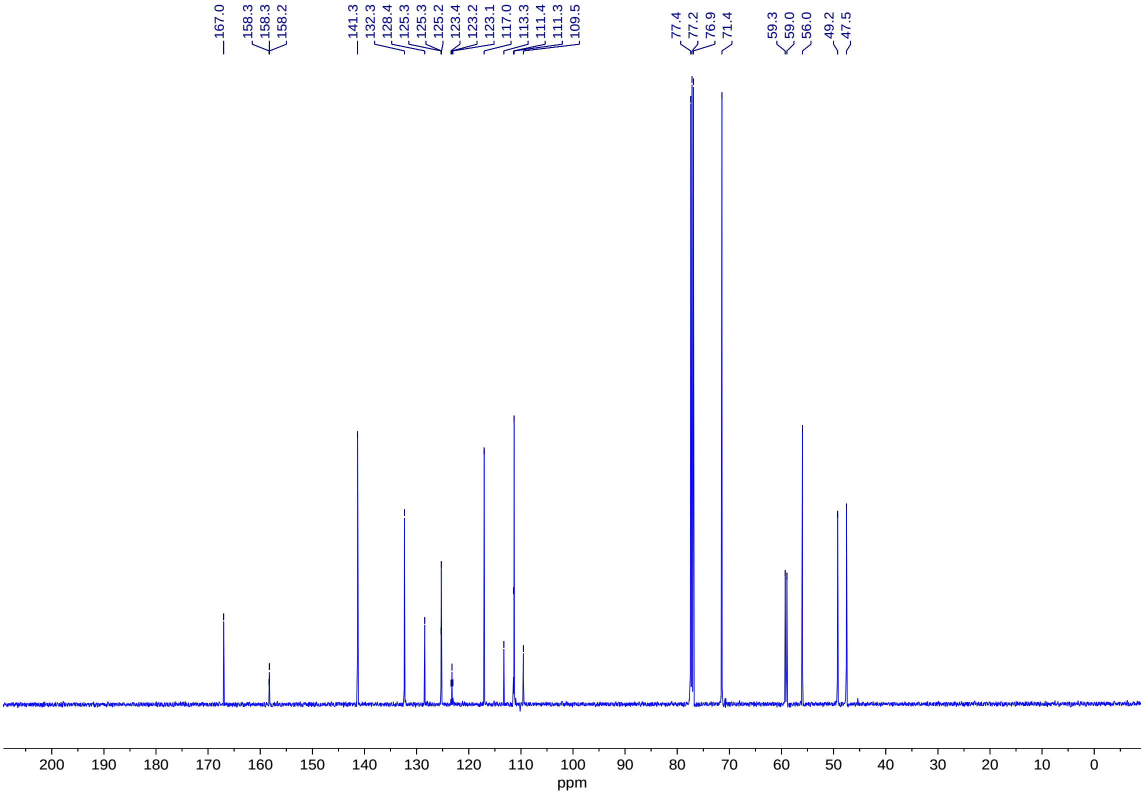
167.0 158.3 158.3 158.2 141.3 132.3 128.4 125.3 125.3 125.2 123.4 123.2 123.1 117.0 113.3 111.4 111.3 109.5
77.4 77.2 76.9 71.4 59.3 59.0 56.0 49.2 47.5
ppm

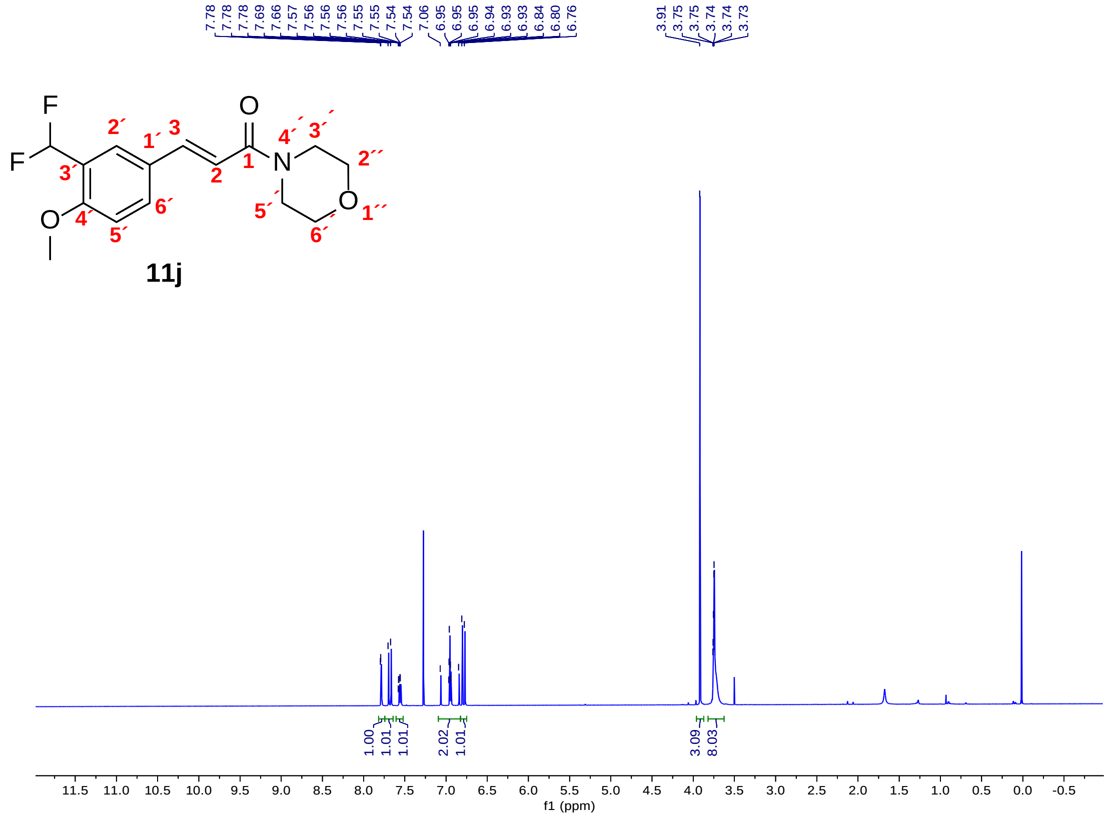
7.78 7.78 7.78 7.69 7.66 7.57 7.56 7.56 7.56 7.55 7.55 7.54 7.54 7.06 6.95 6.95 6.95 6.94 6.93 6.93 6.84 6.80 6.76
3.91 3.75 3.75 3.74 3.74 3.73
11j
1.00 1.01 1.01 2.02 1.01
3.09 8.03
f1 (ppm)

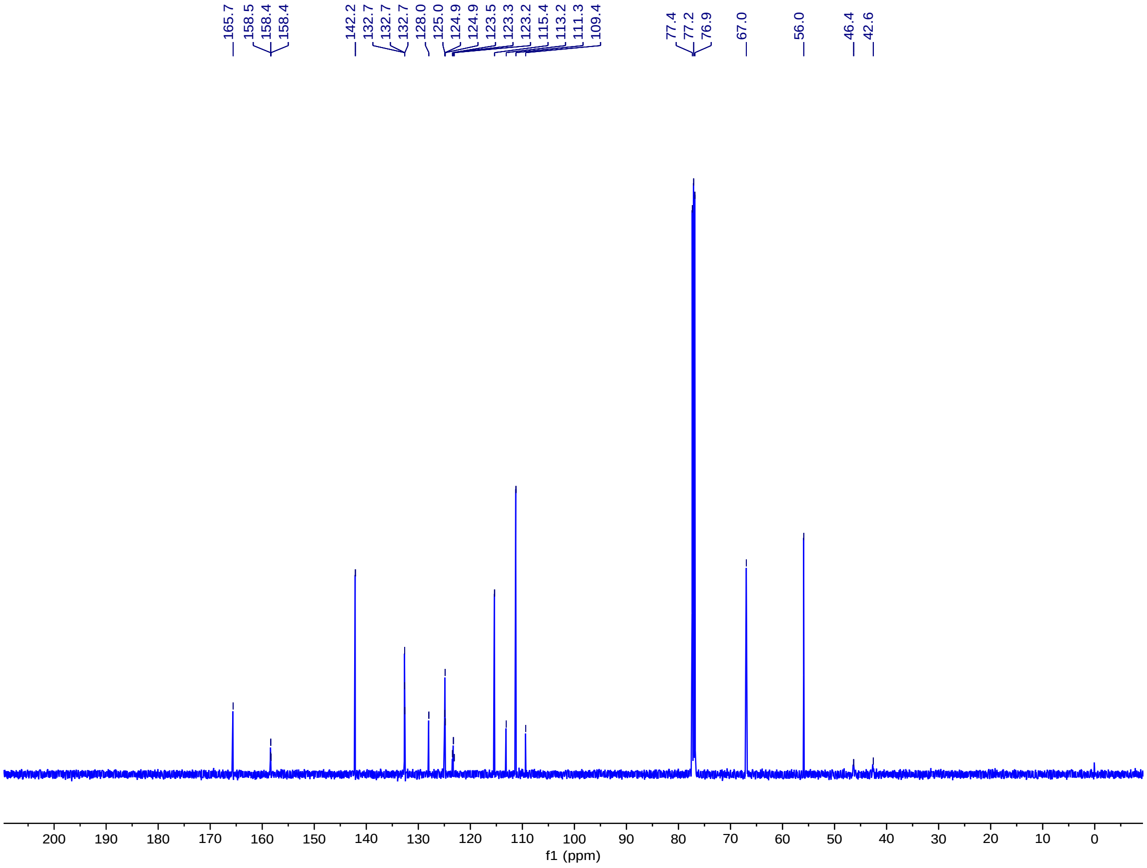
165.7 158.5 158.4 158.4
142.2 132.7 132.7 132.7 128.0 125.0 124.9 124.9 123.5 123.3 123.2 115.4 113.2 111.3 109.4
77.4 77.2 76.9 67.0 56.0 46.4 42.6
f1 (ppm)

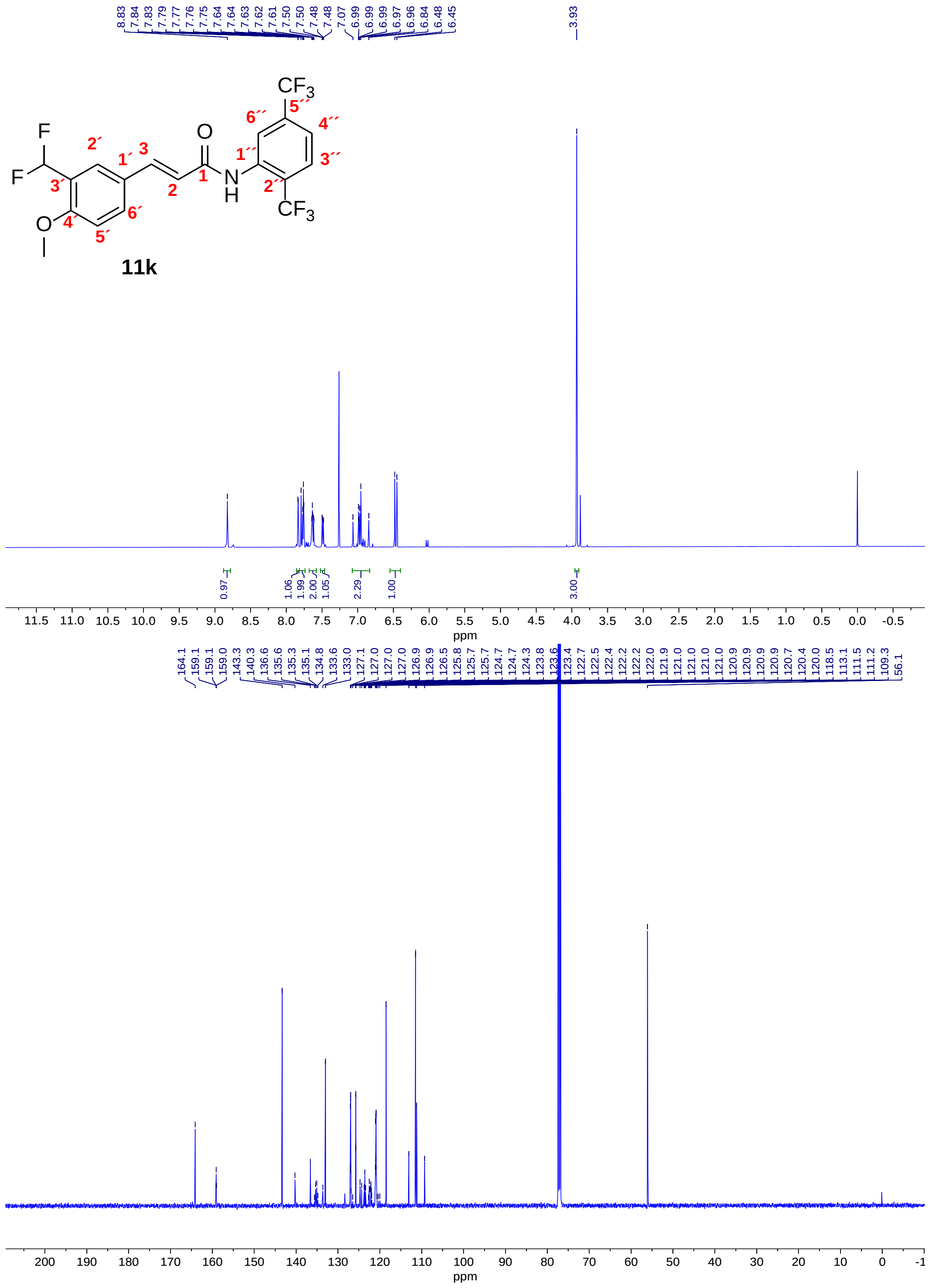

11k
ppm
ppm

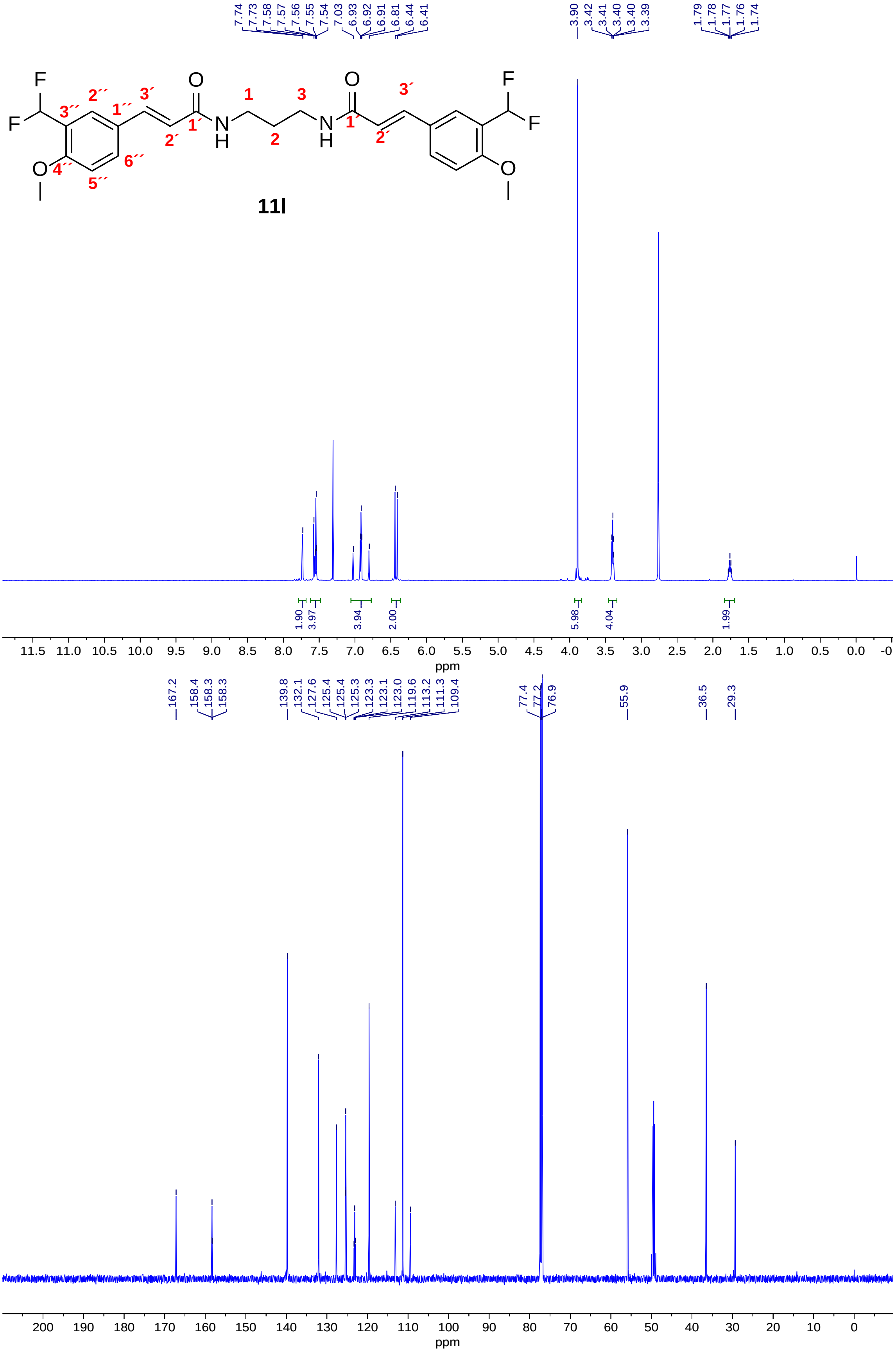
7.74 7.73 7.58 7.57 7.56 7.55 7.54 7.03 6.93 6.92 6.91 6.81 6.44 6.41
3.90 3.42 3.41 3.40 3.40 3.39
1.79 1.78 1.77 1.76 1.74
F
F
O
O
F
F
O
O
N
H
N
H
1
2
3
1´
2´
3´
1´
2´
3´
1´´
2´´
3´´
4´´
5´´
6´´
11l
1.90 3.97 3.94 2.00 5.98 4.04 1.99
11.5 11.0 10.5 10.0 9.5 9.0 8.5 8.0 7.5 7.0 6.5 6.0 5.5 5.0 4.5 4.0 3.5 3.0 2.5 2.0 1.5 1.0 0.5 0.0 -0
ppm
167.2 158.4 158.3 158.3 139.8 132.1 127.6 125.4 125.4 125.3 123.3 123.1 123.0 119.6 113.2 111.3 109.4 77.4 77.2 76.9 55.9 36.5 29.3
200 190 180 170 160 150 140 130 120 110 100 90 80 70 60 50 40 30 20 10 0
ppm

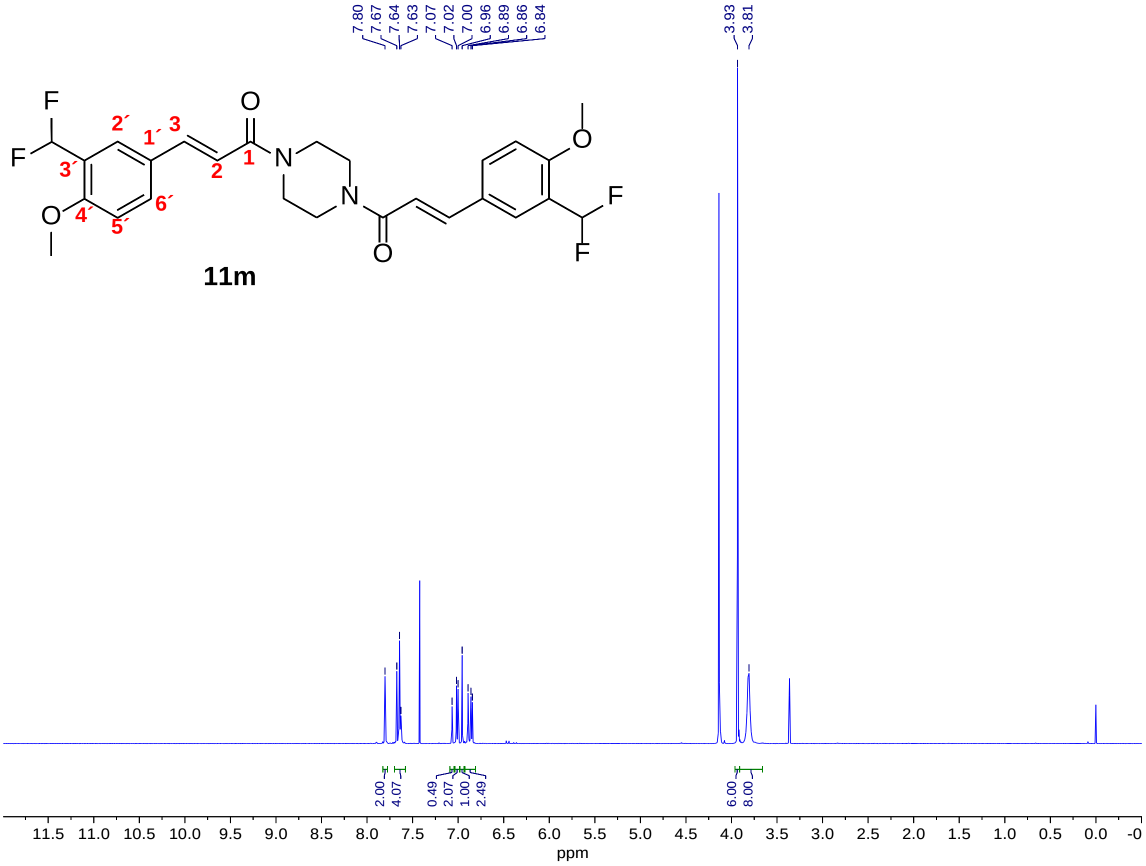

11m
7.80
7.67
7.64
7.63
7.07
7.02
7.00
6.96
6.89
6.86
6.84
3.93
3.81
2.00
4.07
0.49
2.07
1.00
2.49
6.00
8.00
ppm

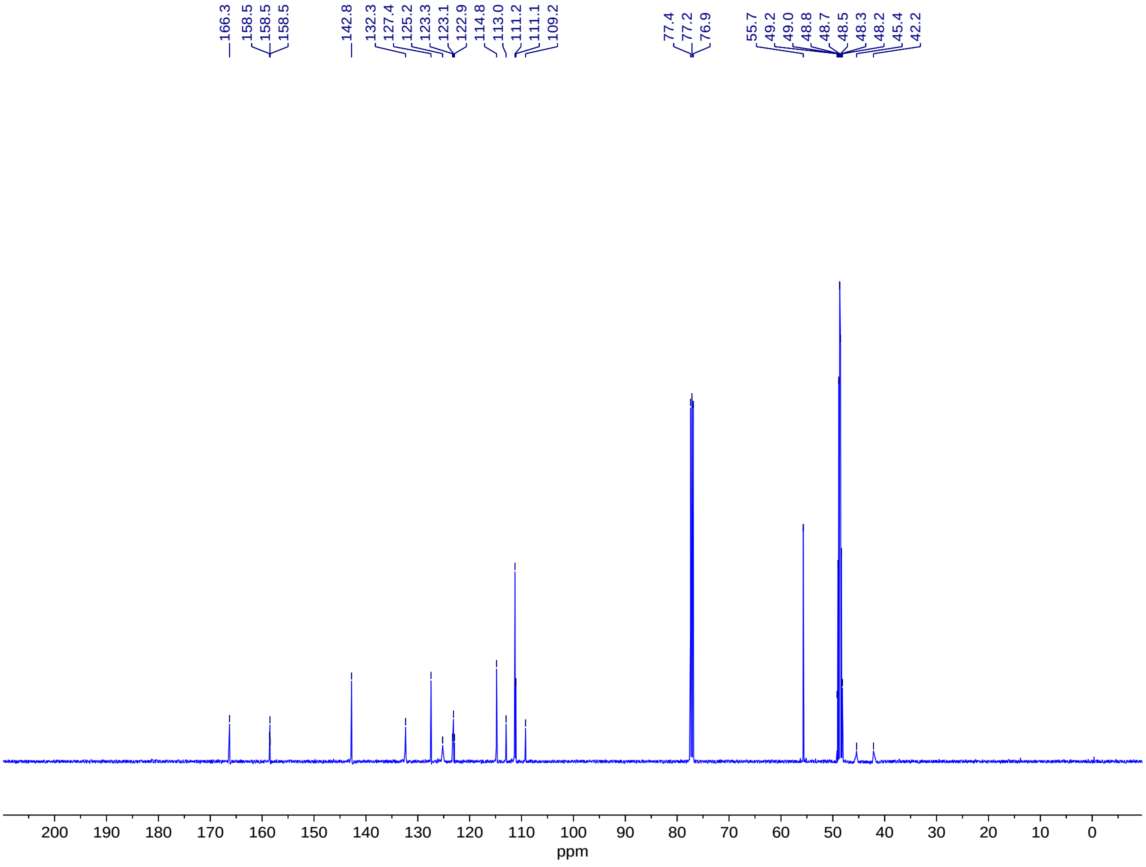

166.3
158.5
158.5
158.5
142.8
132.3
127.4
125.2
123.3
123.1
122.9
114.8
113.0
111.2
111.1
109.2
77.4
77.2
76.9
55.7
49.2
49.0
48.8
48.7
48.5
48.3
48.2
45.4
42.2
ppm

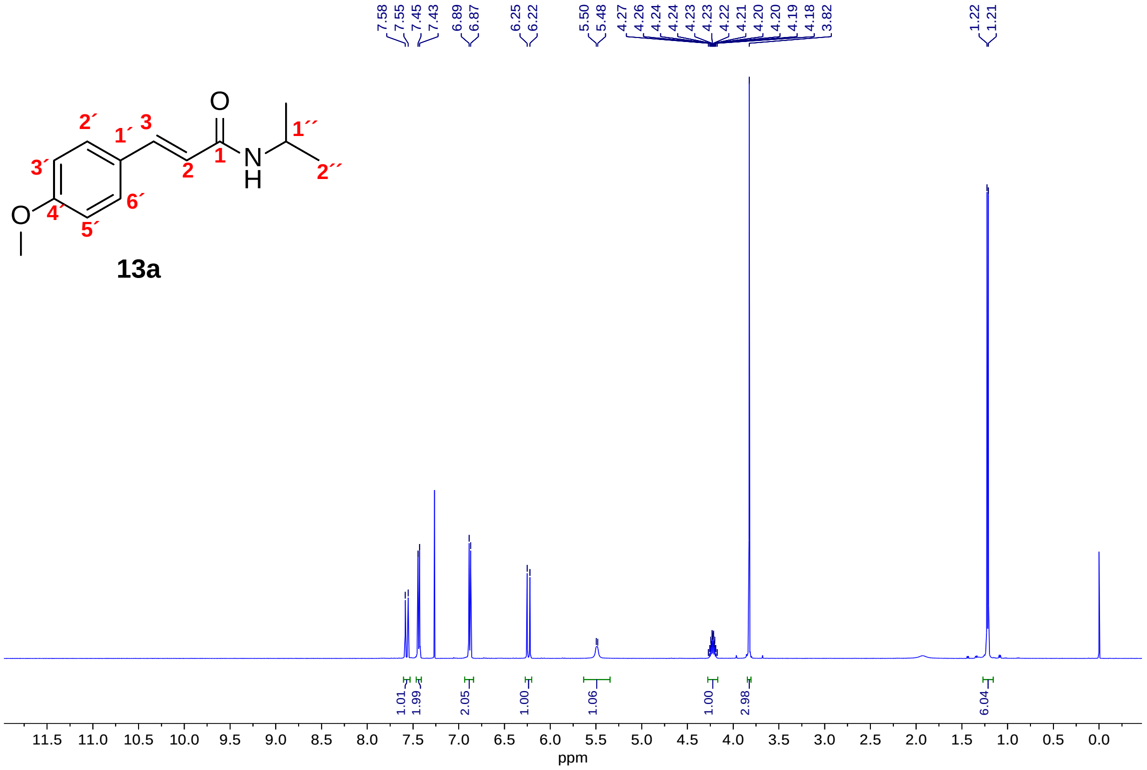
O
N
H
O
1
2
3
1´
2´
3´
4´
5´
6´
1´´
2´´
13a

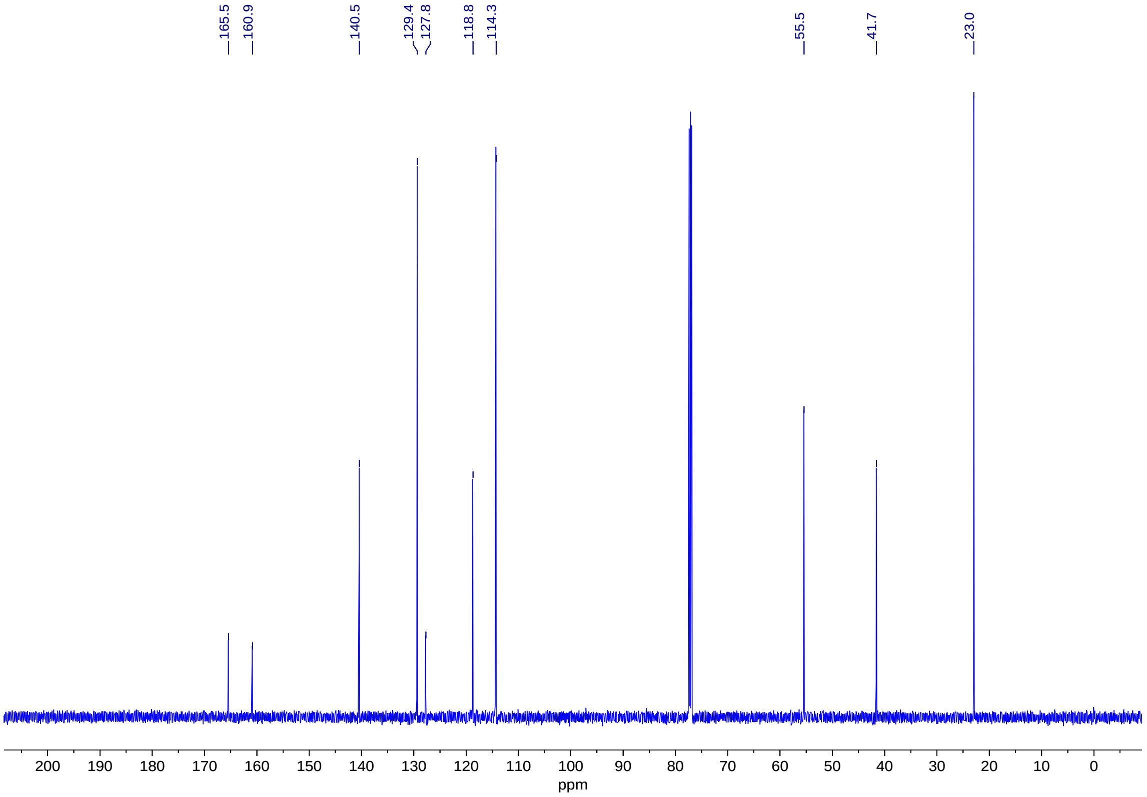

13b

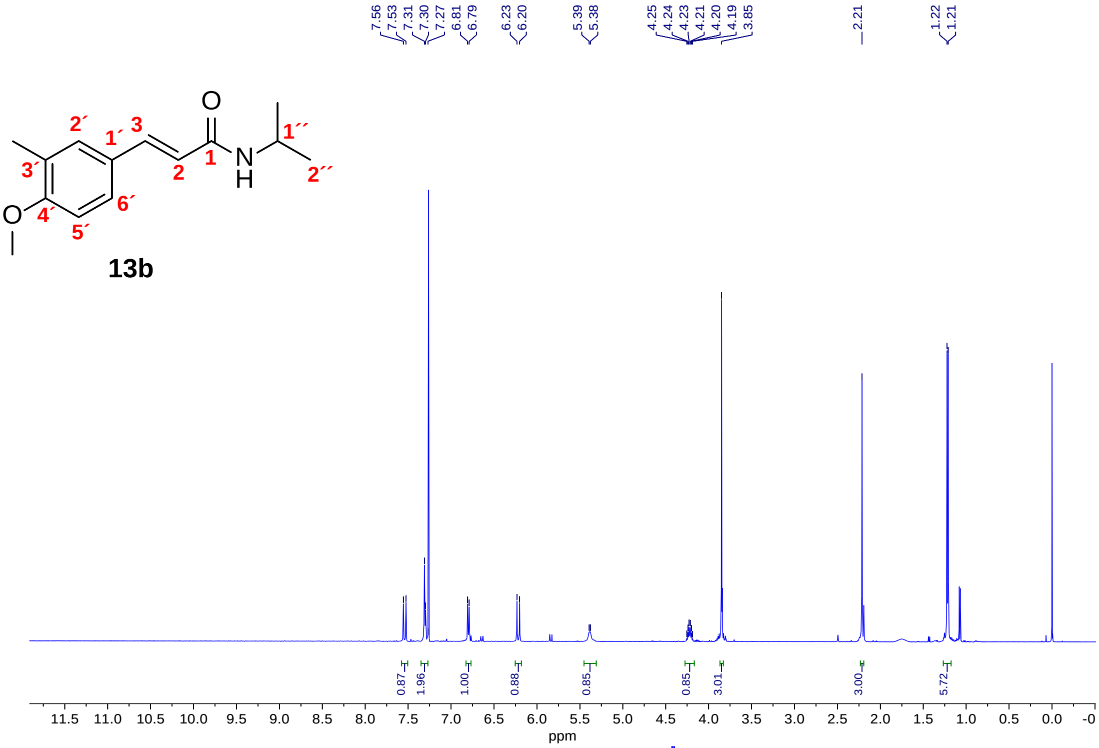
O
N
H
1
2
3
1´
2´
3´
4´
5´
6´
1´´
2´´
O
7.56
7.53
7.31
7.30
7.27
6.81
6.79
6.23
6.20
5.39
5.38
4.25
4.24
4.23
4.21
4.20
4.19
3.85
2.21
1.22
1.21
0.87
1.96
1.00
0.88
0.85
0.85
3.01
3.00
5.72
11.5 11.0 10.5 10.0 9.5 9.0 8.5 8.0 7.5 7.0 6.5 6.0 5.5 5.0 4.5 4.0 3.5 3.0 2.5 2.0 1.5 1.0 0.5 0.0 -0
ppm

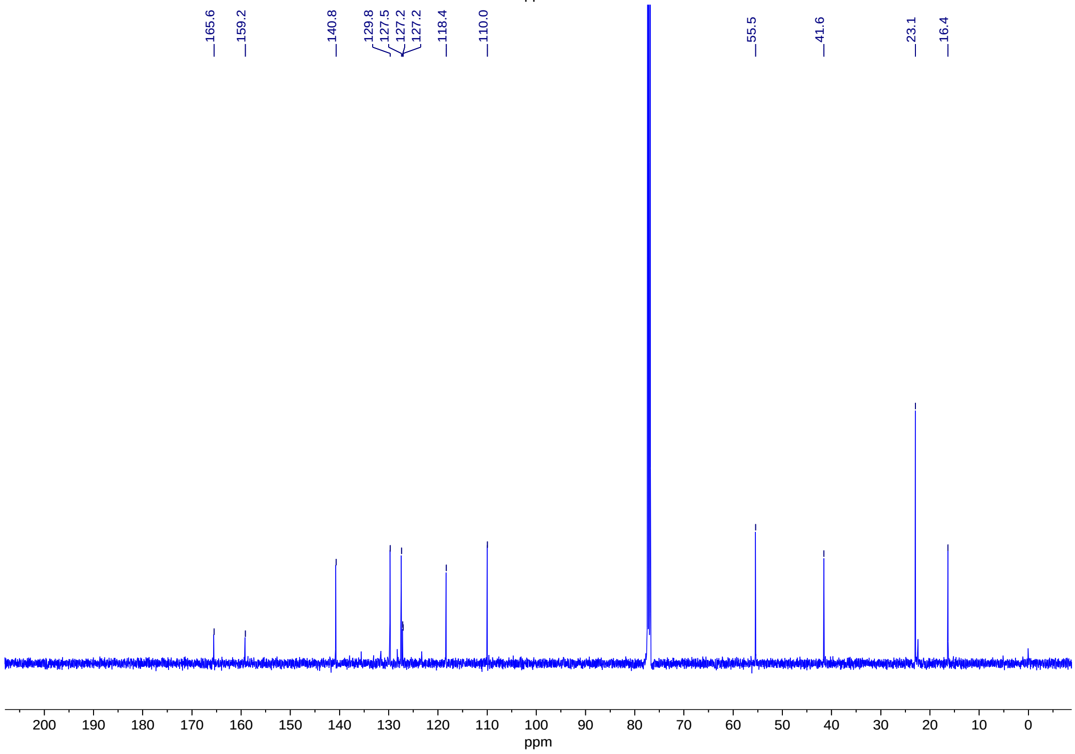
165.6
159.2
140.8
129.8
127.5
127.2
127.2
118.4
110.0
55.5
41.6
23.1
16.4
200 190 180 170 160 150 140 130 120 110 100 90 80 70 60 50 40 30 20 10 0
ppm

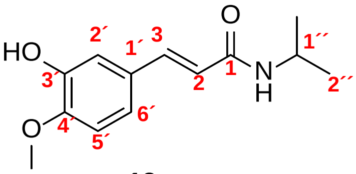

HO
O
O
N
H
1´
2´
3
3´
4´
5´
6´
2
1
1´´
2´´

13c

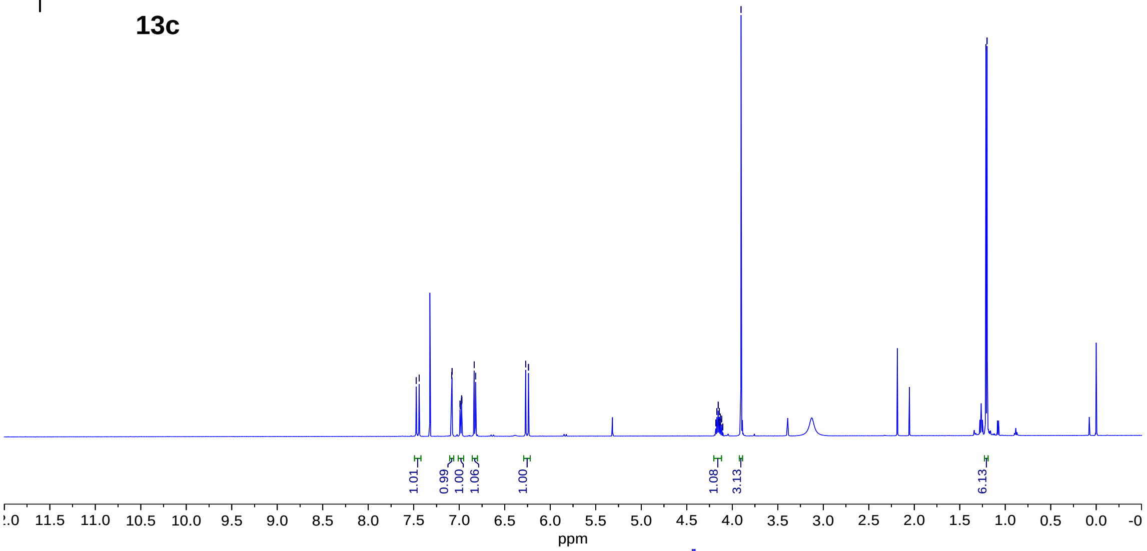

7.47
7.44
7.08
7.08
6.99
6.99
6.98
6.97
6.97
6.84
6.82
6.27
6.24
4.18
4.17
4.15
4.15
4.14
4.13
4.13
4.12
4.10
3.90
1.21
1.20
1.01
0.99
1.00
1.06
1.00
1.08
3.13
6.13
ppm

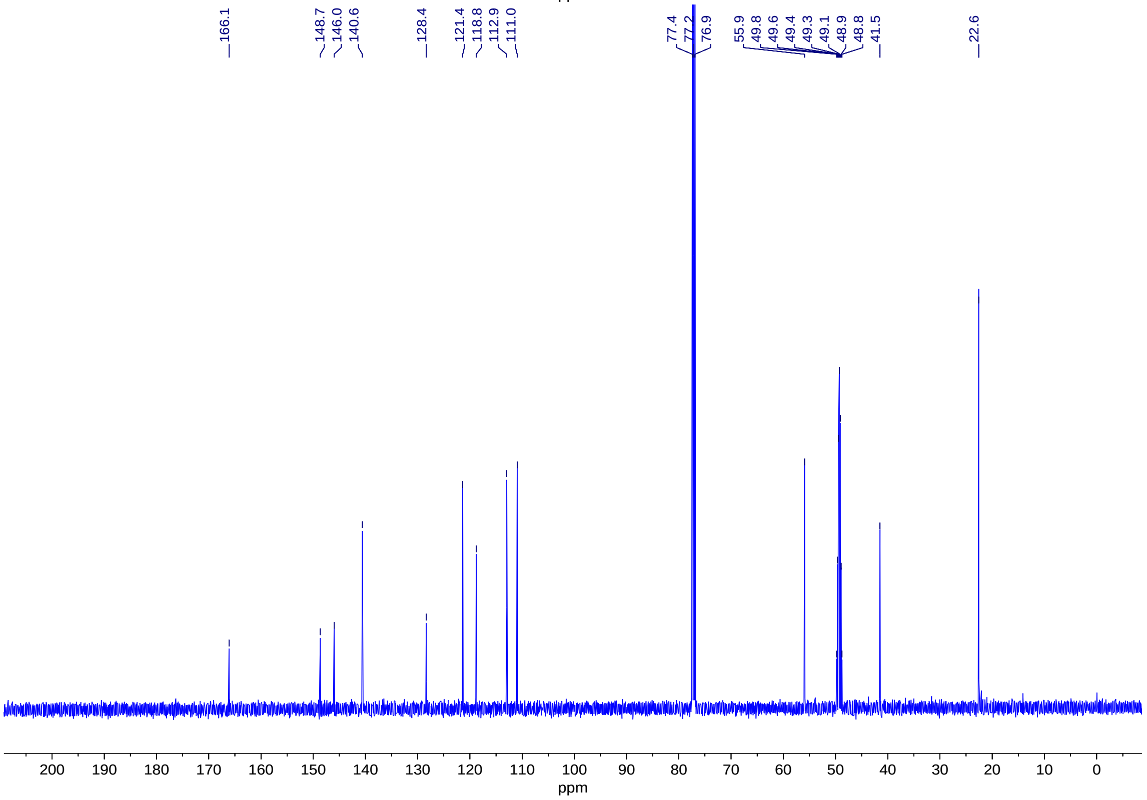

166.1
148.7
146.0
140.6
128.4
121.4
118.8
112.9
111.0
77.4
77.2
76.9
55.9
49.8
49.6
49.4
49.3
49.1
48.9
48.8
41.5
22.6
ppm

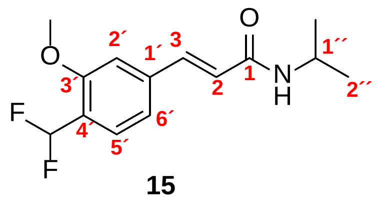
1´
2´
3´
4´
5´
6´
1
2
3
1´´
2´´
O
N
H
F

15

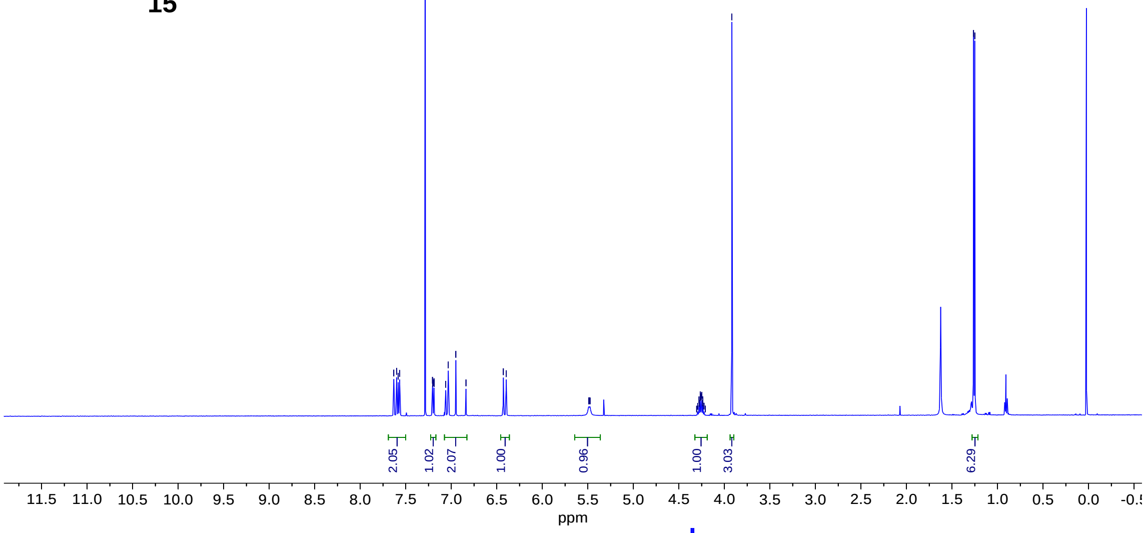
7.63 7.60 7.58 7.57 7.21 7.20 7.19 7.19 7.19 7.06 7.03 6.95 6.84 6.43 6.40 5.49 5.48 5.48 4.30 4.29 4.28 4.28 4.27 4.26 4.25 4.25 4.24 4.24 4.22 4.21 3.92 1.26 1.25
2.05 1.02 2.07 1.00 0.96 1.00 3.03 6.29
ppm

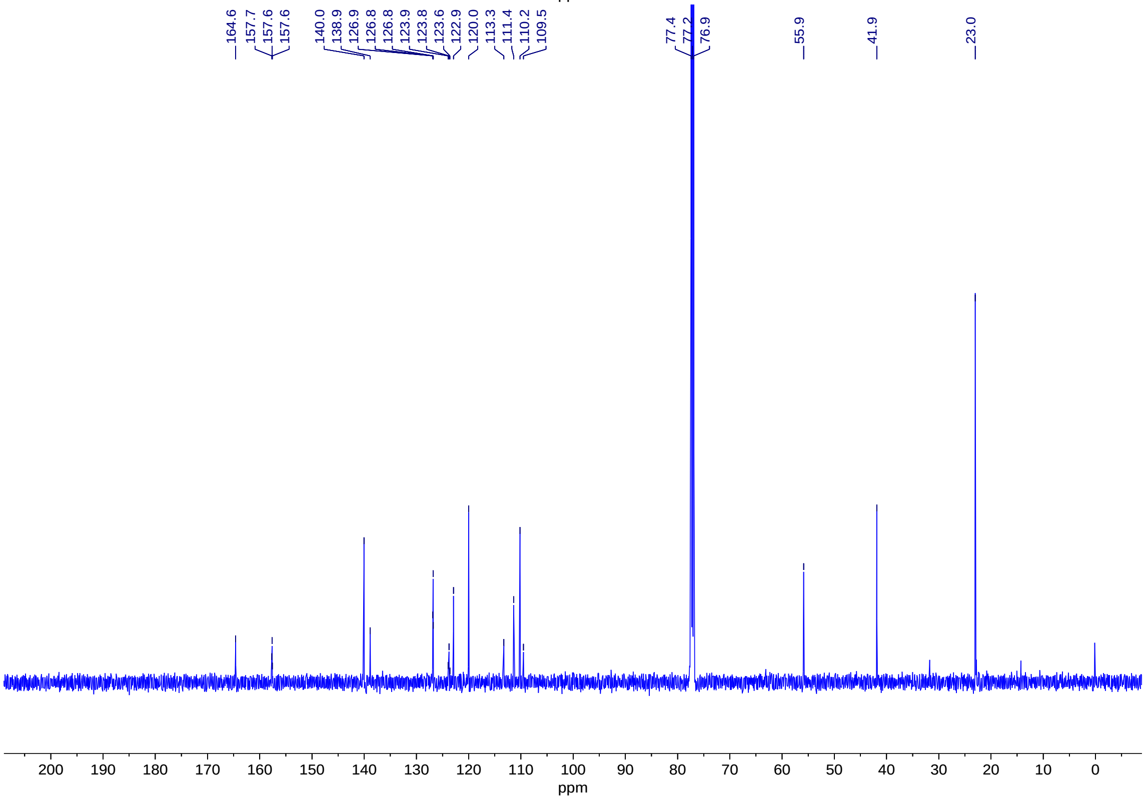
164.6 157.7 157.6 157.6 140.0 138.9 126.9 126.8 123.9 123.8 123.6 122.9 120.0 113.3 111.4 110.2 109.5 77.4 77.2 76.9 55.9 41.9 23.0
ppm

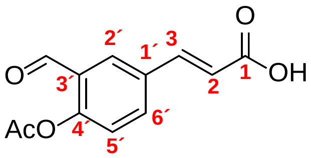
O
AcO
OH
O
1´
2´
3´
4´
5´
6´
1
2
3

17

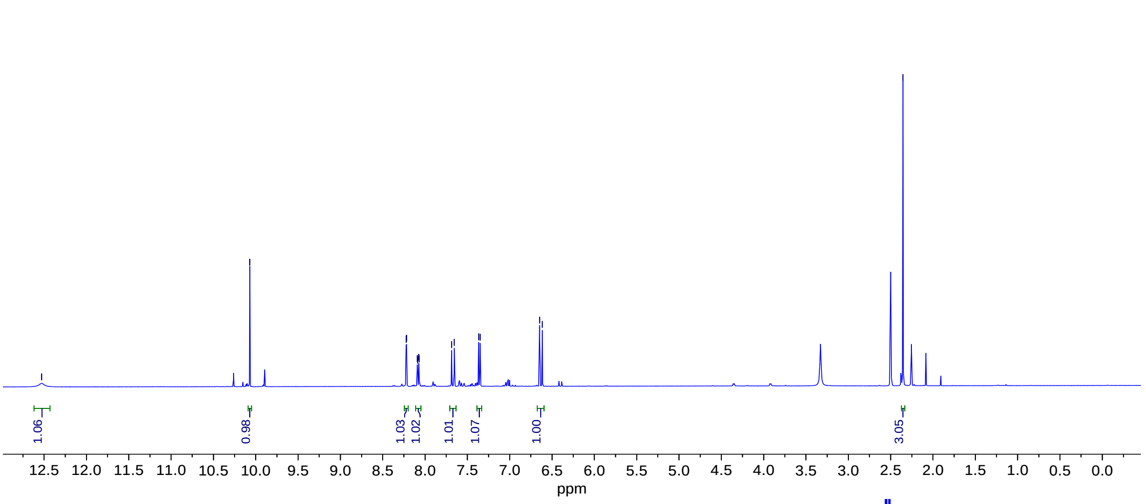
12.53
10.07
8.22
8.22
8.09
8.09
8.07
8.07
7.69
7.65
7.37
7.35
6.65
6.61
2.35
1.06
0.98
1.03
1.02
1.01
1.07
1.00
3.05
12.5 12.0 11.5 11.0 10.5 10.0 9.5 9.0 8.5 8.0 7.5 7.0 6.5 6.0 5.5 5.0 4.5 4.0 3.5 3.0 2.5 2.0 1.5 1.0 0.5 0.0
ppm

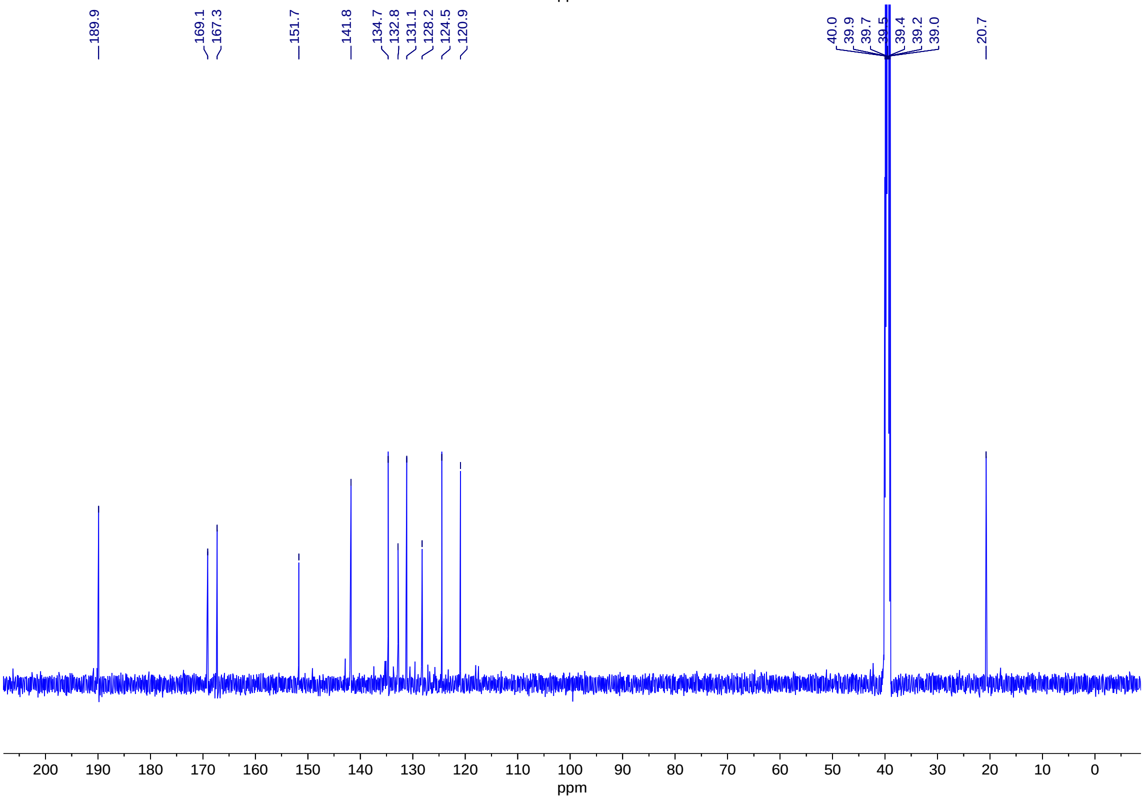
189.9
169.1
167.3
151.7
141.8
134.7
132.8
131.1
128.2
124.5
120.9
40.0
39.9
39.7
39.5
39.4
39.2
39.0
20.7
200 190 180 170 160 150 140 130 120 110 100 90 80 70 60 50 40 30 20 10 0
ppm

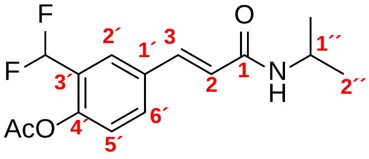

18a

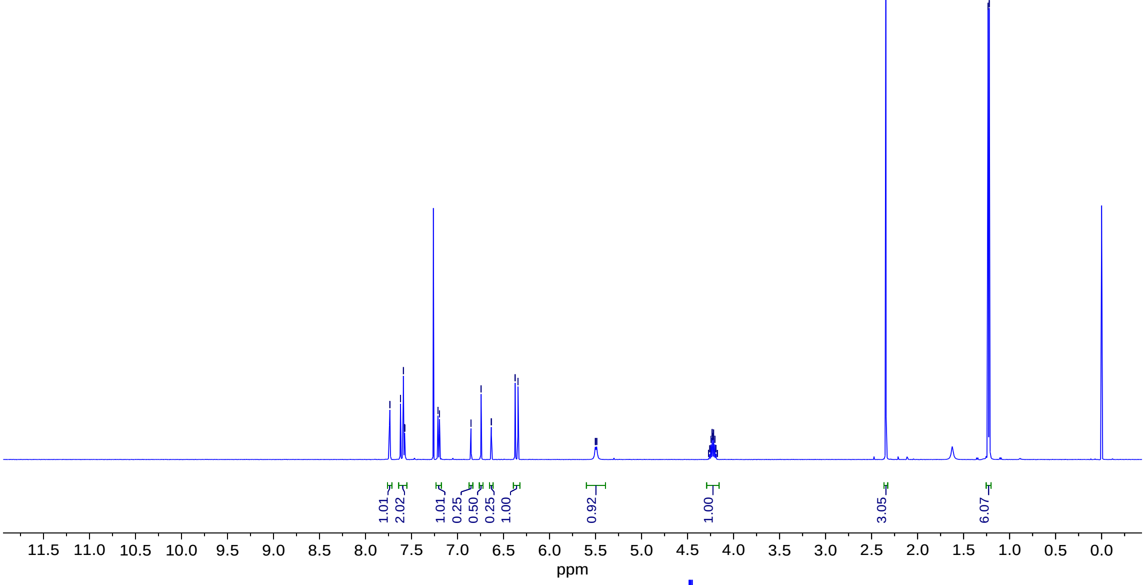
7.74 7.62 7.59 7.58 7.57 7.21 7.20 6.85 6.74 6.63 6.37 6.34 5.50 5.49 4.27 4.26 4.25 4.23 4.23 4.22 4.22 4.20 4.19 4.18 2.34 1.23 1.22
1.01 2.02 1.01 0.25 0.50 0.25 1.00 0.92 1.00 3.05 6.07
11.5 11.0 10.5 10.0 9.5 9.0 8.5 8.0 7.5 7.0 6.5 6.0 5.5 5.0 4.5 4.0 3.5 3.0 2.5 2.0 1.5 1.0 0.5 0.0
ppm

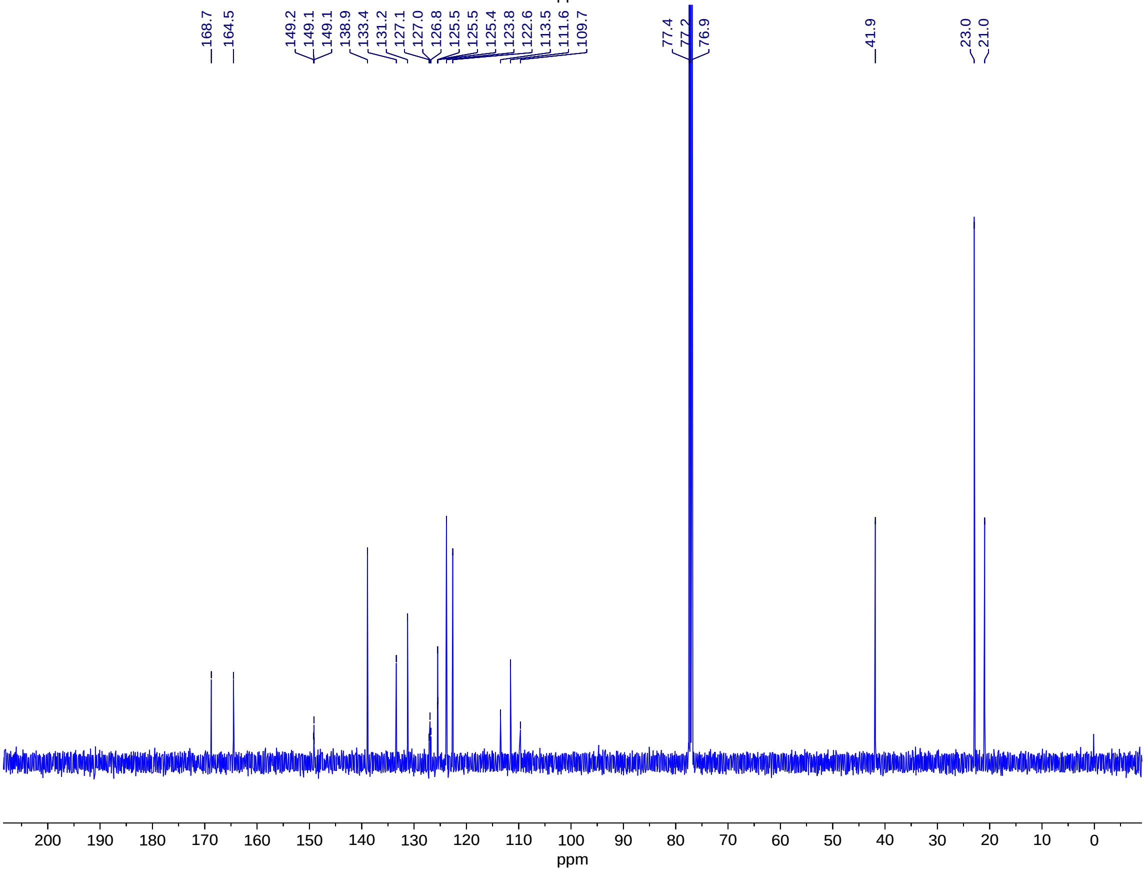
168.7 164.5 149.2 149.1 149.1 138.9 133.4 131.2 127.1 127.0 126.8 125.5 125.5 125.4 123.8 122.6 113.5 111.6 109.7 77.4 77.2 76.9 41.9 23.0 21.0
200 190 180 170 160 150 140 130 120 110 100 90 80 70 60 50 40 30 20 10 0
ppm

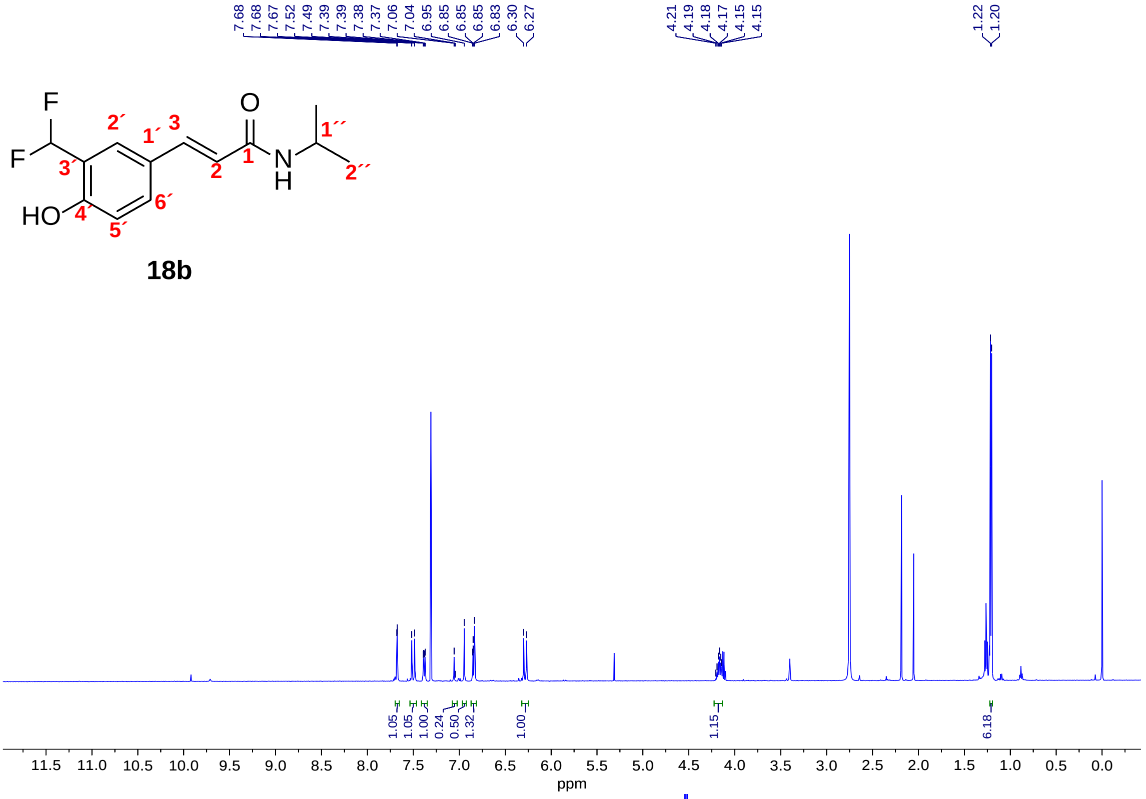
F
F
HO
O
N
H
1
2
3
1´
2´
3´
4´
5´
6´
1´´
2´´
18b
ppm

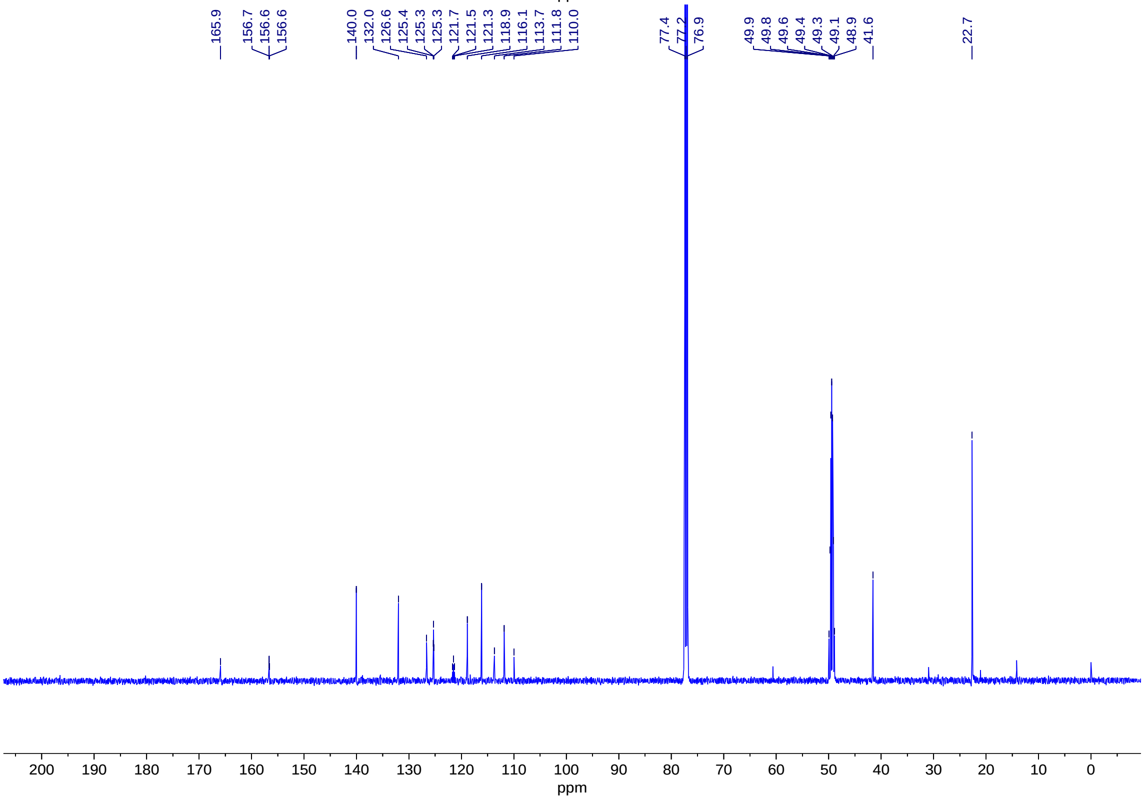
ppm

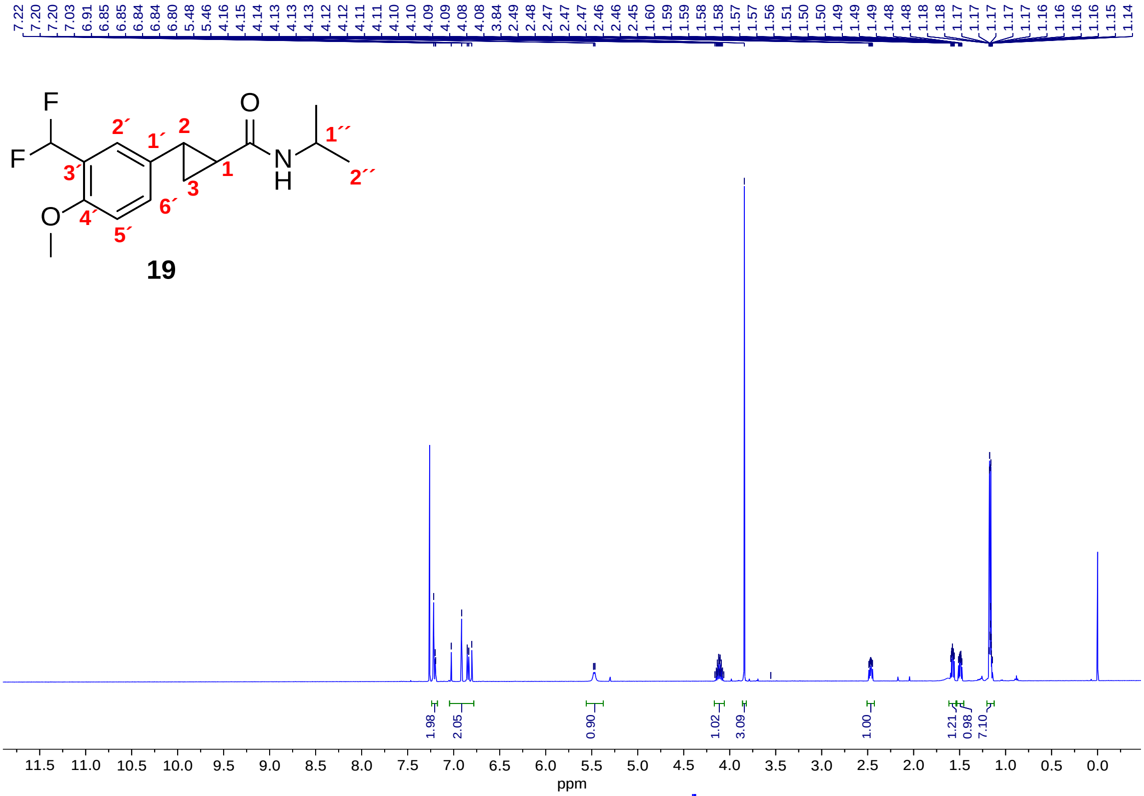
7.22 7.20 7.20 7.03 6.91 6.85 6.84 6.84 6.80 5.48 5.46 4.16 4.15 4.14 4.13 4.13 4.13 4.12 4.12 4.11 4.11 4.10 4.10 4.09 4.09 4.08 4.08 3.84 2.49 2.48 2.47 2.47 2.47 2.46 2.46 2.45 1.60 1.59 1.59 1.58 1.58 1.57 1.57 1.56 1.51 1.50 1.50 1.49 1.49 1.49 1.48 1.48 1.18 1.18 1.17 1.17 1.17 1.17 1.17 1.16 1.16 1.16 1.15 1.14
F
F
O
N
H
O
1´
2´
3´
4´
5´
6´
1
2
3
1´´
2´´
19
1.98
2.05
0.90
1.02
3.09
1.00
1.21
0.98
7.10
11.5 11.0 10.5 10.0 9.5 9.0 8.5 8.0 7.5 7.0 6.5 6.0 5.5 5.0 4.5 4.0 3.5 3.0 2.5 2.0 1.5 1.0 0.5 0.0
ppm

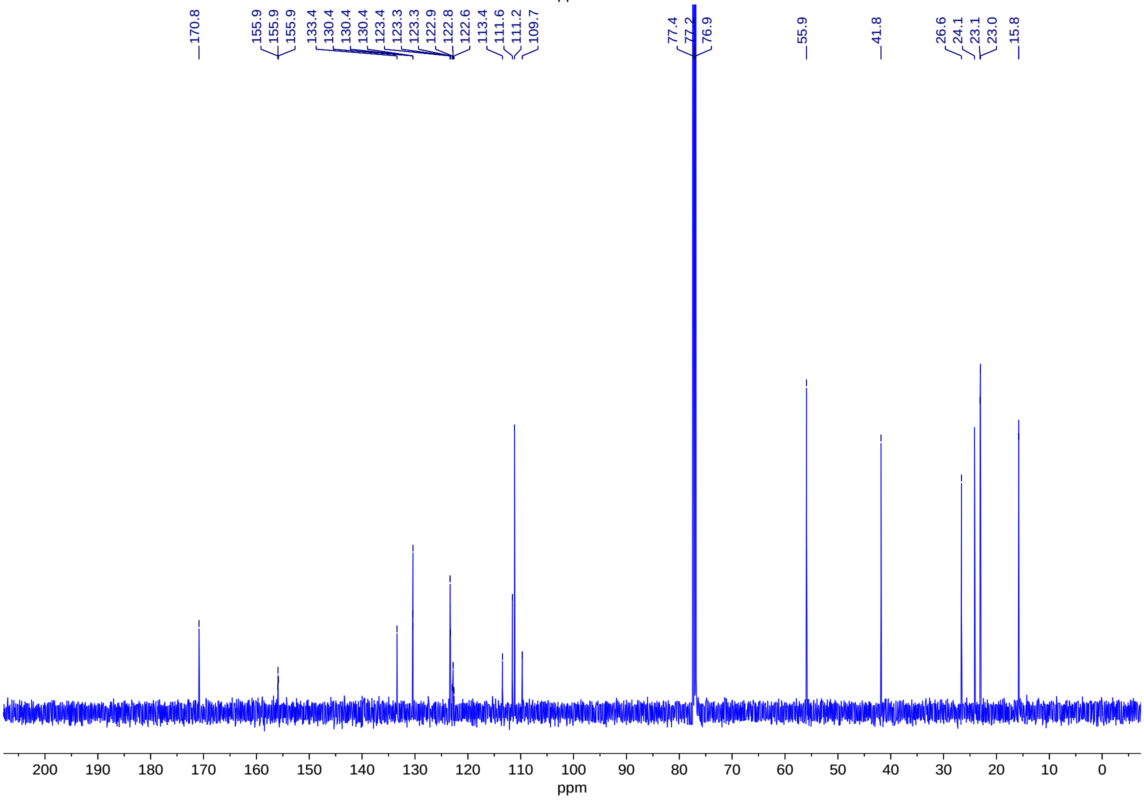
170.8
155.9 155.9 155.9 133.4 130.4 130.4 130.4 123.4 123.4 123.3 123.3 122.9 122.8 122.6 113.4 111.6 111.2 109.7
77.4 77.2 76.9
55.9
41.8
26.6 24.1 23.1 23.0 15.8
200 190 180 170 160 150 140 130 120 110 100 90 80 70 60 50 40 30 20 10 0
ppm

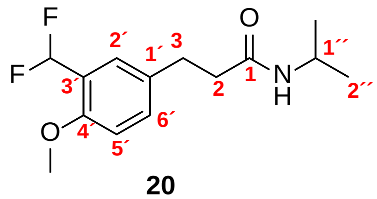

20

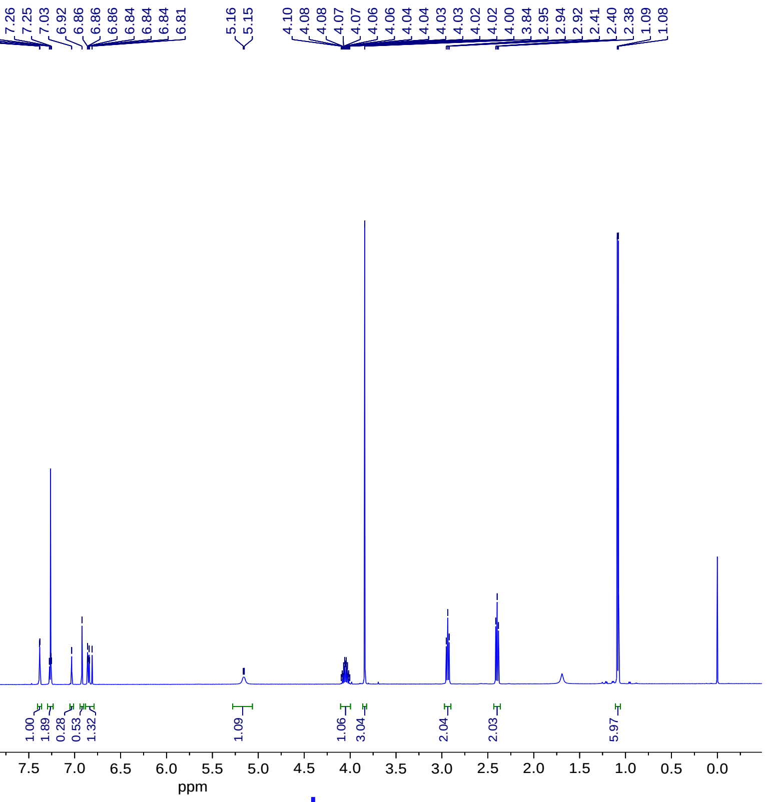

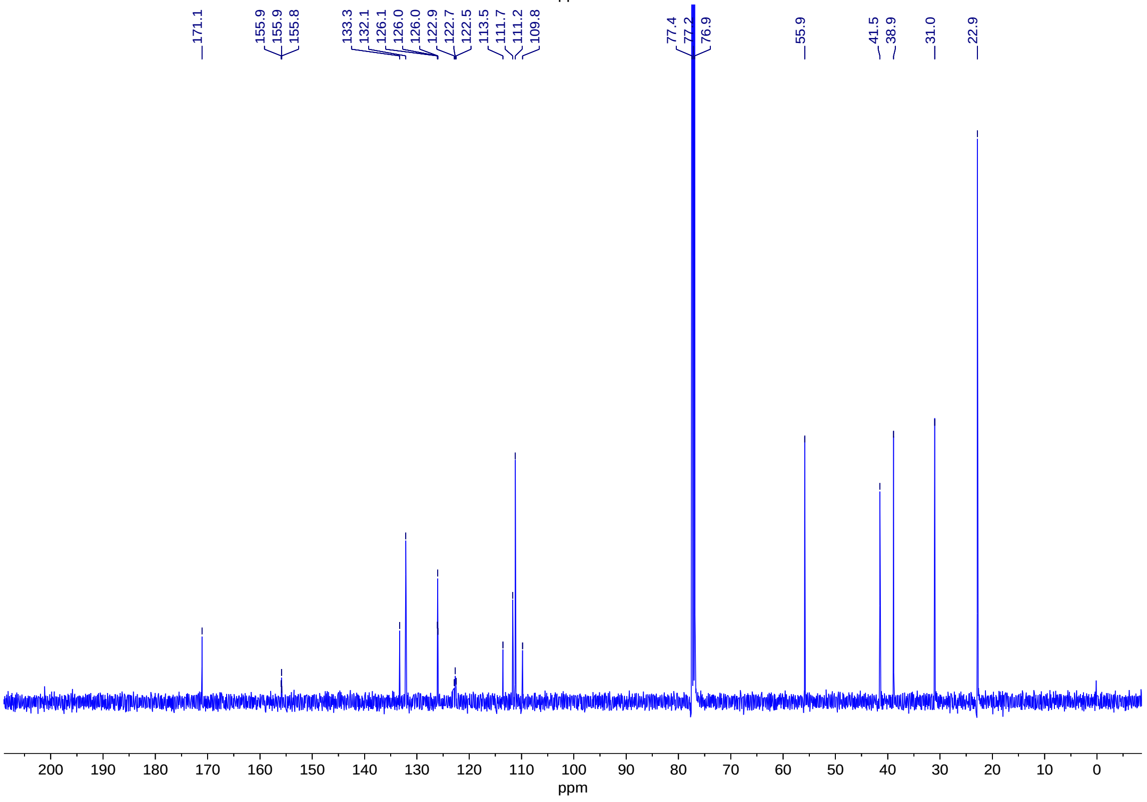

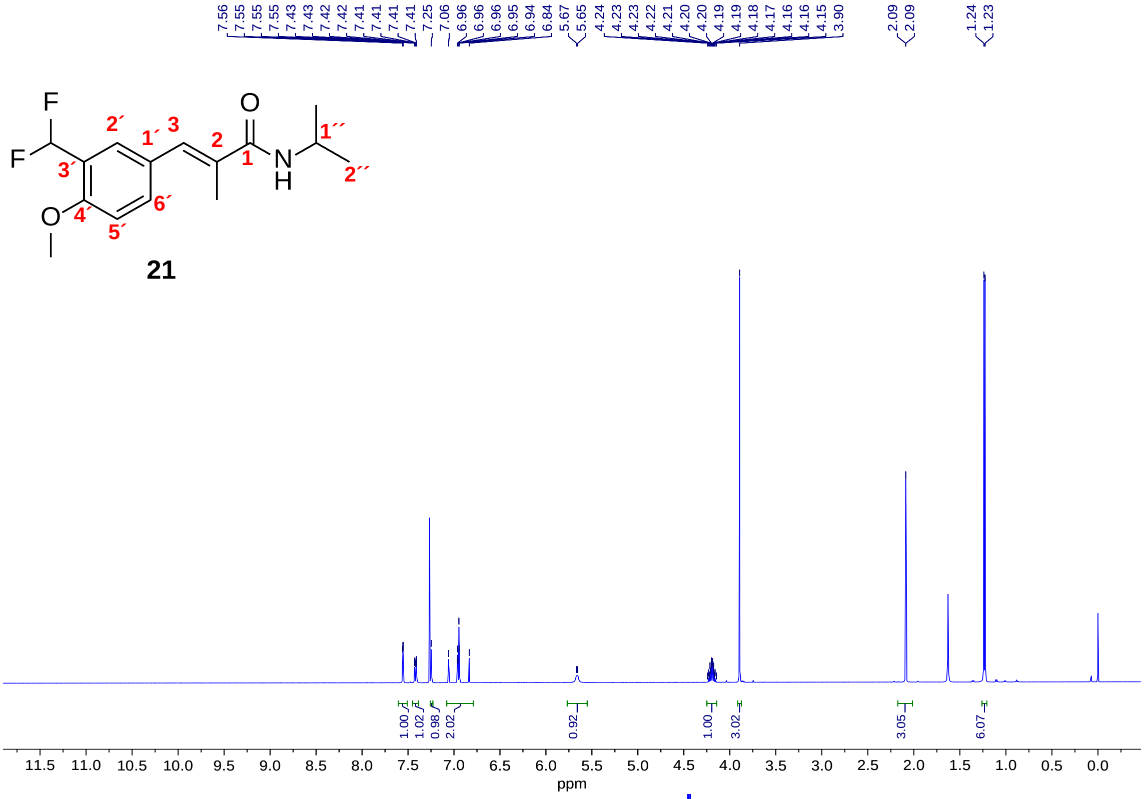
F
F
O
N
H
O
1
2
3
1´
2´
3´
4´
5´
6´
1´´
2´´

21

7.56
7.55
7.55
7.55
7.43
7.43
7.42
7.42
7.41
7.41
7.41
7.41
7.25
7.06
6.96
6.96
6.96
6.95
6.94
6.84
5.67
5.65
4.24
4.23
4.23
4.22
4.21
4.20
4.20
4.19
4.19
4.18
4.17
4.16
4.16
4.15
3.90
2.09
2.09
1.24
1.23
1.00
1.02
0.98
2.02
0.92
1.00
3.02
3.05
6.07
11.5 11.0 10.5 10.0 9.5 9.0 8.5 8.0 7.5 7.0 6.5 6.0 5.5 5.0 4.5 4.0 3.5 3.0 2.5 2.0 1.5 1.0 0.5 0.0
ppm

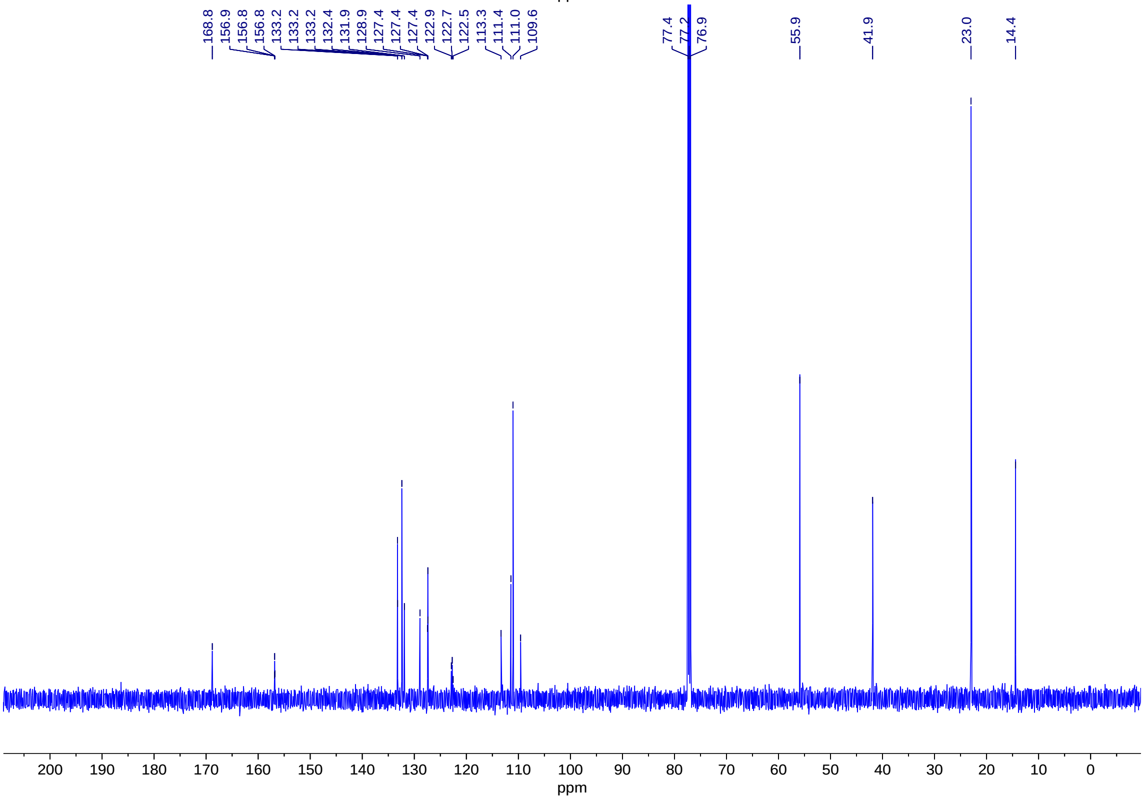
168.8
156.9
156.8
156.8
133.2
133.2
133.2
132.4
131.9
128.9
127.4
127.4
127.4
122.9
122.7
122.5
113.3
111.4
111.0
109.6
77.4
77.2
76.9
55.9
41.9
23.0
14.4
200 190 180 170 160 150 140 130 120 110 100 90 80 70 60 50 40 30 20 10 0
ppm

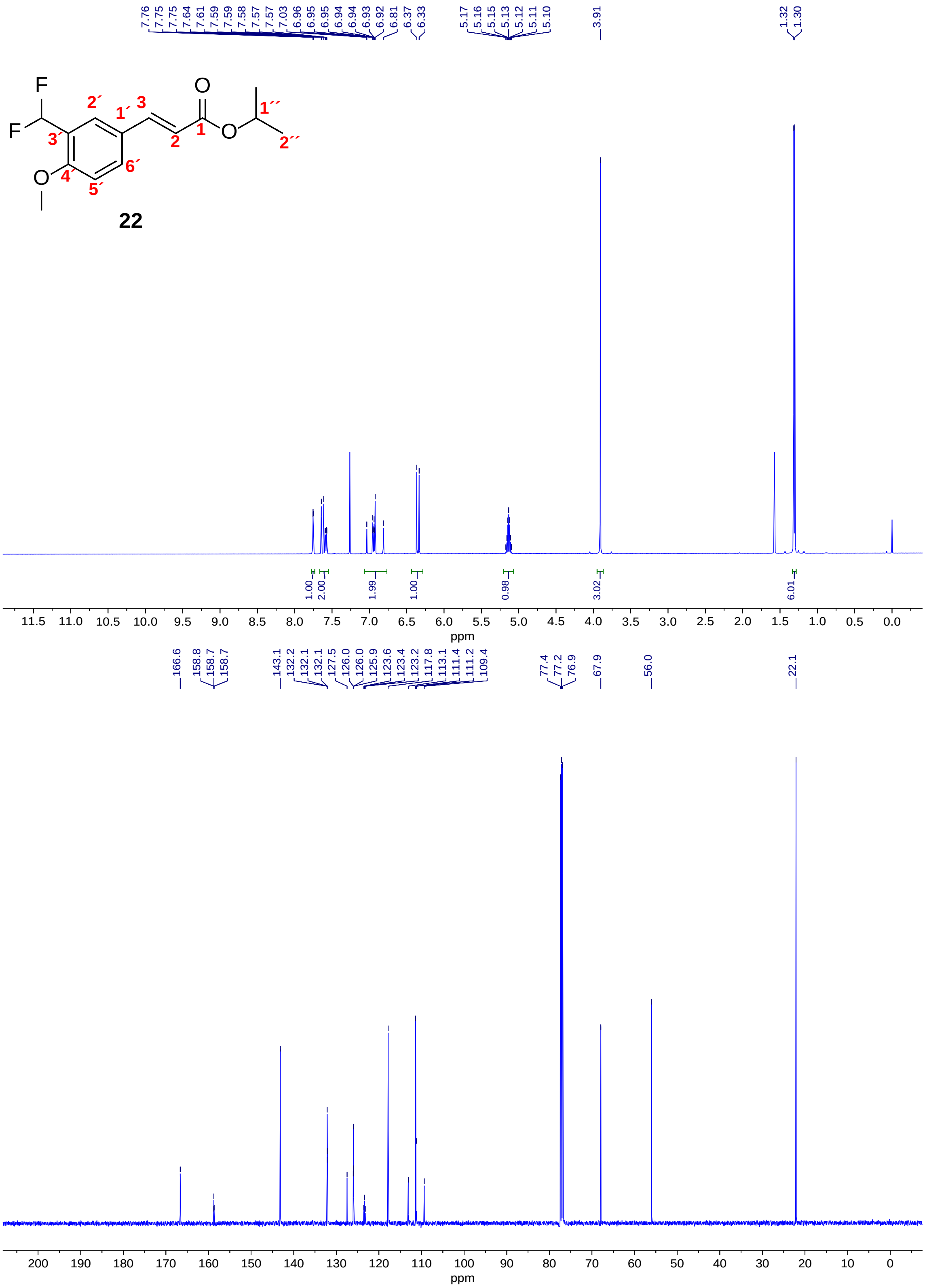
22
ppm
ppm

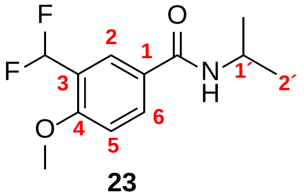
F
F
O
N
H
O
1
2
3
4
5
6
1´
2´

23

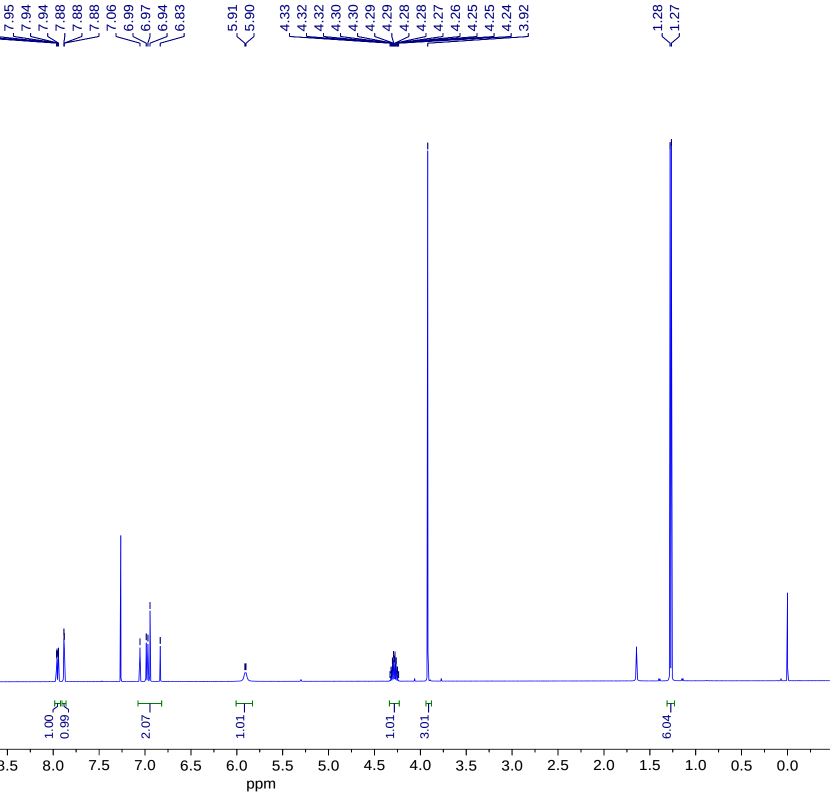
7.96
7.96
7.96
7.95
7.94
7.94
7.88
7.88
7.88
7.06
6.99
6.97
6.94
6.83
5.91
5.90
4.33
4.32
4.32
4.30
4.30
4.29
4.29
4.28
4.28
4.27
4.26
4.25
4.25
4.24
3.92
1.28
1.27
1.00
0.99
2.07
1.01
1.01
3.01
6.04
11.5 11.0 10.5 10.0 9.5 9.0 8.5 8.0 7.5 7.0 6.5 6.0 5.5 5.0 4.5 4.0 3.5 3.0 2.5 2.0 1.5 1.0 0.5 0.0
ppm

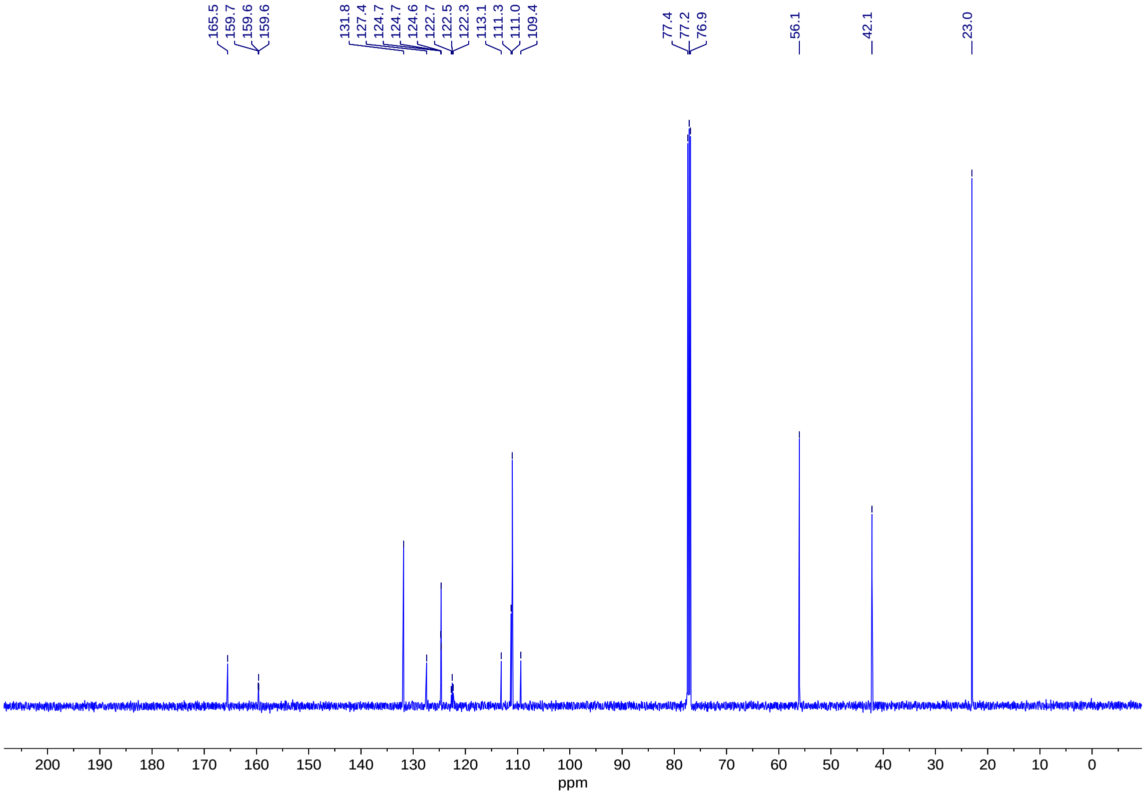
165.5
159.7
159.6
159.6
131.8
127.4
124.7
124.7
124.6
122.7
122.5
122.3
113.1
111.3
111.0
109.4
77.4
77.2
76.9
56.1
42.1
23.0
200 190 180 170 160 150 140 130 120 110 100 90 80 70 60 50 40 30 20 10 0
ppm

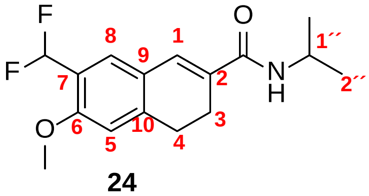
F
F
O
N
H
O
1
2
3
4
5
6
7
8
9
10
1´´
2´´

24

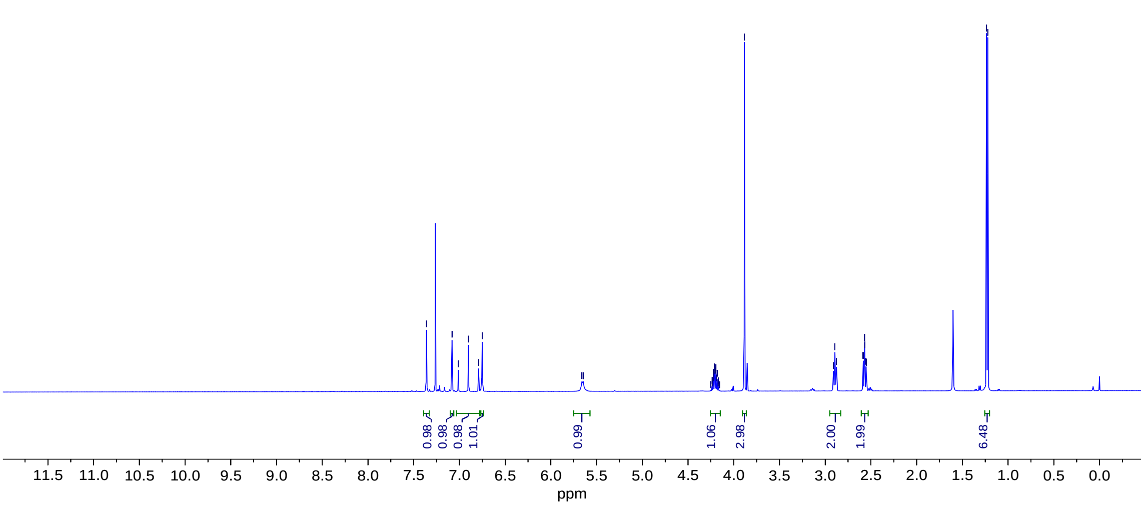
7.36
7.08
7.01
6.90
6.79
6.75
5.66
5.64
4.25
4.24
4.23
4.22
4.22
4.21
4.21
4.20
4.19
4.18
4.18
4.17
4.17
4.16
3.88
2.91
2.89
2.88
2.59
2.58
2.57
2.57
2.55
2.55
1.24
1.22
0.98
0.98
0.98
1.01
0.99
1.06
2.98
2.00
1.99
6.48
ppm

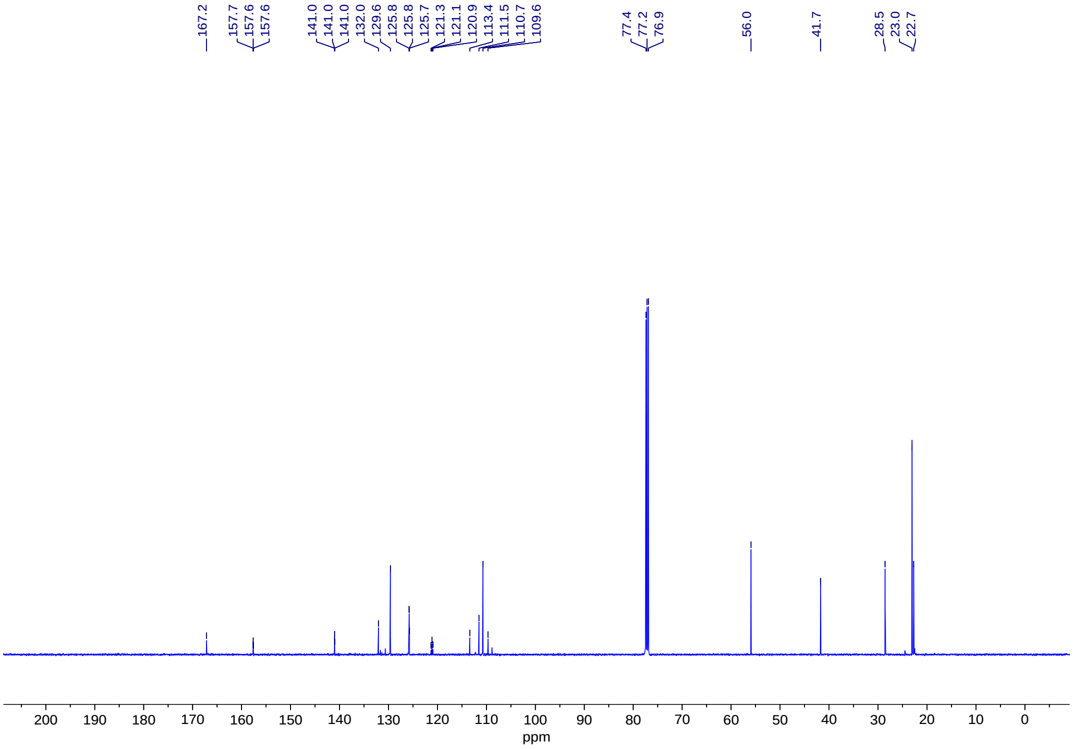
167.2
157.7
157.6
157.6
141.0
141.0
141.0
132.0
129.6
125.8
125.8
125.7
121.3
121.1
120.9
113.4
111.5
110.7
109.6
77.4
77.2
76.9
56.0
41.7
28.5
23.0
22.7
ppm

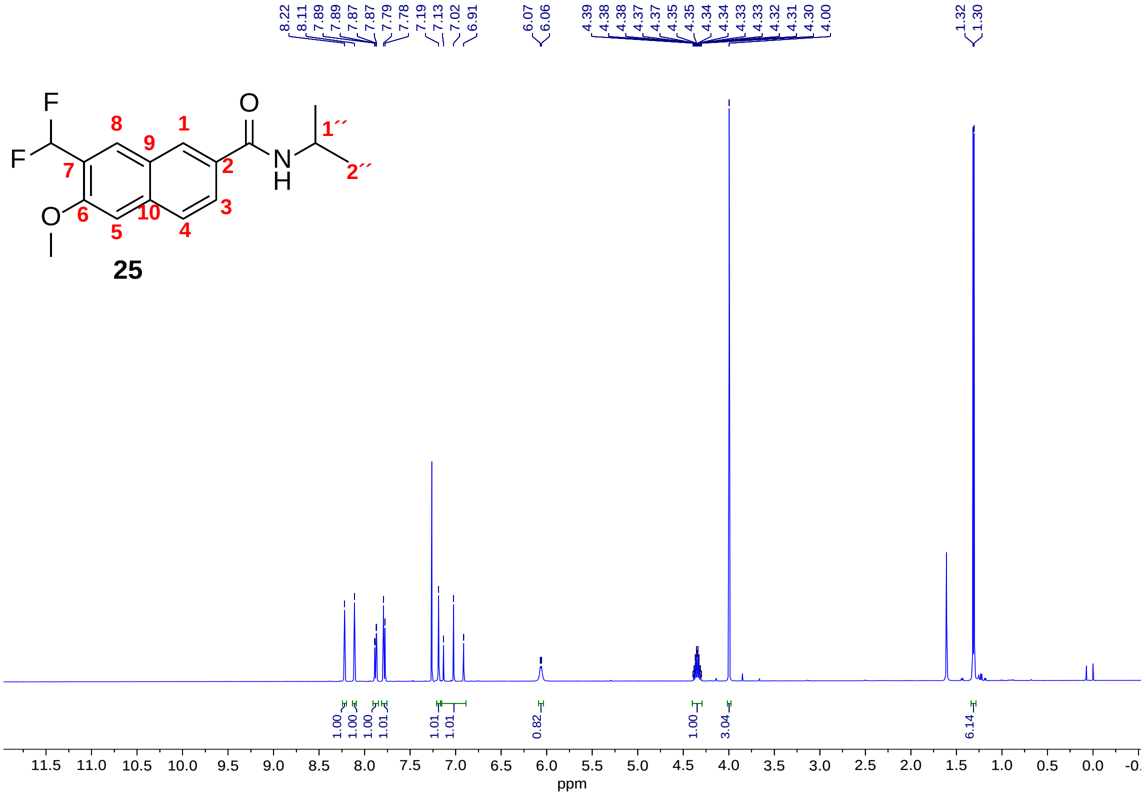

F
F
O
N
H
1
2
3
4
5
6
7
8
9
10
1´´
2´´

25

8.22
8.11
7.89
7.89
7.87
7.87
7.79
7.78
7.19
7.13
7.02
6.91
6.07
6.06
4.39
4.38
4.38
4.37
4.37
4.35
4.35
4.34
4.34
4.33
4.33
4.32
4.31
4.30
4.00
1.32
1.30
1.00
1.00
1.00
1.01
1.01
1.01
0.82
1.00
3.04
6.14
11.5 11.0 10.5 10.0 9.5 9.0 8.5 8.0 7.5 7.0 6.5 6.0 5.5 5.0 4.5 4.0 3.5 3.0 2.5 2.0 1.5 1.0 0.5 0.0
ppm

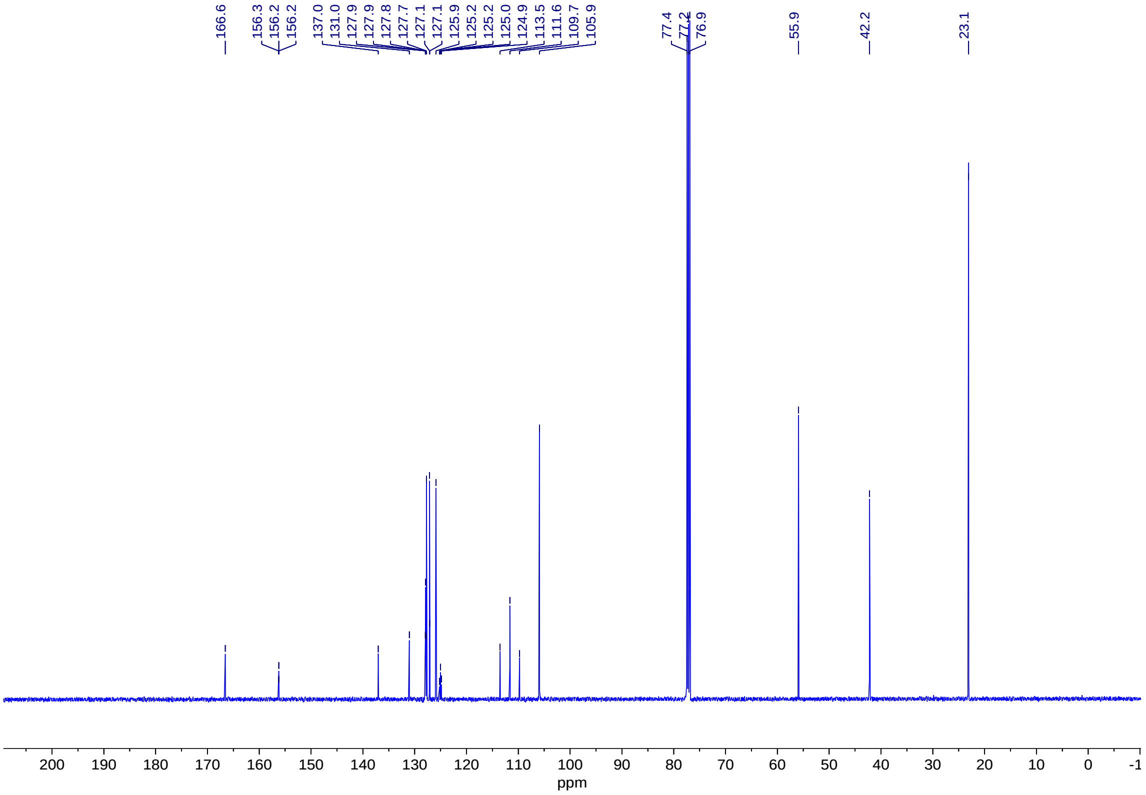

166.6
156.3
156.2
156.2
137.0
131.0
127.9
127.9
127.8
127.7
127.1
127.1
125.9
125.2
125.2
125.0
124.9
113.5
111.6
109.7
105.9
77.4
77.2
76.9
55.9
42.2
23.1
200 190 180 170 160 150 140 130 120 110 100 90 80 70 60 50 40 30 20 10 0 -1
ppm

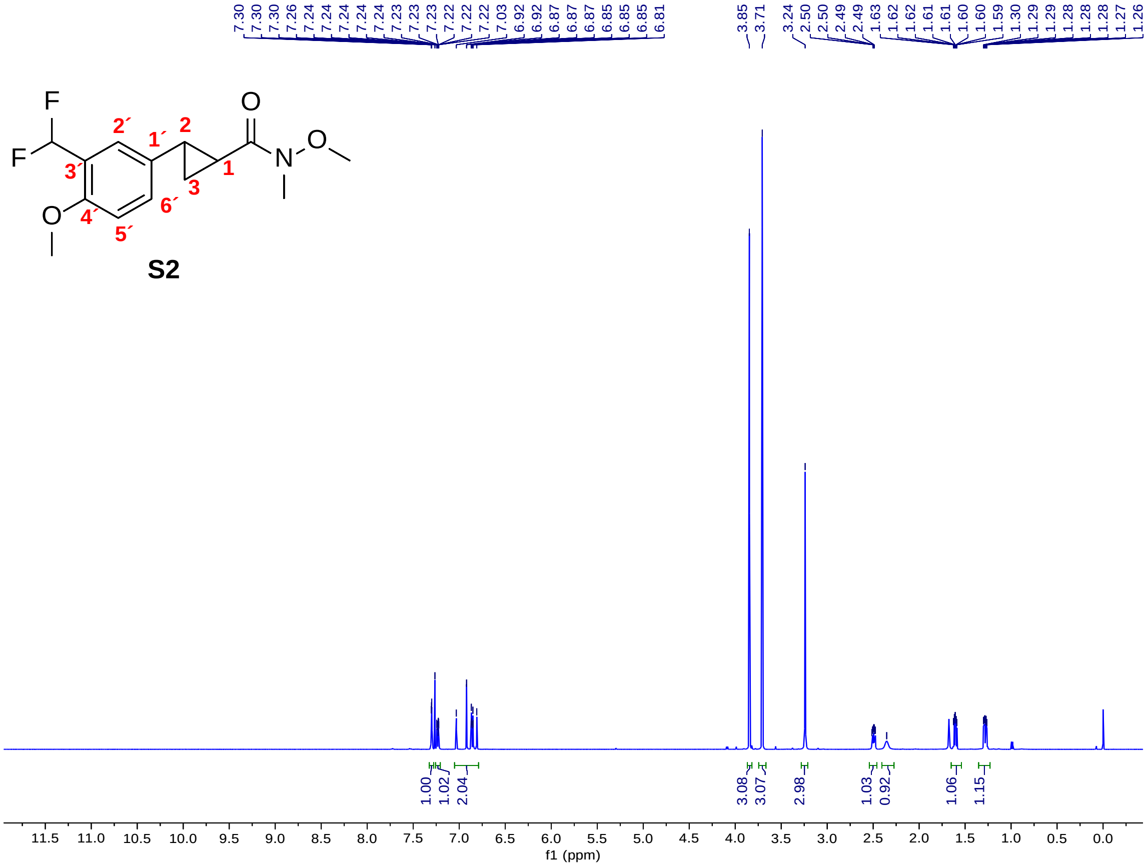

S2

7.30 7.30 7.30 7.26 7.24 7.24 7.24 7.24 7.24 7.23 7.23 7.23 7.22 7.22 7.22 7.03 6.92 6.92 6.87 6.87 6.85 6.85 6.85 6.81
3.85 3.71 3.24 2.50 2.50 2.49 2.49 1.63 1.62 1.61 1.61 1.60 1.60 1.59 1.30 1.29 1.29 1.28 1.28 1.28 1.27 1.26
1.00 1.02 2.04 3.08 3.07 2.98 1.03 0.92 1.06 1.15
f1 (ppm)

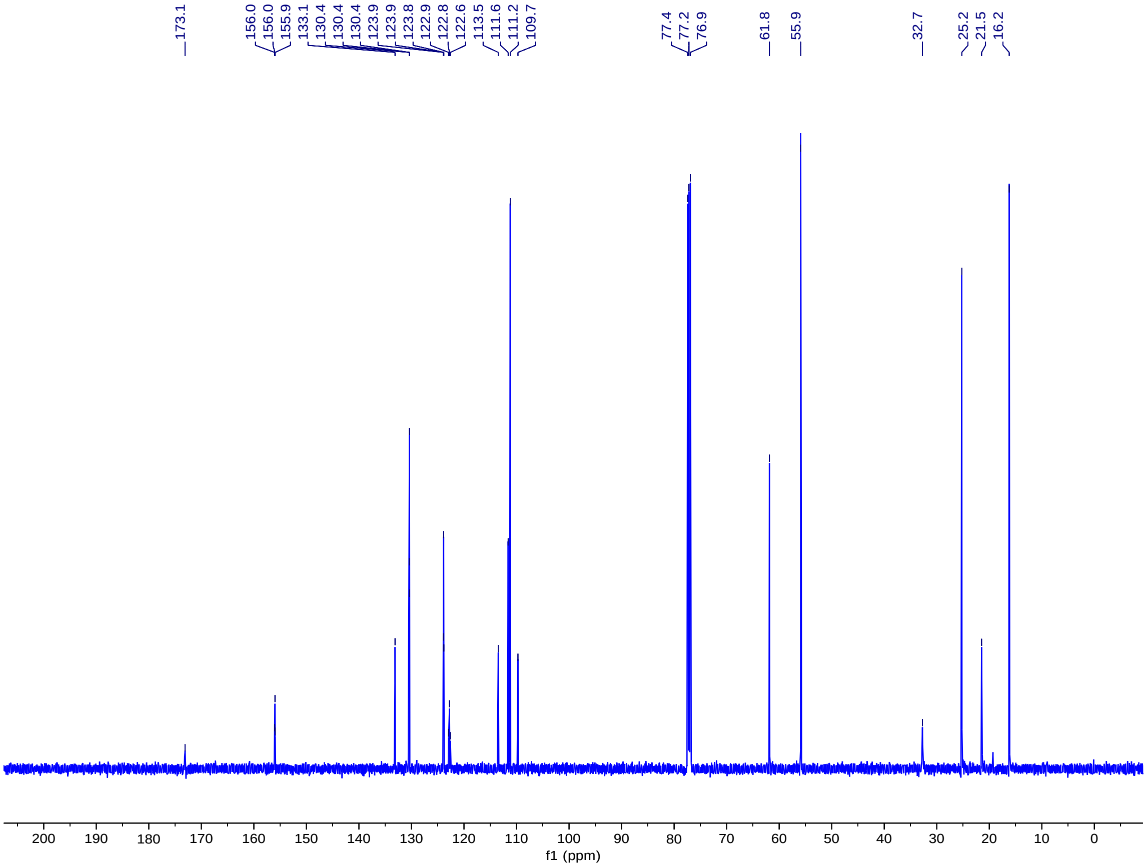
173.1 156.0 156.0 155.9 133.1 130.4 130.4 130.4 123.9 123.9 123.8 122.9 122.8 122.6 113.5 111.6 111.2 109.7 77.4 77.2 76.9 61.8 55.9 32.7 25.2 21.5 16.2
f1 (ppm)

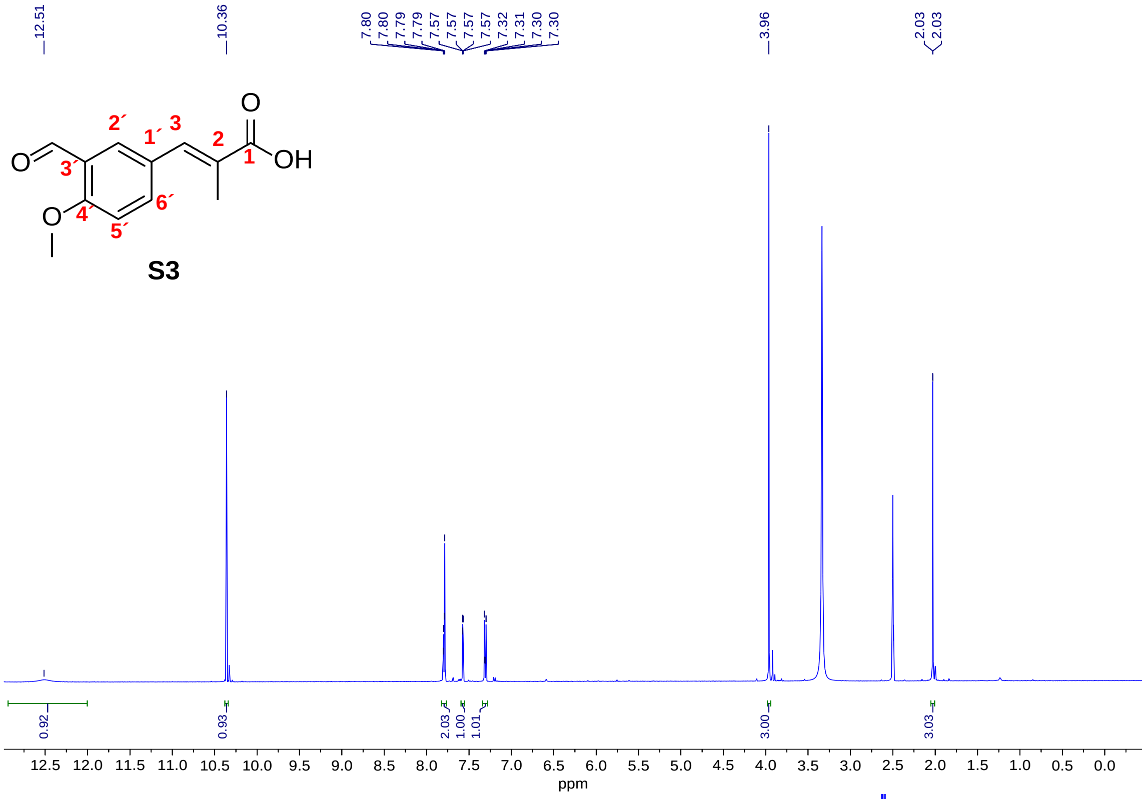

12.51
10.36
7.80 7.80 7.79 7.79 7.57 7.57 7.57 7.57 7.32 7.31 7.30 7.30
3.96
2.03 2.03
O
OH
O
O
1 2 3 1´ 2´ 3´ 4´ 5´ 6´
S3
0.92 0.93 2.03 1.00 1.01 3.00 3.03
ppm

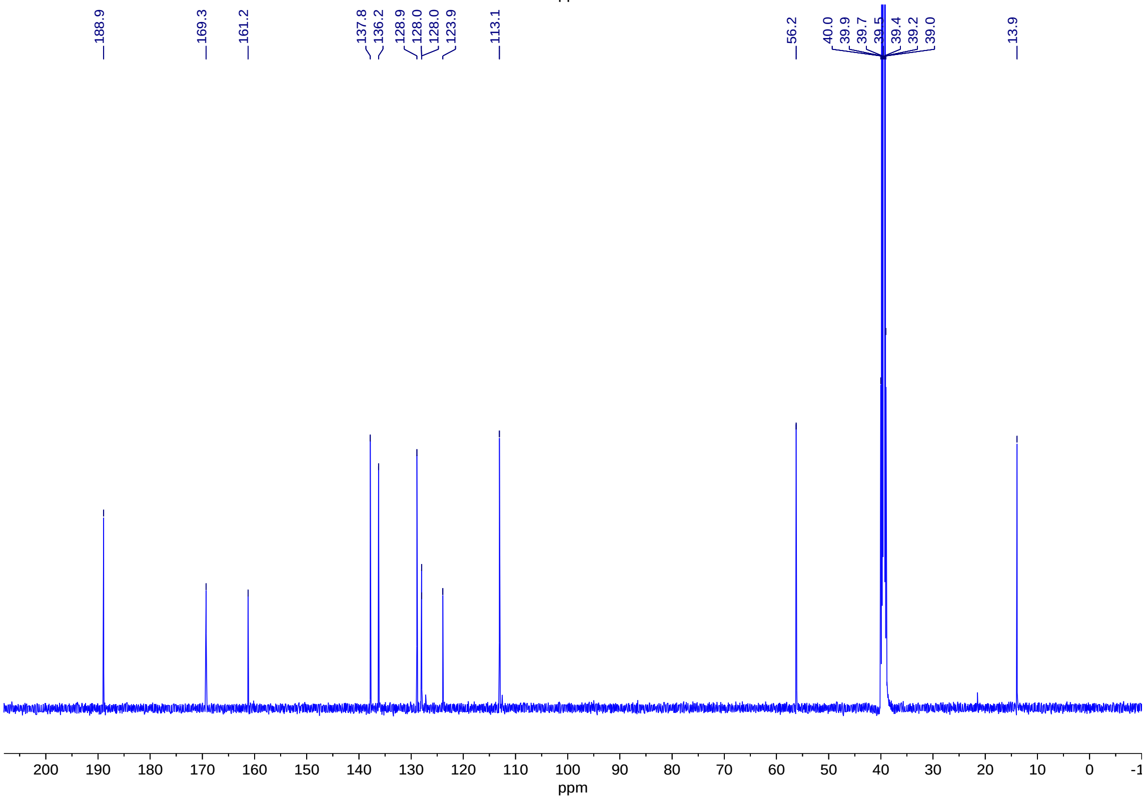

188.9
169.3
161.2
137.8 136.2 128.9 128.0 128.0 123.9
113.1
56.2
40.0 39.9 39.7 39.5 39.4 39.2 39.0
13.9
ppm

4. MS spectra

Compound **9**

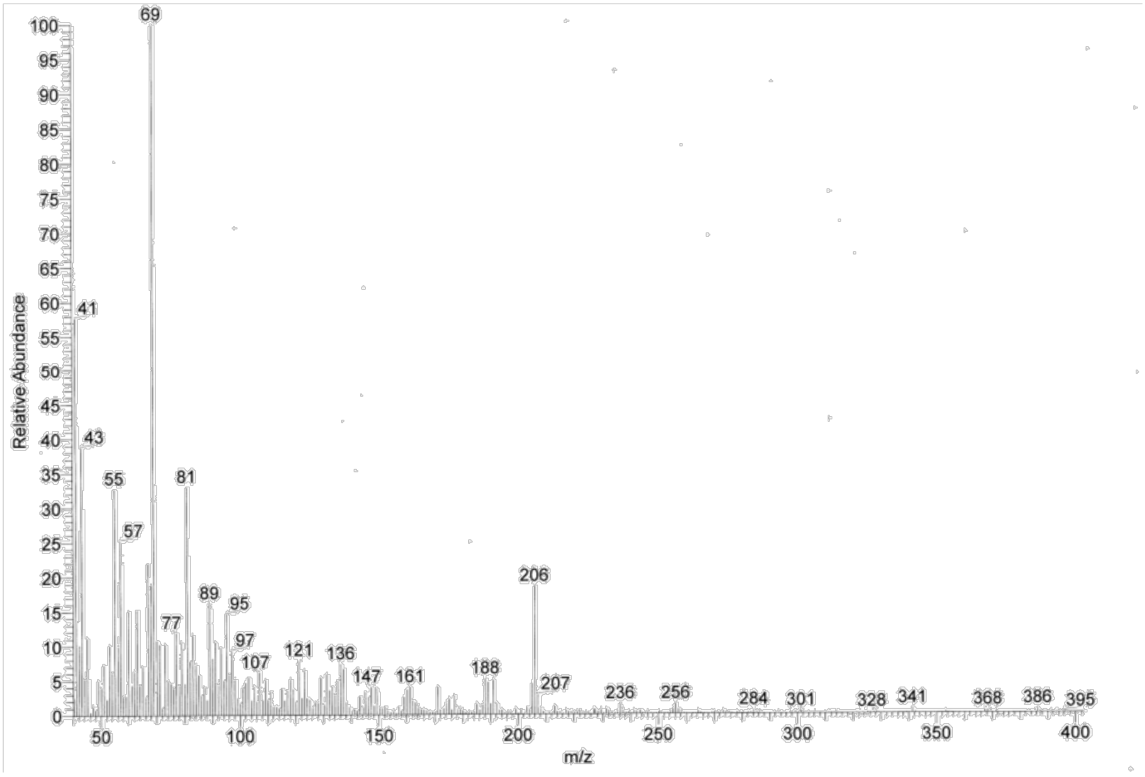

Compound **11a**

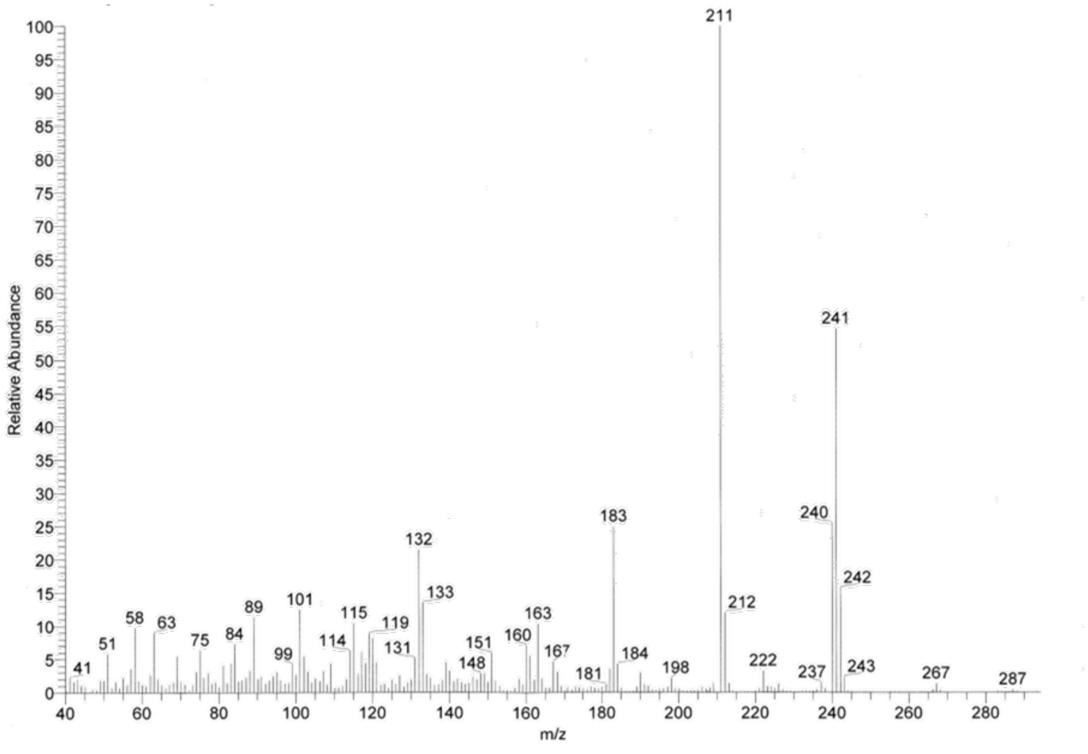

Compound **11b**

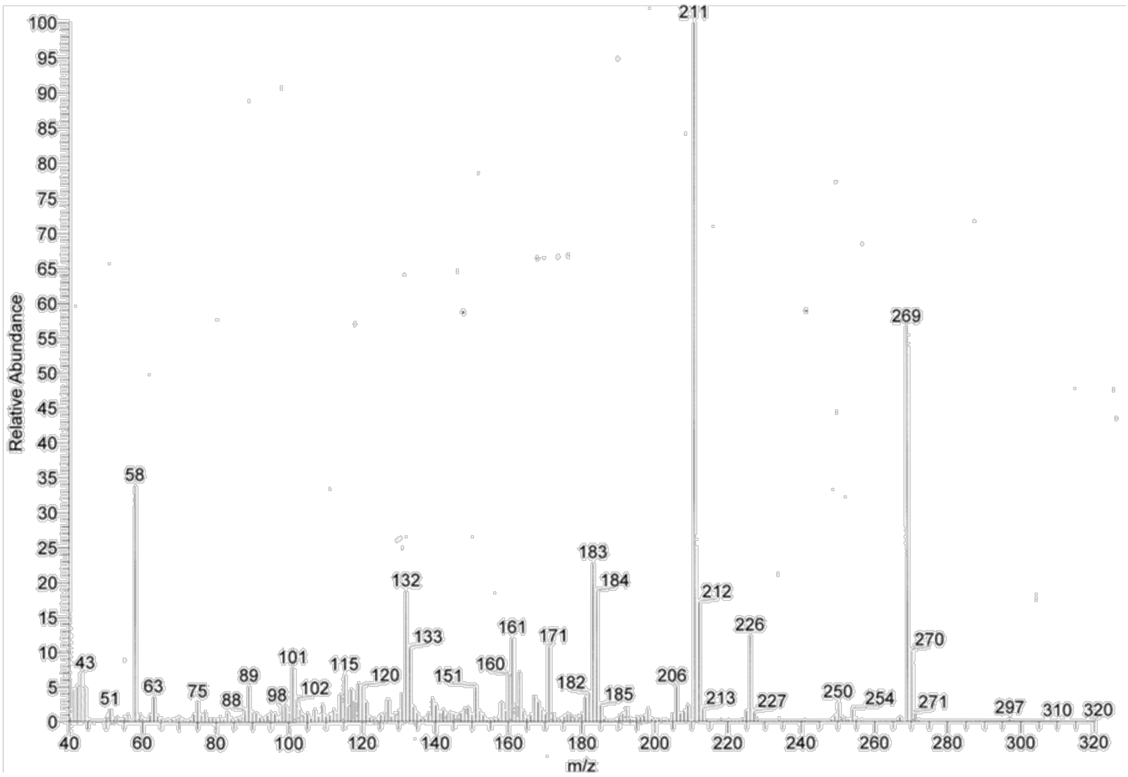

Compound **11c**

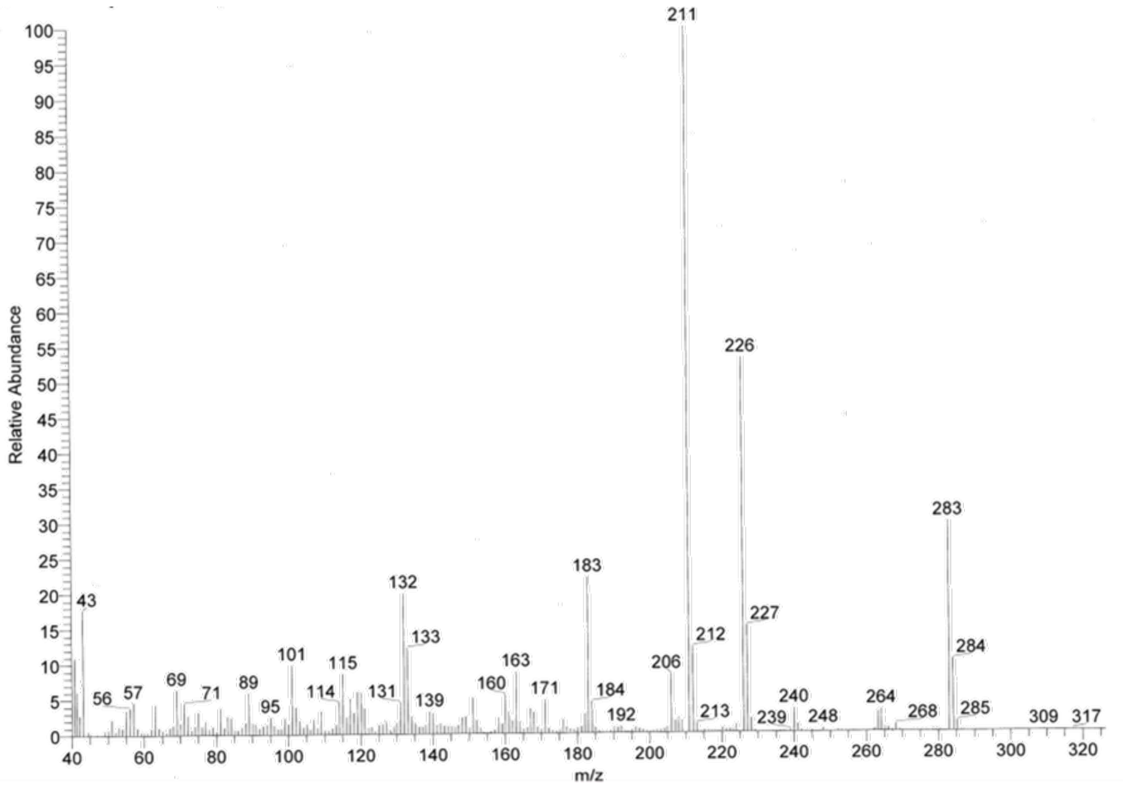

Compound **11d**

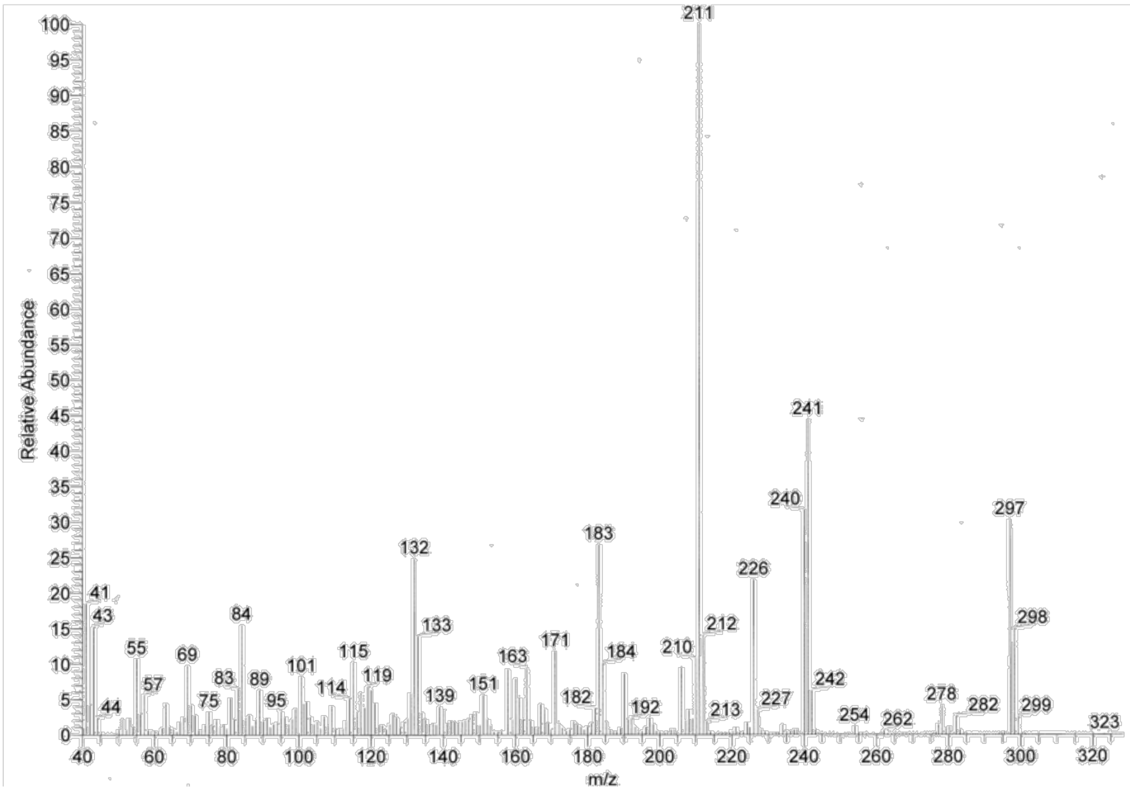

Compound **11e**

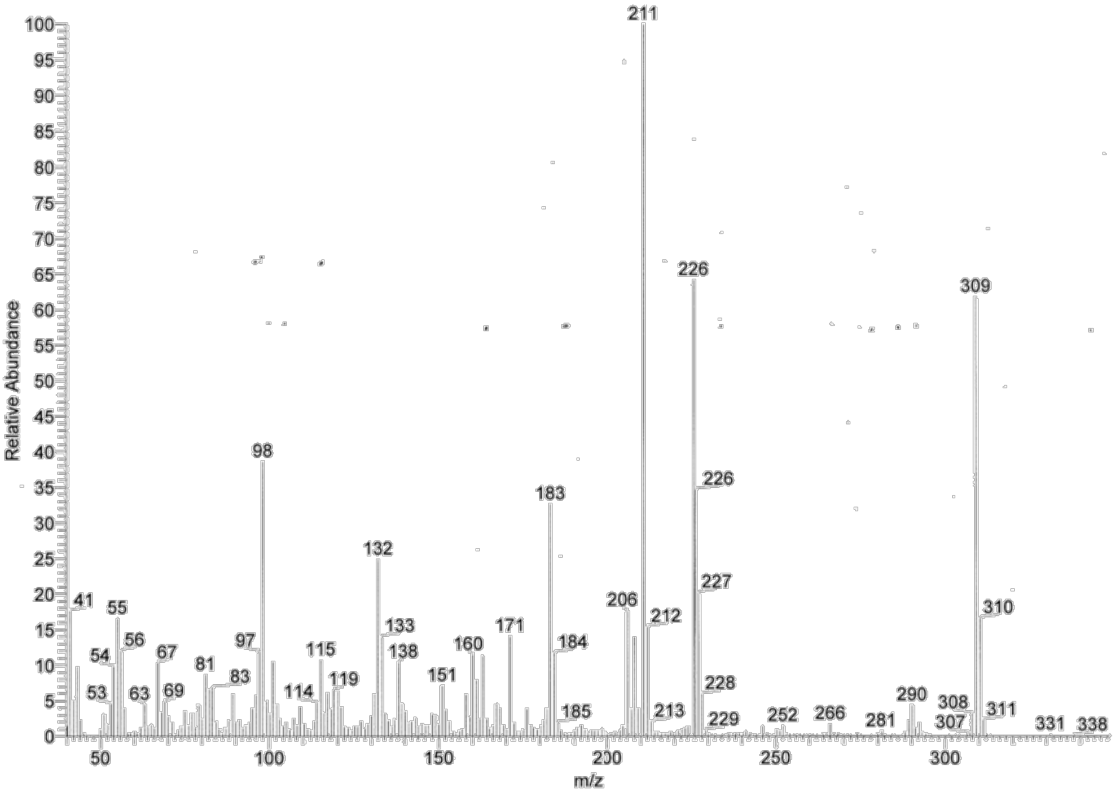

Compound **11f**

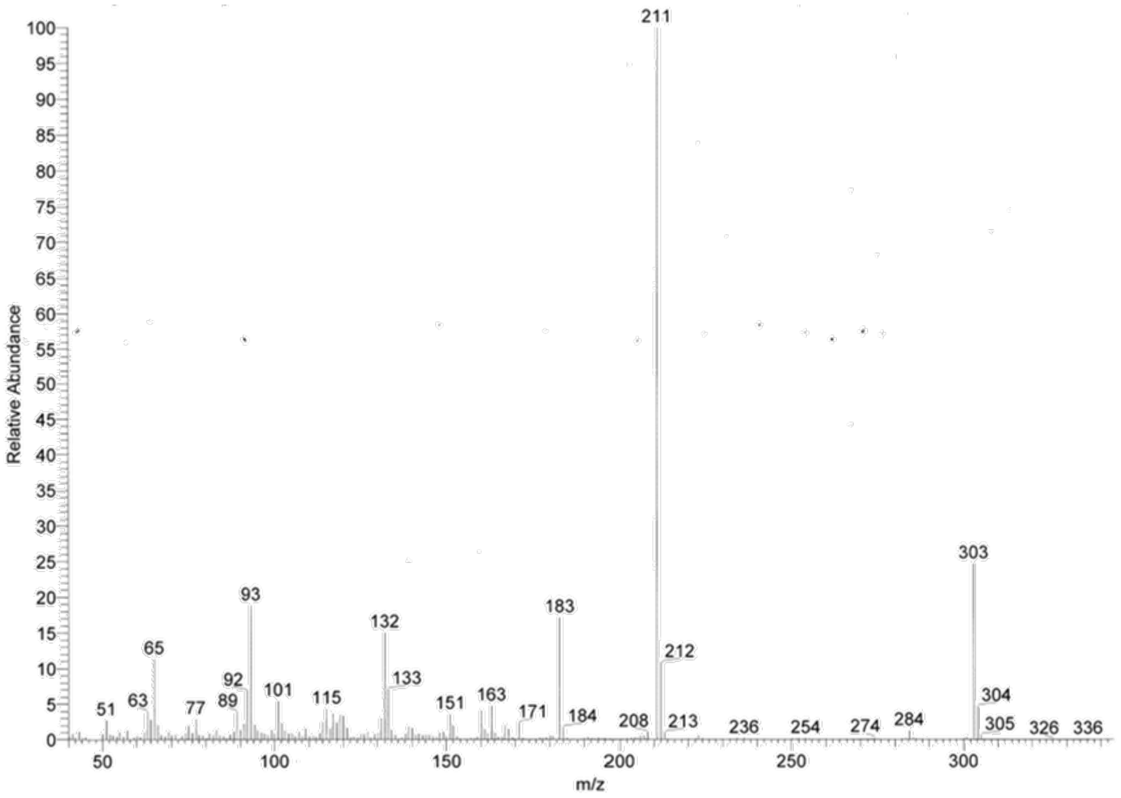

Compound **11g**

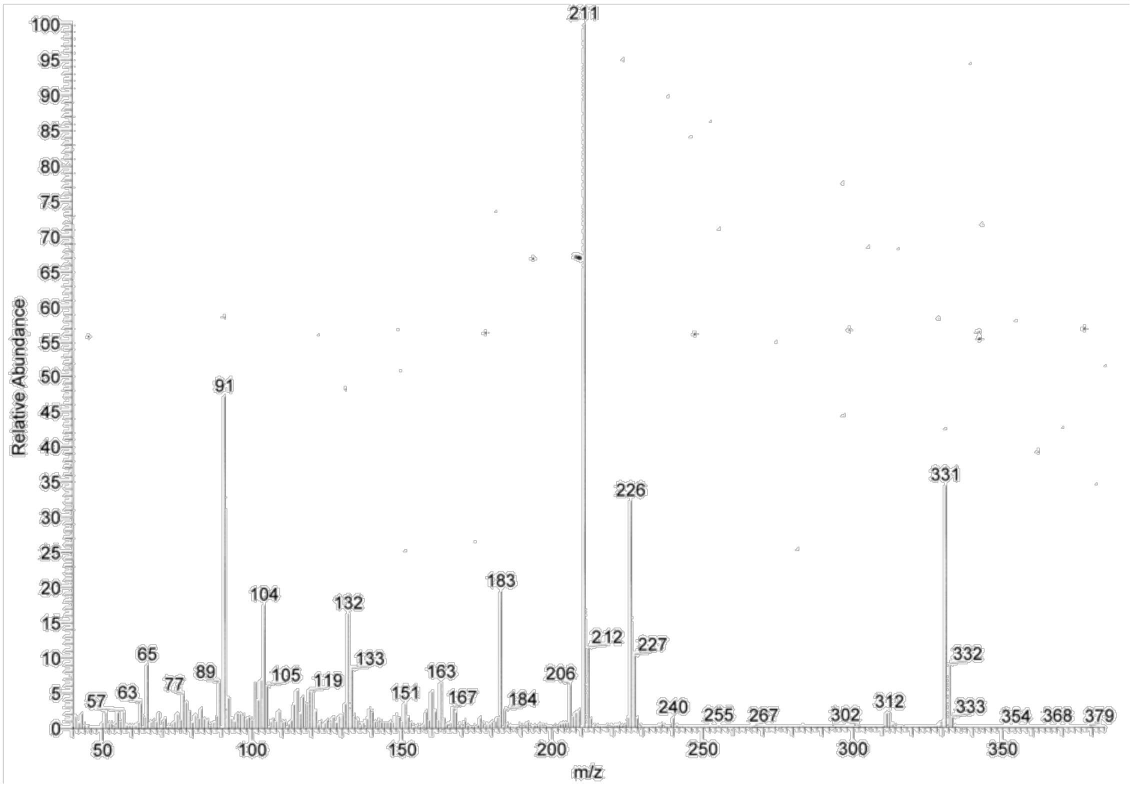

Compound **11h**

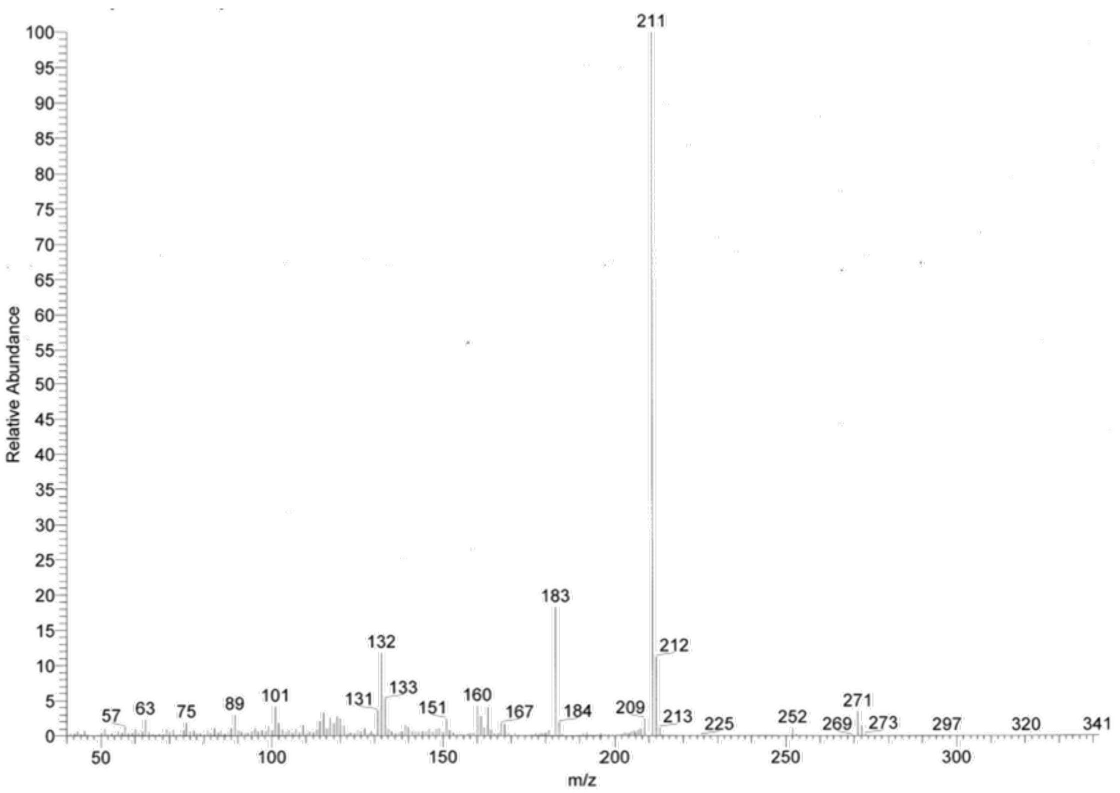

Compound **11i**

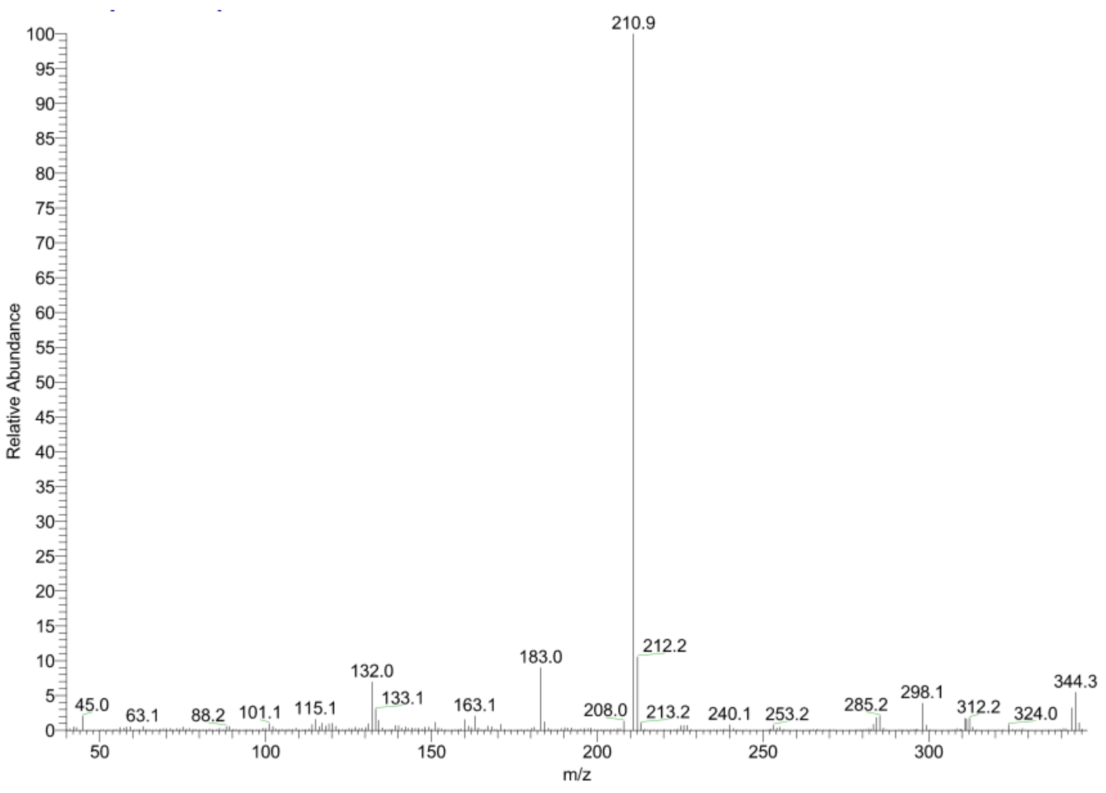

Compound **11j**

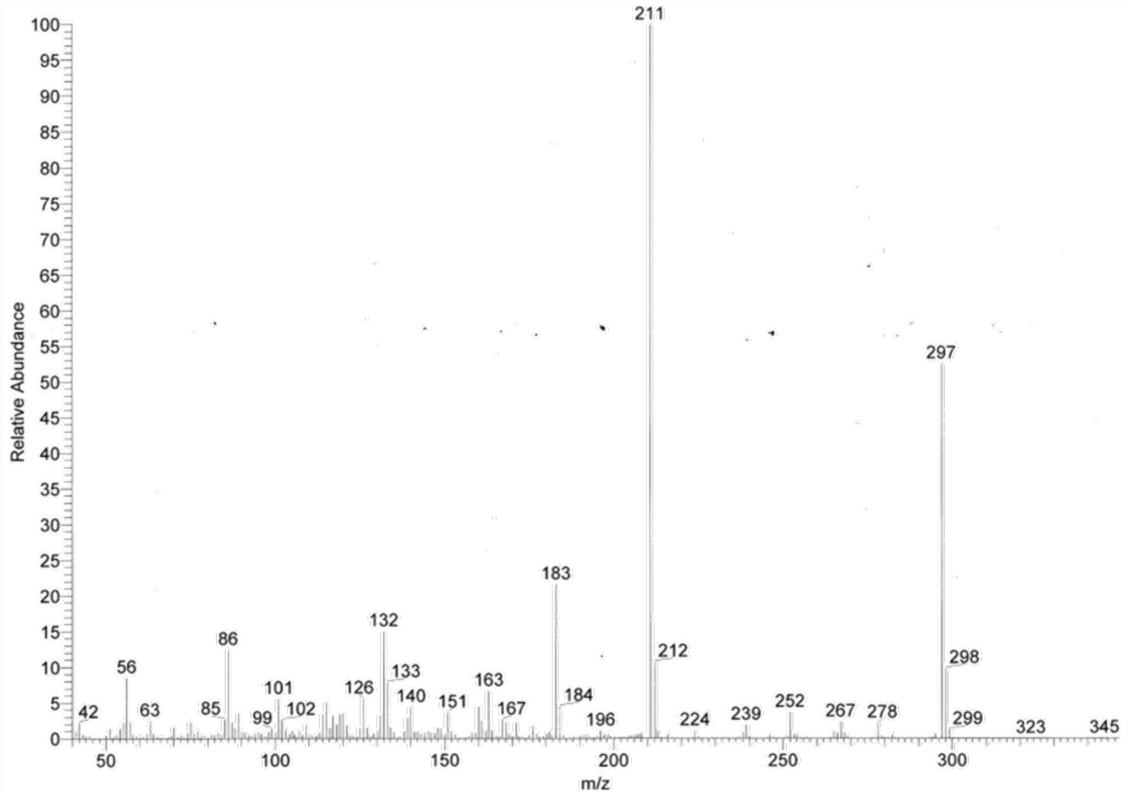

Compound **11k**

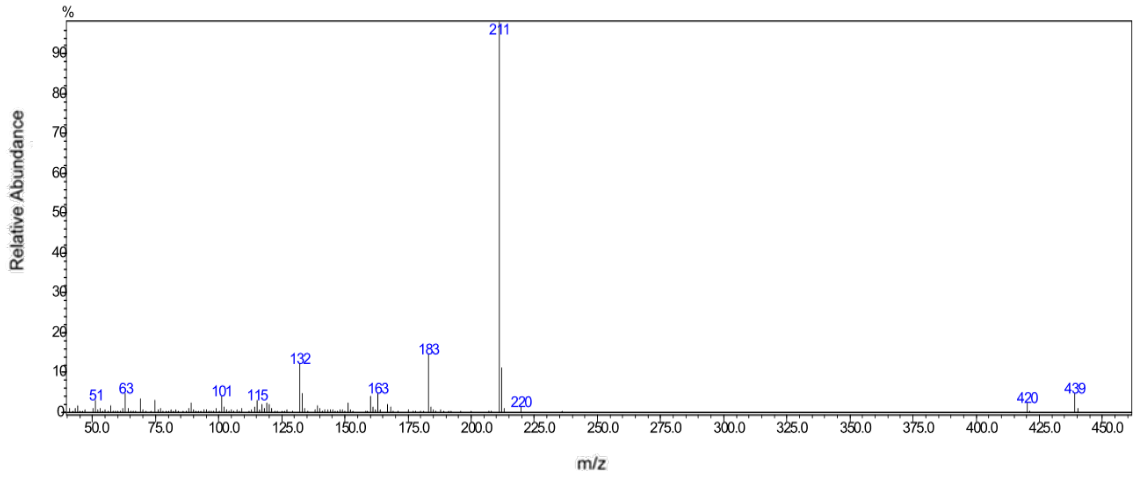

Compound **11l**

Acquisition Parameter

Source Type	ESI	Ion Polarity	Positive	Set Nebulizer	0.4 Bar
Focus	Not active	Set Capillary	4500 V	Set Dry Heater	180 °C
Scan Begin	50 m/z	Set End Plate Offset	-500 V	Set Dry Gas	4.0 l/min
Scan End	950 m/z	Set Collision Cell RF	150.0 Vpp	Set Divert Valve	Source

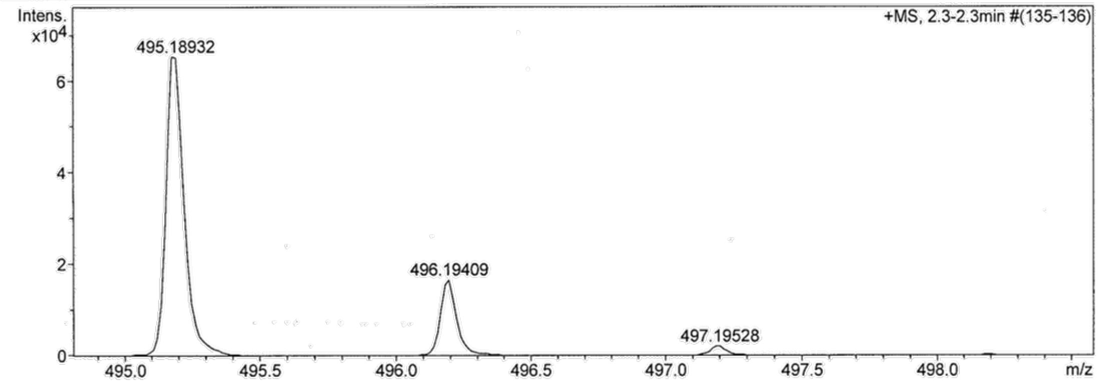

Meas. m/z	#	Formula	m/z	err [ppm]	Mean err [ppm]	rdb	N-Rule	e⁻ Conf	mSigma
495.18932	1	C 25 H 27 F 4 N 2 O 4	495.19015	1.7	1.1	11.5	ok	even	15.6

Acquisition Parameter

Source Type	ESI	Ion Polarity	Positive	Set Nebulizer	0.4 Bar
Focus	Not active	Set Capillary	4500 V	Set Dry Heater	180 °C
Scan Begin	50 m/z	Set End Plate Offset	-500 V	Set Dry Gas	4.0 l/min
Scan End	950 m/z	Set Collision Cell RF	150.0 Vpp	Set Divert Valve	Source

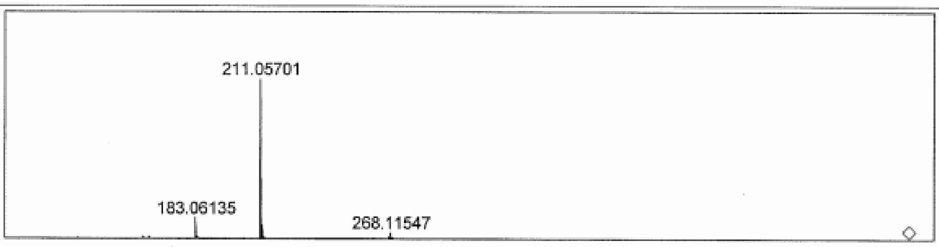

+MS2(495.10001), 33eV, 2.5min #(151)

#	m/z	I	I %
1	110.02589	159	0.1
2	115.05473	352	0.2
3	132.05576	757	0.5
4	133.05098	280	0.2
5	138.05643	696	0.5
6	160.05273	2018	1.3
7	161.06014	888	0.6
8	163.05514	1835	1.2
9	164.05916	379	0.3
10	168.03564	178	0.1
11	173.05888	231	0.2
12	183.06135	19446	12.9
13	184.06499	1775	1.2
14	191.05878	172	0.1
15	211.05701	151034	100.0
16	212.06077	13314	8.8
17	213.06214	970	0.6
18	216.09922	160	0.1
19	245.12932	476	0.3
20	246.11842	207	0.1
21	248.10816	272	0.2
22	267.13218	1771	1.2
23	268.11547	5885	3.9
24	269.11980	897	0.6
25	285.14102	233	0.2
26	494.56398	280	0.2

Acquisition Parameter

Source Type	ESI	Ion Polarity	Positive	Set Nebulizer	0.4 Bar		
Focus	Not active	Set Capillary	4500 V	Set Dry Heater	180 °C		
Scan Begin	50 m/z	Set End Plate Offset	-500 V	Set Dry Gas	4.0 l/min		
Scan End	950 m/z	Set Collision Cell RF	150.0 Vpp	Set Divert Valve	Source		

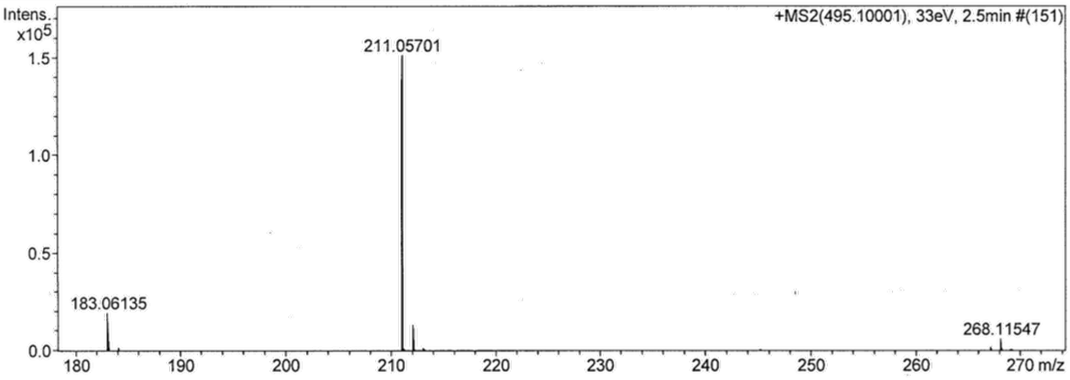

Meas. m/z	#	Formula	m/z	err [ppm]	Mean err [ppm]	rdb	N-Rule	e⁻ Conf	mSigma
183.06135	1	C 10 H 9 F 2 O	183.06160	1.4	1.2	5.5	ok	even	10.2
211.05701	1	C 11 H 9 F 2 O 2	211.05651	-2.3	-2.4	6.5	ok	even	18.0
268.11547	1	C 14 H 16 F 2 N O 2	268.11436	-4.1	-4.6	6.5	ok	even	9.0

Compound **11m**

Acquisition Parameter

Source Type	ESI	Ion Polarity	Positive	Set Nebulizer	0.4 Bar
Focus	Not active	Set Capillary	4500 V	Set Dry Heater	180 °C
Scan Begin	50 m/z	Set End Plate Offset	-500 V	Set Dry Gas	4.0 l/min
Scan End	950 m/z	Set Collision Cell RF	150.0 Vpp	Set Divert Valve	Source

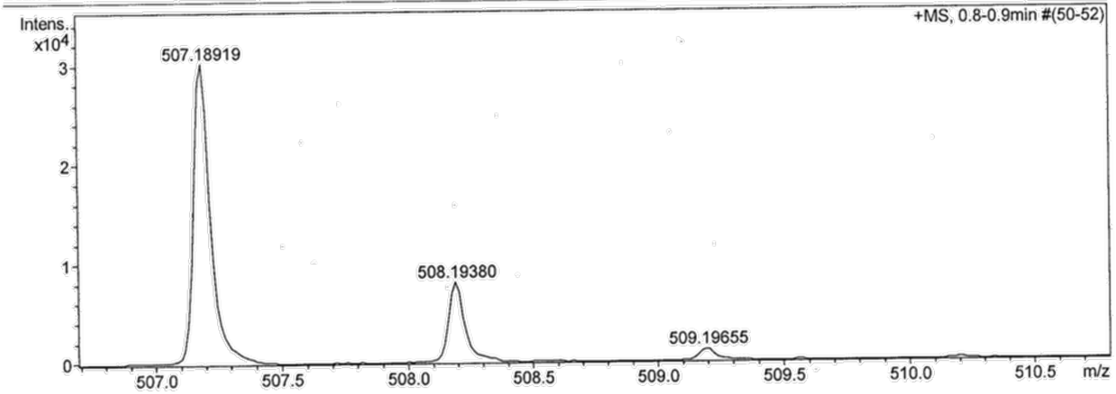

Meas. m/z	#	Formula	m/z	err [ppm]	Mean err [ppm]	rdb	N-Rule	e⁻ Conf	mSigma
507.18919	1	C 26 H 27 F 4 N 2 O 4	507.19015	1.9	1.3	12.5	ok	even	12.5

Acquisition Parameter

Source Type	ESI	Ion Polarity	Positive	Set Nebulizer	0.4 Bar
Focus	Not active	Set Capillary	4500 V	Set Dry Heater	180 °C
Scan Begin	50 m/z	Set End Plate Offset	-500 V	Set Dry Gas	4.0 l/min
Scan End	950 m/z	Set Collision Cell RF	150.0 Vpp	Set Divert Valve	Source

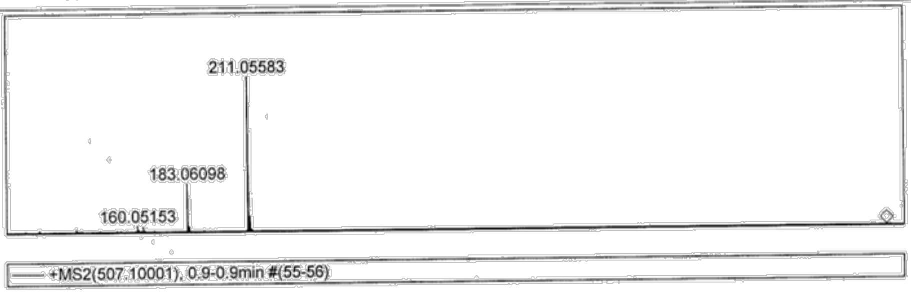

#	m/z	I	I %
1	115.05271	402	0.9
2	132.05601	674	1.5
3	133.05250	182	0.4
4	135.05993	145	0.3
5	160.05153	1664	3.6
6	161.05786	536	1.2
7	163.05407	1472	3.2
8	164.06045	220	0.5
9	183.06098	14092	30.7
10	184.06439	1438	3.1
11	191.05127	164	0.4
12	211.05583	45918	100.0
13	212.05924	4311	9.4
14	213.06362	372	0.8

Acquisition Parameter

Source Type	ESI	Ion Polarity	Positive	Set Nebulizer	0.4 Bar
Focus	Not active	Set Capillary	4500 V	Set Dry Heater	180 °C
Scan Begin	50 m/z	Set End Plate Offset	-500 V	Set Dry Gas	4.0 l/min
Scan End	950 m/z	Set Collision Cell RF	150.0 Vpp	Set Divert Valve	Source

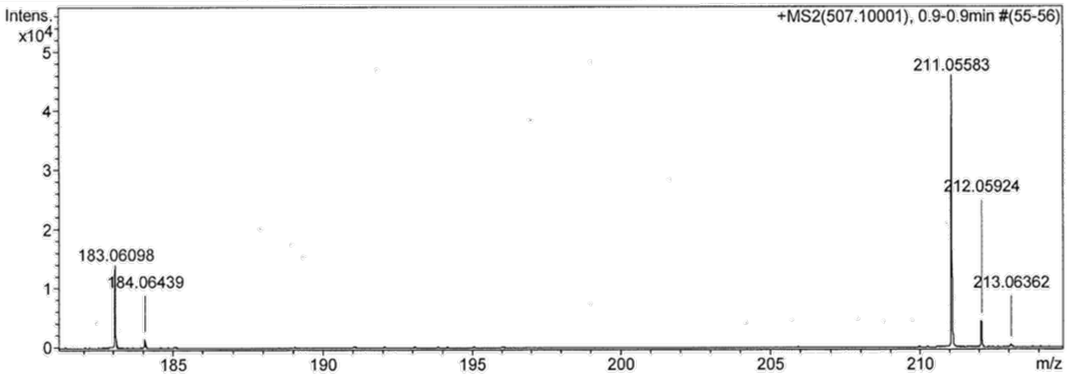

Meas. m/z	#	Formula	m/z	err [ppm]	Mean err [ppm]	rdb	N-Rule	e⁻ Conf	mSigma
183.06098	1	C 10 H 9 F 2 O	183.06160	3.4	3.4	5.5	ok	even	4.7
211.05583	1	C 11 H 9 F 2 O 2	211.05651	3.2	3.2	6.5	ok	even	14.6

Compound **13a**

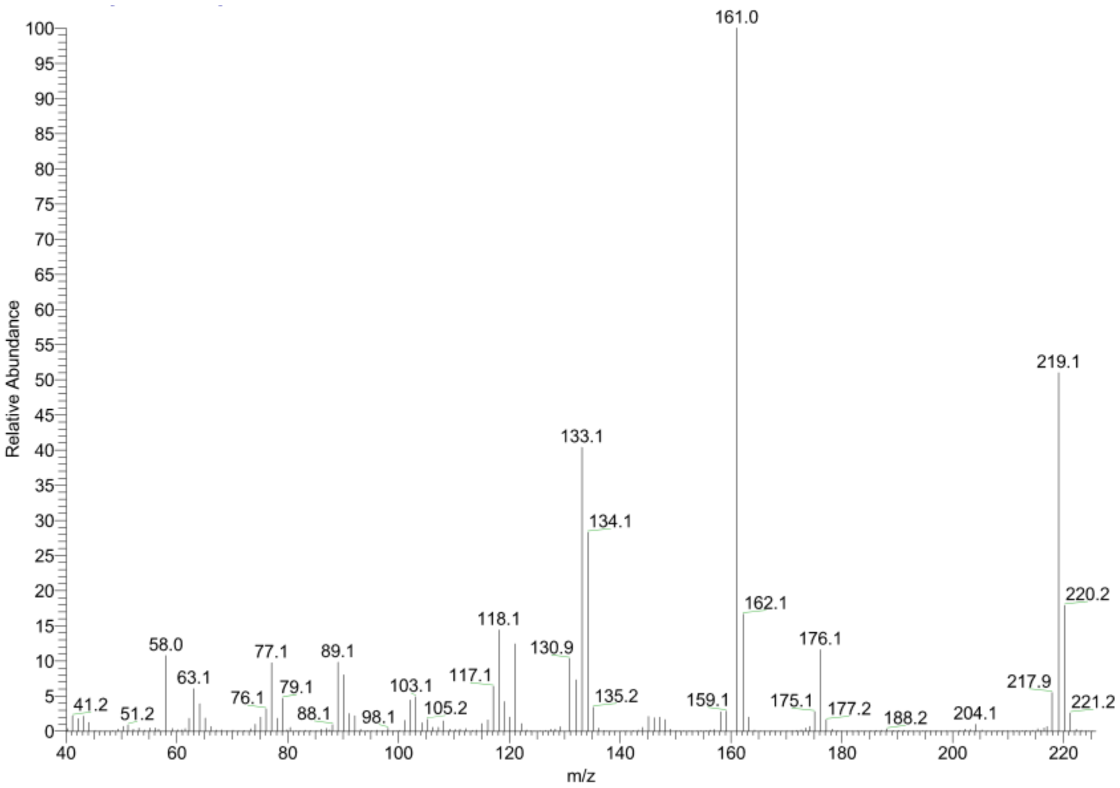

Compound **13b**

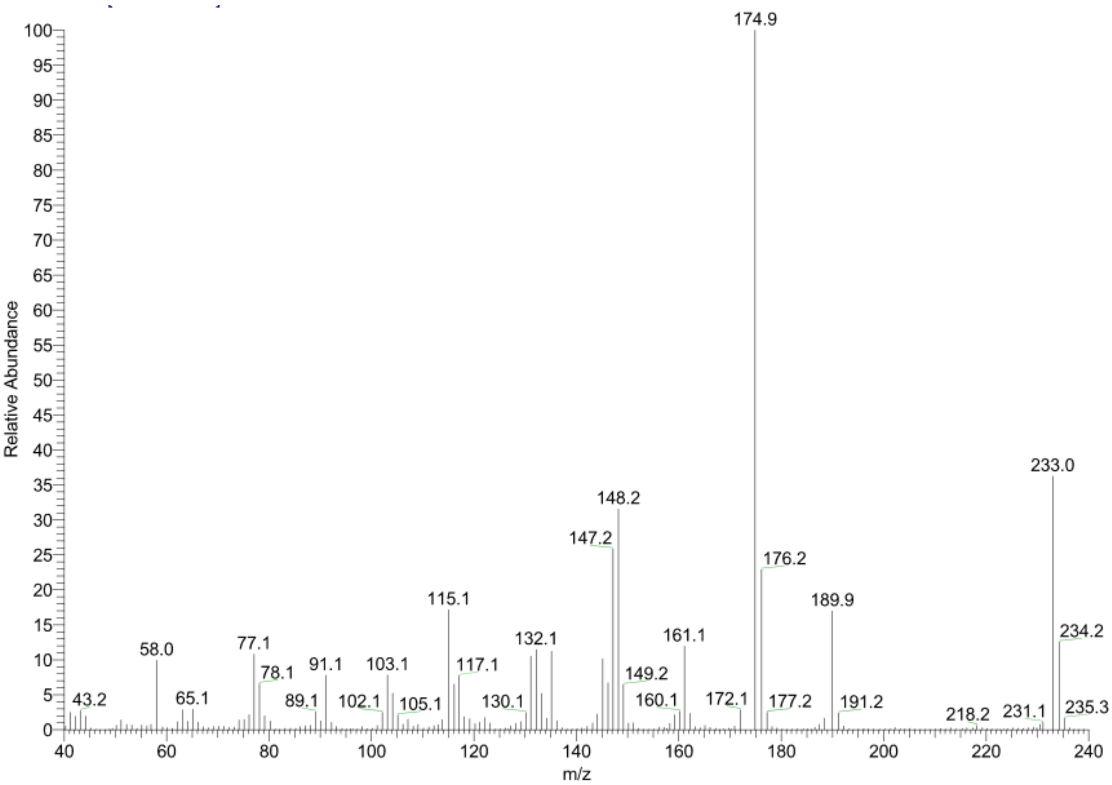

Compound **13c**

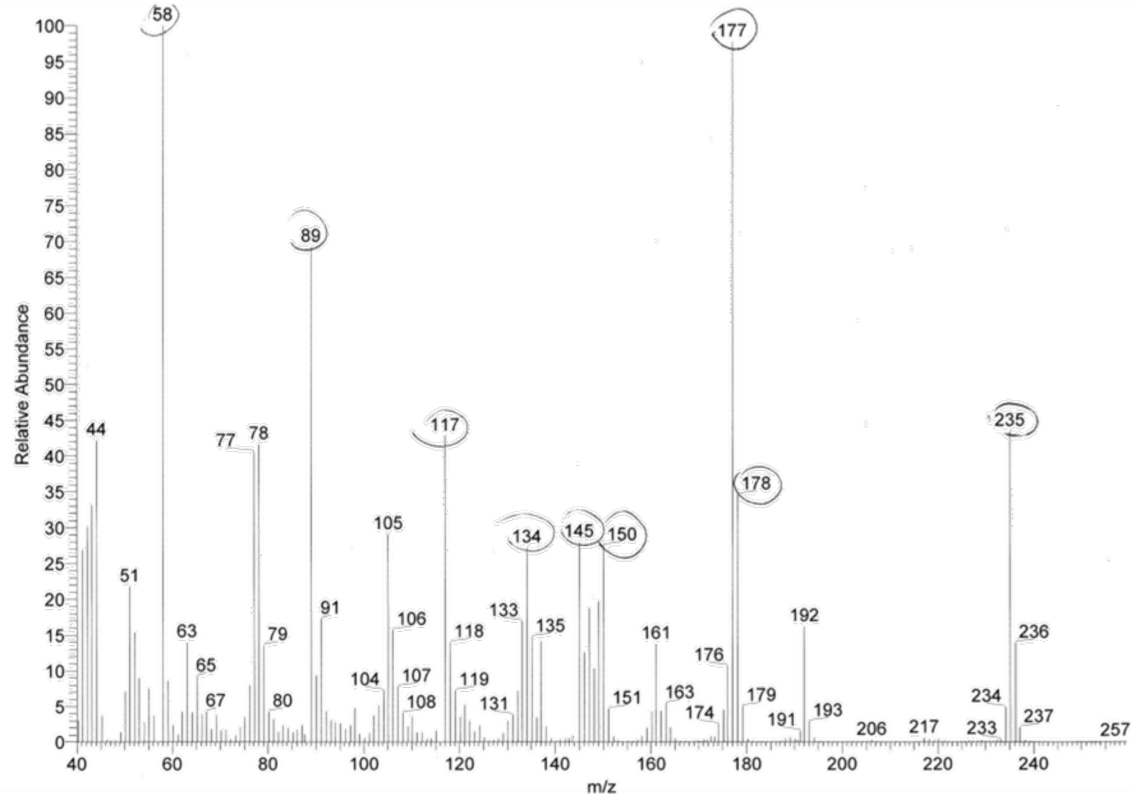

Compound **15**

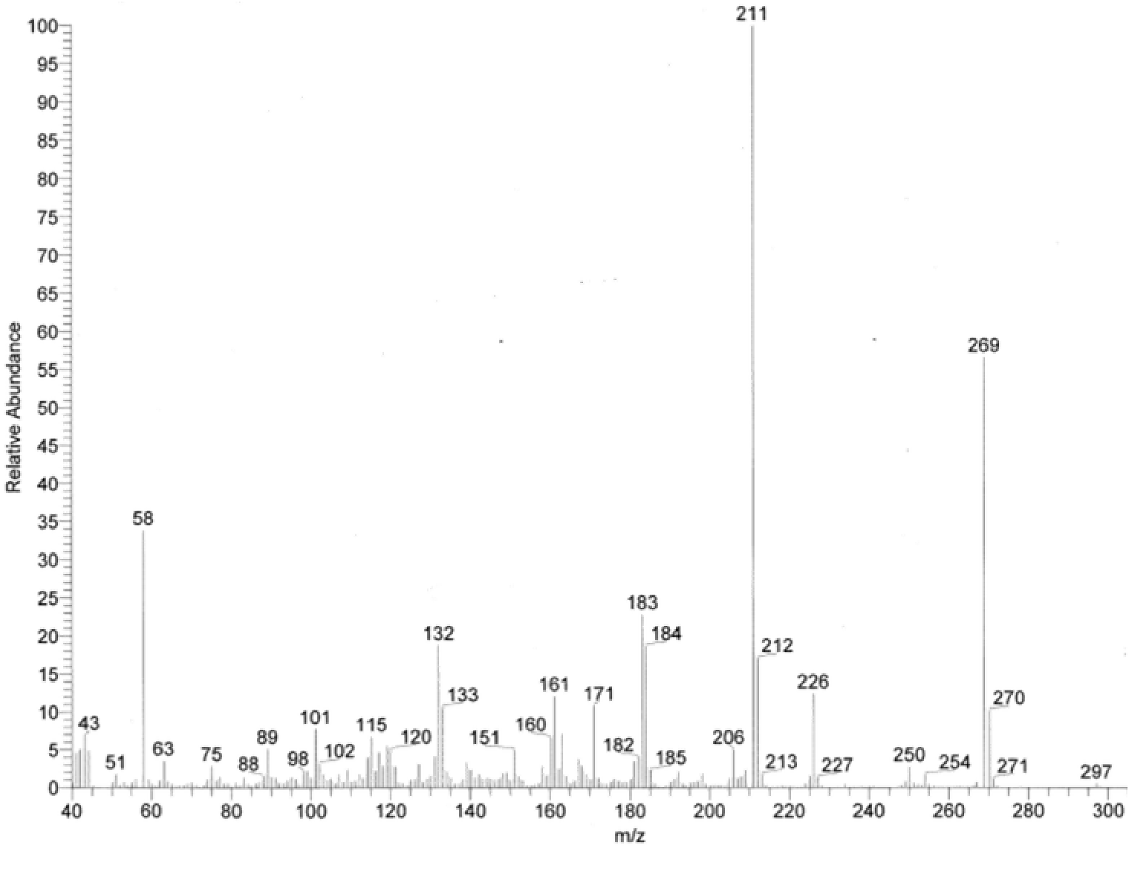

Compound **17**

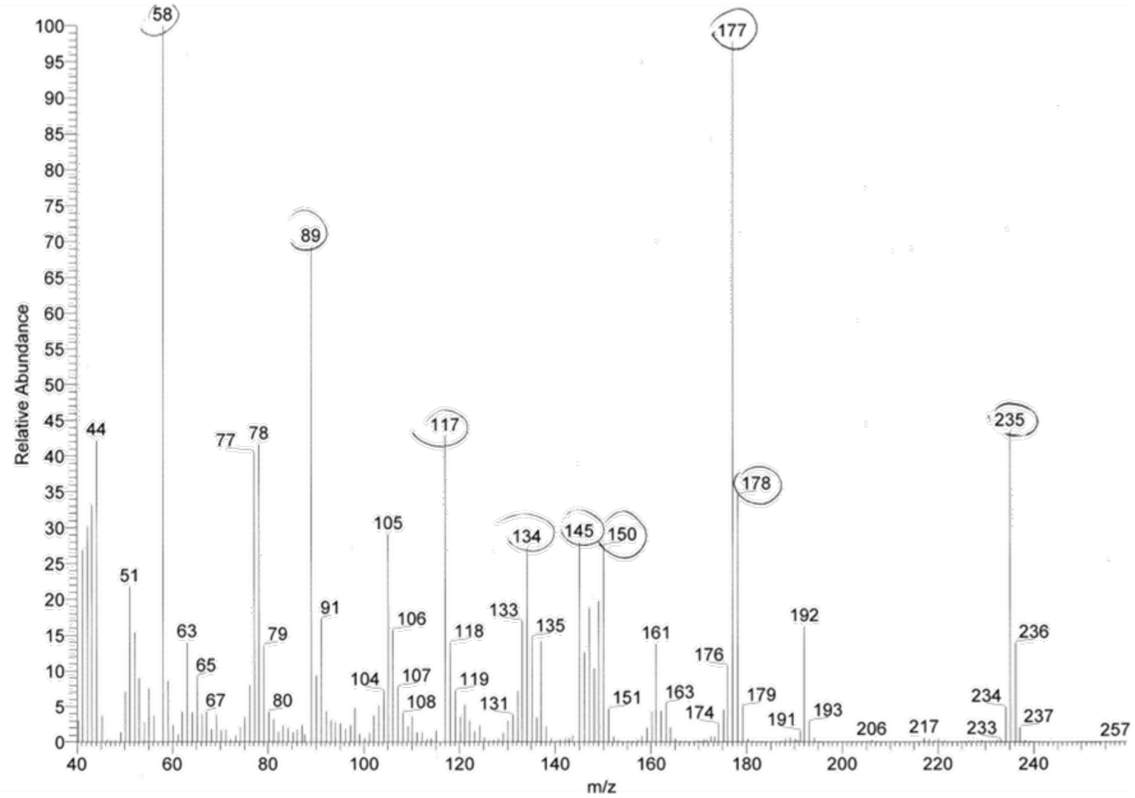

Compound **18a**

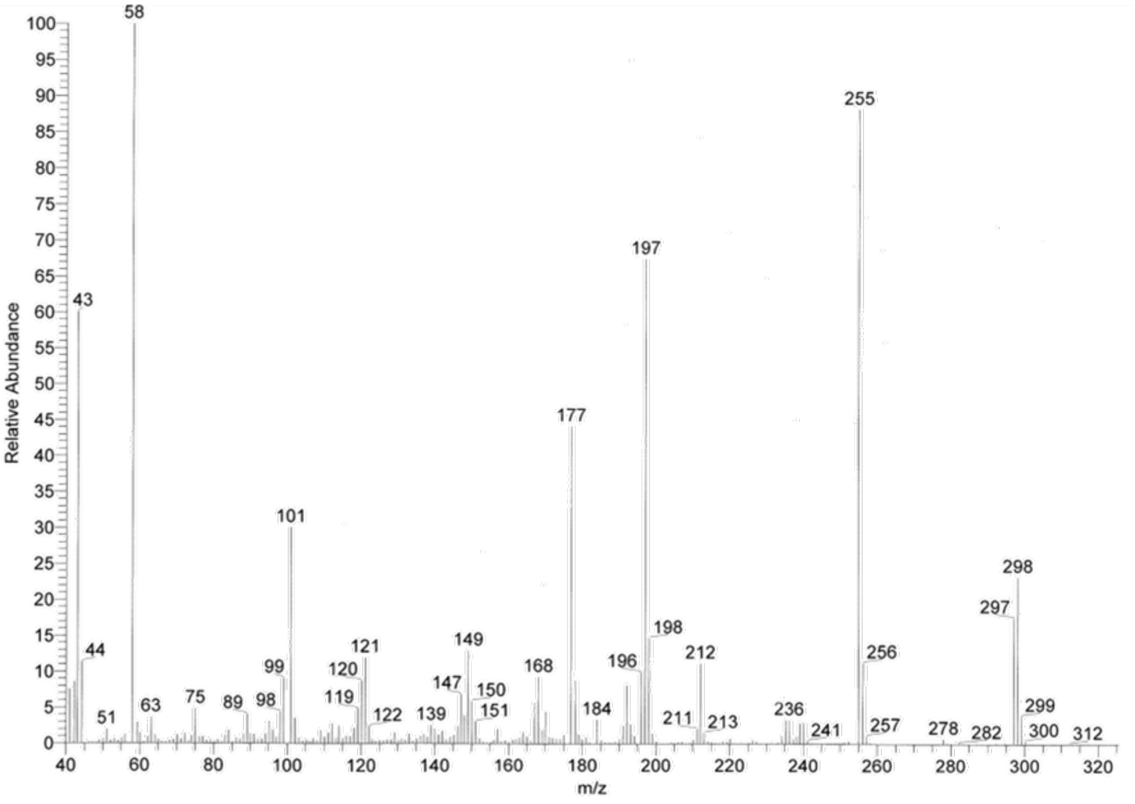

Compound **18b**

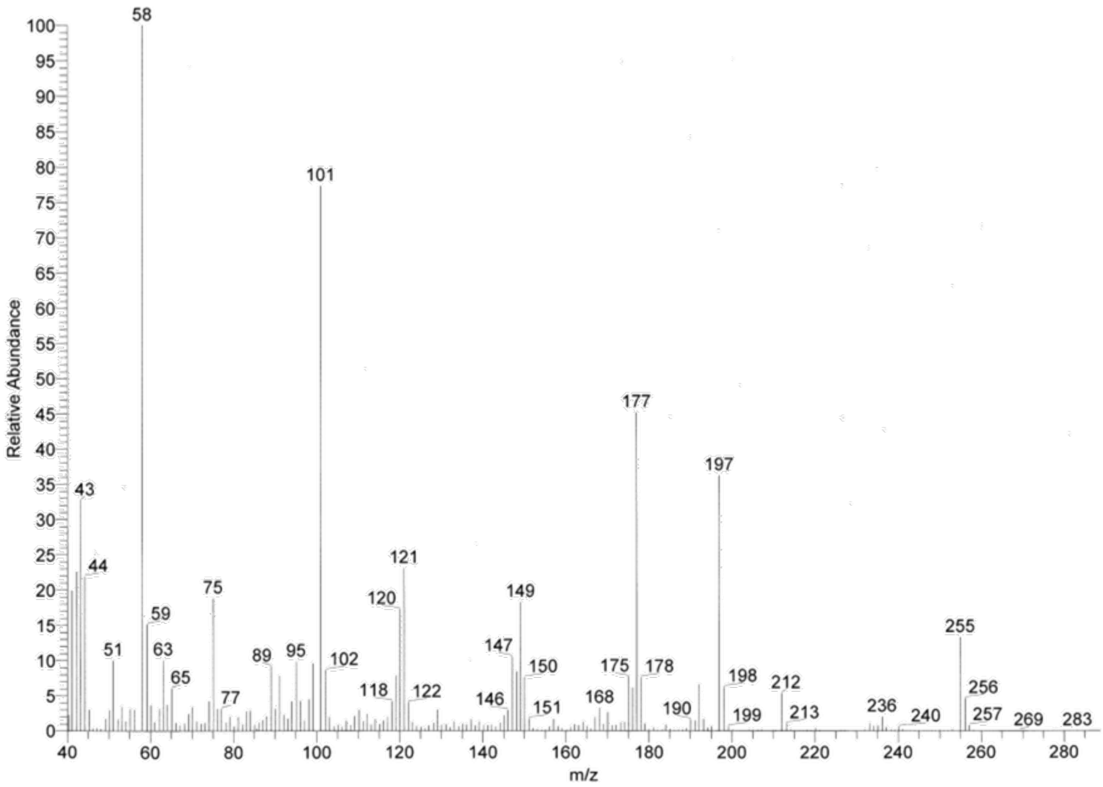

Compound **19**

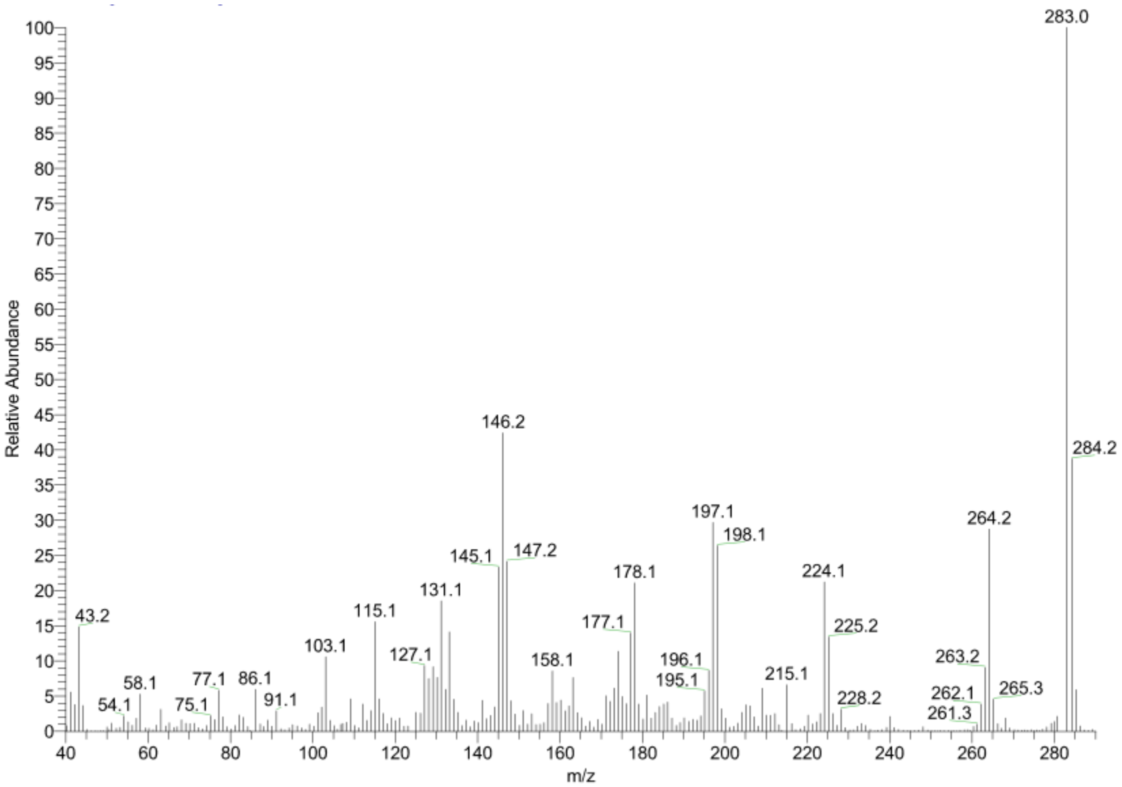

Compound **20**

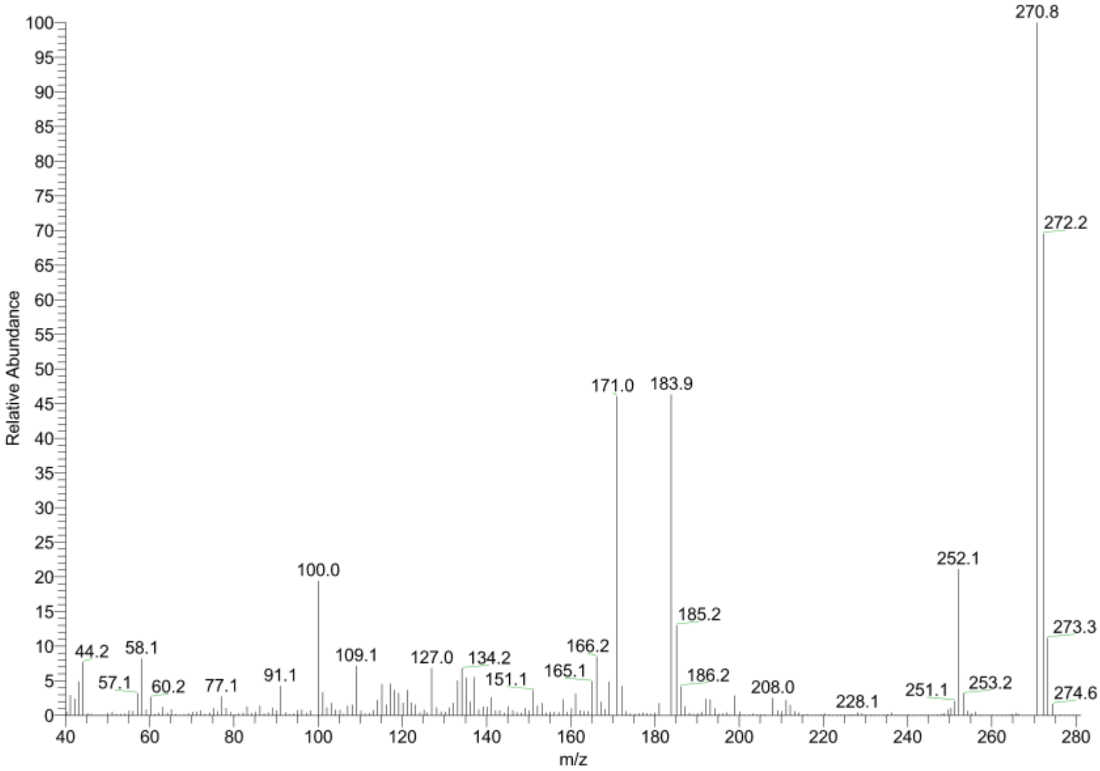

Compound **21**

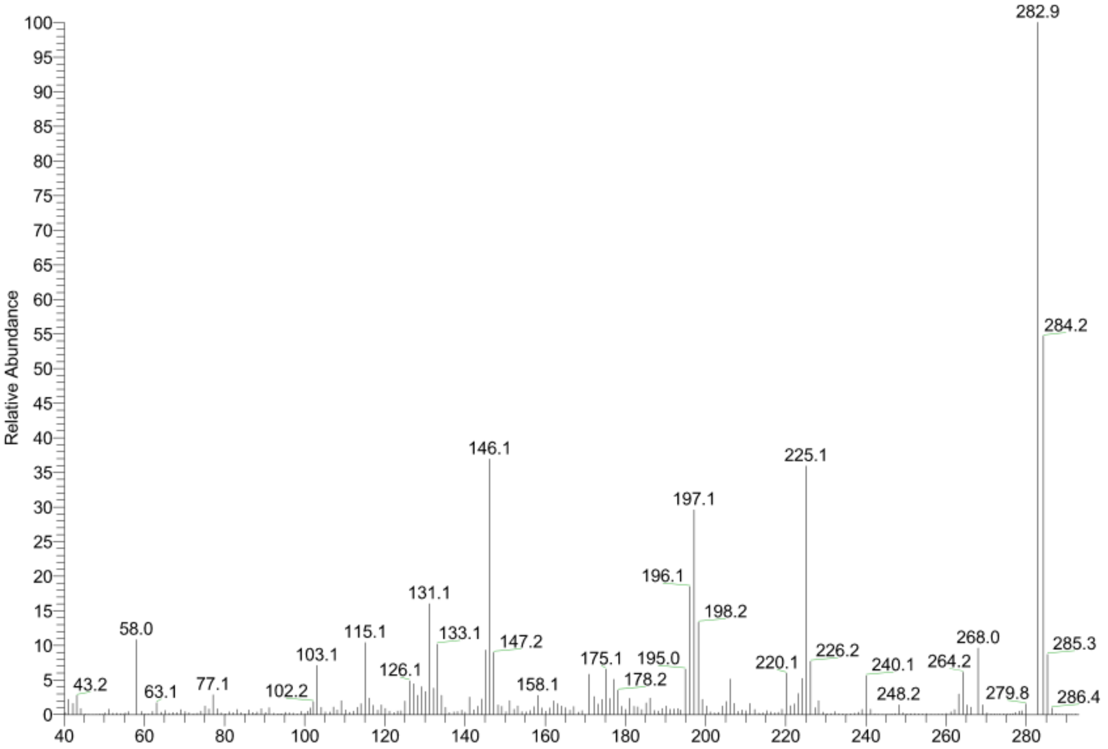

Compound **22**

Acquisition Parameter

Source Type	ESI	Ion Polarity	Positive	Set Nebulizer	0.4 Bar
Focus	Not active	Set Capillary	4500 V	Set Dry Heater	200 °C
Scan Begin	50 m/z	Set End Plate Offset	-450 V	Set Dry Gas	4.0 l/min
Scan End	950 m/z	Set Collision Cell RF	150.0 Vpp	Set Divert Valve	Source

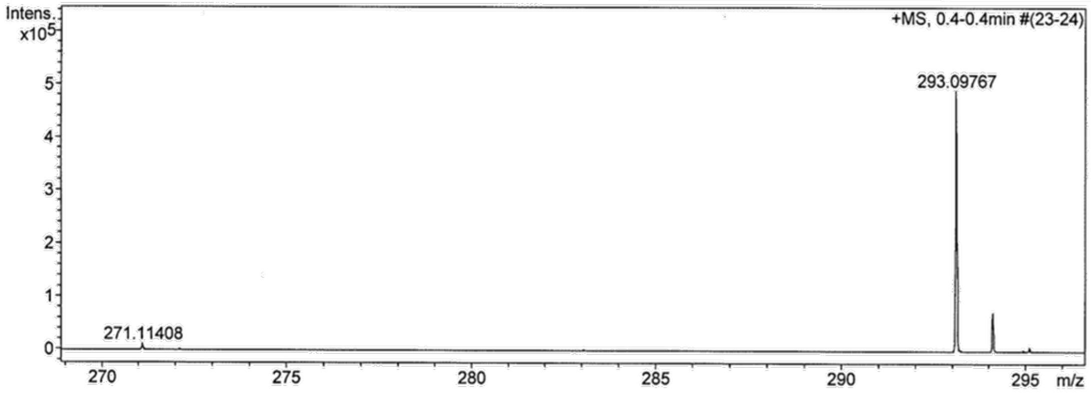

Meas. m/z	#	Formula	m/z	err [ppm]	Mean err [ppm]	rdb	e⁻ Conf	N-Rule	mSigma
271.11408	1	C 14 H 17 F 2 O 3	271.11403	-0.21	-0.10	5.5	even	ok	9.7
293.09767	1	C 14 H 16 F 2 Na O 3	293.09597	-5.79	-5.21	5.5	even	ok	2.5

Compound **23**

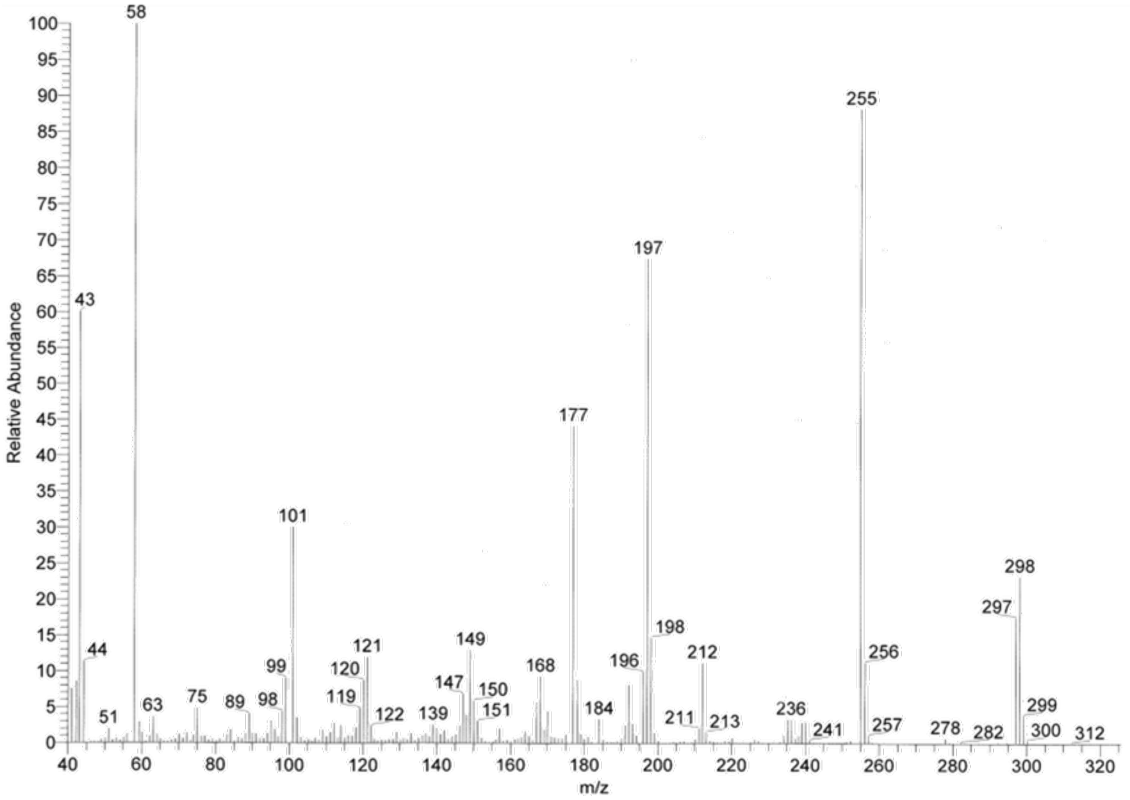

Compound **24**

Acquisition Parameter

Source Type	ESI	Ion Polarity	Positive	Set Nebulizer	3.0 Bar
Focus	Not active	Set Capillary	4000 V	Set Dry Heater	200 °C
Scan Begin	100 m/z	Set End Plate Offset	-500 V	Set Dry Gas	7.0 l/min
Scan End	1100 m/z	Set Collision Cell RF	150.0 Vpp	Set Divert Valve	Source

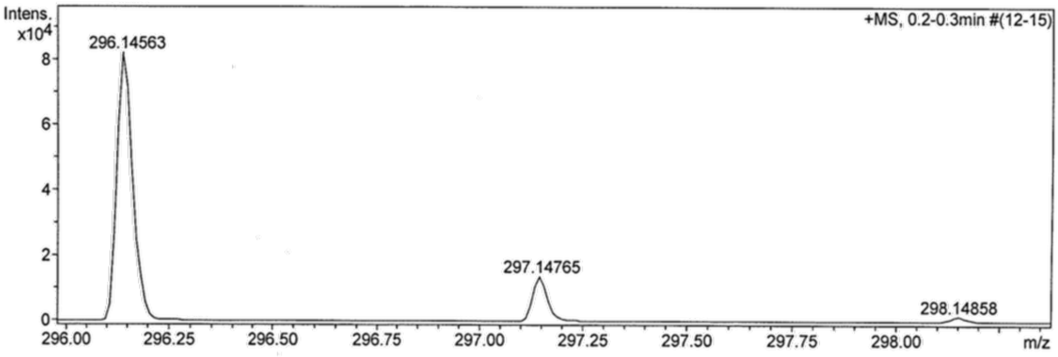

Meas. m/z	#	Formula	m/z	err [ppm]	Mean err [ppm]	e⁻ Conf	N-Rule	mSigma
296.14563	1	C 16 H 20 F 2 N O 2	296.14566	0.12	0.93	even	ok	6.0

Compound **25**

Acquisition Parameter FCEN - UBA

Source Type	ESI	Ion Polarity	Positive	Set Nebulizer	3.0 Bar
Focus	Not active	Set Capillary	4000 V	Set Dry Heater	200 °C
Scan Begin	100 m/z	Set End Plate Offset	-500 V	Set Dry Gas	7.0 l/min
Scan End	1100 m/z	Set Collision Cell RF	150.0 Vpp	Set Divert Valve	Source

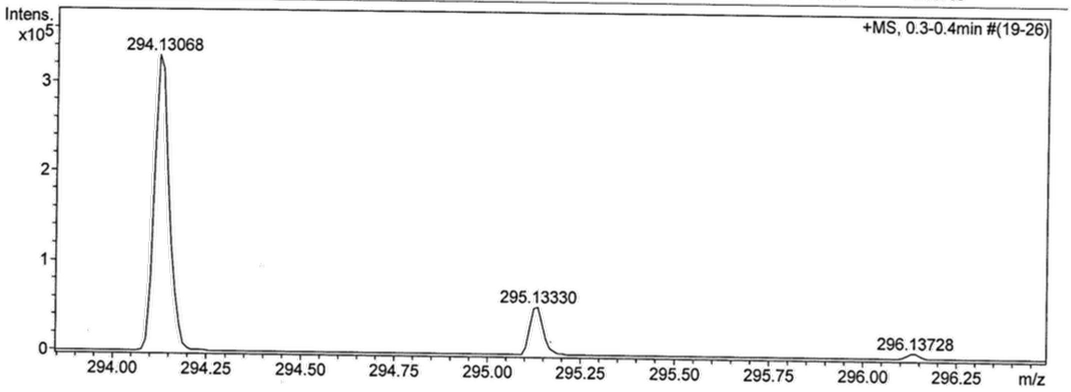

Meas. m/z	#	Formula	m/z	err [ppm]	Mean err [ppm]	e⁻ Conf	N-Rule	mSigma
294.13068	1	C 16 H 18 F 2 N O 2	294.13001	-2.27	-1.93	even	ok	8.1

Compound **S2**

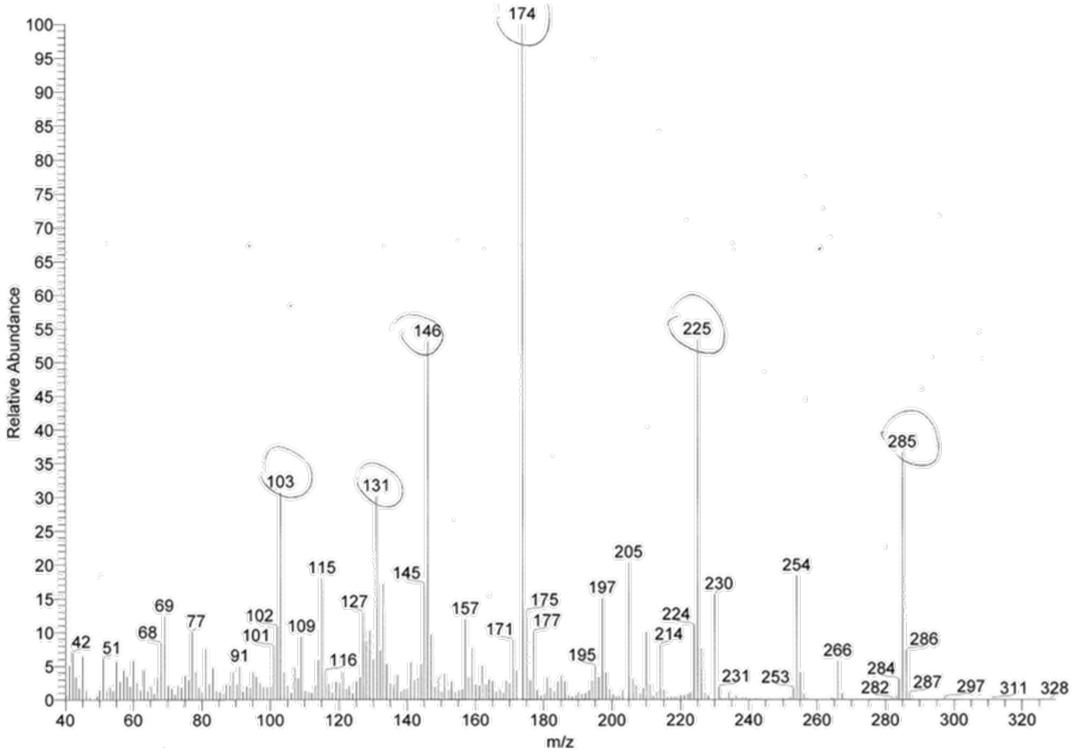

Relative Abundance
m/z
174
146
225
285
103
131
205
254
115
145
230
197
69
127
157
175
102
224
77
109
171
177
68
101
214
286
42
51
91
116
195
266
284
231
253
287
297
311
328
282
